# The septin cytoskeleton is a regulator of intestinal epithelial barrier integrity and mucosal inflammation

**DOI:** 10.1172/jci.insight.191538

**Published:** 2025-10-07

**Authors:** Nayden G. Naydenov, Gaizun Hu, Dominik Robak, Atif Zafar, Khosiyat Makhmudova, Susana Lechuga, Yuta Ohno, Naseer Sangwan, Saikat Bandyopadhyay, Ryan Musich, Erin Jeffery, Lei Sun, Armando Marino-Melendez, Florian Rieder, Gloria Sheynkman, Andrei I. Ivanov, Seham Ebrahim

**Affiliations:** 1 Department of Inflammation and Immunity, Lerner Research Institute, Cleveland Clinic Foundation, Cleveland, Ohio, USA.; 2 Center for Membrane and Cell Physiology, University of Virginia, Charlottesville, Virginia, USA.; 3 Department of Molecular Physiology and Biological Physics, University of Virginia School of Medicine, Charlottesville, Virginia, USA.; 4 Department of Cardiovascular and Metabolic Science, Lerner Research Institute, and; 5 Department of Gastroenterology, Hepatology and Nutrition, Digestive Diseases Institute, Cleveland Clinic Foundation, Cleveland, Ohio, USA.

**Keywords:** Cell biology, Gastroenterology, Cytoskeleton, Inflammatory bowel disease, Tight junctions

## Abstract

Intestinal epithelial barrier integrity is essential for human health, and its disruption induces and exacerbates intestinal inflammatory disorders. While the epithelial cytoskeleton is critical for maintaining gut barrier-integrity, the role of septins — a family of GTP-binding, cytoskeletal proteins — is largely unknown. This highlights an important knowledge gap, as dysfunction of septins, and specifically septin 9 (SEPT9), is associated with intestinal pathologies. We determined that SEPT9 localizes to the apical junctions of intestinal epithelial cells (IECs), overlapping with both tight and adherens junctions. IEC-specific ablation of SEPT9 in mice resulted in leaky gut, due to mislocalization of junctional proteins, and increased susceptibility to experimental colitis. Consistently, SEPT9 expression was significantly reduced in intestinal mucosa of patients with inflammatory bowel disease (IBD). Using affinity-purification mass spectrometry, super-resolution imaging, and genetic KO, we determined that SEPT9 interacts with and is necessary to recruit nonmuscle myosin IIC (NMIIC) to the IEC perijunctional actomyosin belt. Loss of NMIIC also caused IEC barrier disruption. In summary, SEPT9 regulates intestinal barrier integrity by supporting the assembly of tight and adherens junctions through NMIIC recruitment to the actomyosin belt. The septin cytoskeleton safeguards the intestinal mucosa during acute inflammation, and its disruption in IBD suggests a loss of this protective function.

## Abstract 2

The septin cytoskeleton localizes to apical junctions of intestinal epithelial cells and maintains barrier integrity, thus protecting the intestinal epithelium from inflammation

## Graphical abstract

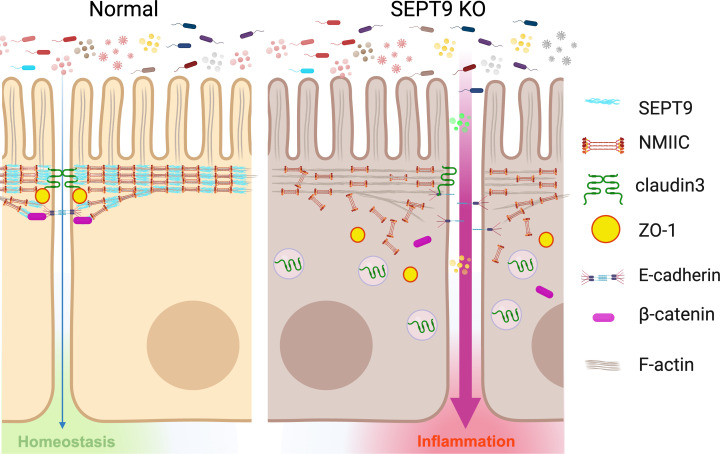

## Introduction

The intestinal epithelial barrier is a critical component of a healthy gut, forming a protective boundary that separates internal organs from luminal contents, including microorganisms, while regulating the directional flow of water, nutrients, and waste. Disruption of this barrier is a hallmark of various gastrointestinal disorders, most notably inflammatory bowel diseases (IBD) such as Crohn’s disease (CD) and ulcerative colitis (UC) (1–3). These barrier defects are key drivers of disease progression, triggering and sustaining mucosal inflammation (3–5). The gut barrier is composed of a single layer of columnar epithelial cells interconnected by lateral cell-cell adhesions, mediated by specialized multiprotein complexes known as epithelial junctions. The 3 primary types of epithelial junctions are tight junctions (TJs), adherens junctions (AJs), and desmosomes. Among these, the apically located TJs play a central role in maintaining epithelial barrier integrity and regulating permeability, underscoring their significance in gut health and disease (6–9).

TJs are formed via self-assembly of various transmembrane proteins, including claudins, occludin, and junctional adhesion molecule A (6–8). These proteins interact with counterparts on adjacent plasma membranes, establishing a selective barrier. On their cytoplasmic side, TJs are anchored by members of the *zonula occludens* (ZO) protein family, along with other scaffolding, polarity, and signaling proteins (6–8). TJs work in tandem with E-cadherin–based AJs to regulate epithelial barrier integrity and facilitate intestinal mucosal repair (9, 10). A defining feature of TJs and AJs is their coupling to the circumferential actomyosin belt, composed of antiparallel actin filament bundles and nonmuscle myosin II (NMII) motors (11–13). This connection to the actomyosin cytoskeleton is essential for maintaining junction stability, regulating permeability, and enabling dynamic remodeling of intercellular contacts (11–13).

Interestingly, superresolution microscopy has revealed that the perijunctional NMII assembles into periodic, sarcomere-like structures (14, 15), suggesting a critical role in mechanical force transduction and the regulation of junctional permeability (16). Despite these insights, the mechanisms governing the recruitment and assembly of the junction-associated actomyosin cytoskeleton, particularly in healthy versus inflamed intestinal epithelium, remain poorly understood.

Septin GTPases are a family of filament-forming proteins with the distinctive ability to interact with cellular membranes and major cytoskeletal components, such as actin filaments and microtubules (17–19). Septins have been reported to cross-link and curve actin filaments (20, 21), anchor actin bundles to the plasma membrane (22), and interact with NMII and regulate its activity (21, 23). These properties make the septin cytoskeleton a compelling candidate for controlling the assembly and contractility of the perijunctional actomyosin belt, thereby playing a potential role in maintaining epithelial barrier integrity. While some septins were previously implicated in the regulation of epithelial and endothelial junctions in vitro (24–26), the underlying molecular mechanisms and in vivo relevance of the septin-dependent control of tissue barrier integrity remain enigmatic.

The septin cytoskeleton in mammalian cells is formed by 12 homologous members of this protein family that copolymerize into either octamers or hexamers, with subsequent assembly into high-order cytoskeletal elements (22). Among them, septin 9 (SEPT9) plays a major role in formation of mammalian septin octamers and their interactions with other cellular structures (22). Importantly, several studies have reported associations between SEPT9 dysregulation and human intestinal disorders. Both mutations and hypermethylation of the SEPT9 gene, resulting in reduced protein expression, occur in IBD and colorectal cancer (27–29). Since colon carcinogenesis is known to be modulated by gut inflammation and inflammation-induced disruption of the intestinal epithelial barrier (30, 31), it is important to elucidate how the SEPT9-based cytoskeleton regulates the intestinal epithelial barrier integrity under homeostatic conditions and in mucosal inflammatory disorders.

In this study, we investigate the cellular localization and function of SEPT9 in the intestinal epithelium using different experimental approaches, including knockin mice expressing fluorescently tagged endogenous SEPT9, epithelial-specific SEPT9-KO mice, and SEPT9-deficient human intestinal epithelial cells (IECs). We demonstrate, for the first time to our knowledge, that SEPT9 is enriched at epithelial apical junctions, colocalizing with TJ and AJ proteins, and regulates intestinal epithelial barrier integrity via the recruitment of junctional NMIIC and assembly of TJ components. Furthermore, SEPT9 plays protective roles during experimental colitis in vivo by limiting mucosal injury and inflammation. Consistent with these findings, epithelial SEPT9 expression is diminished in the intestinal mucosa of patients with IBD, and it is mislocalized in IECs of patients with CD. Our findings, thus, reveal a potentially new role for the septin cytoskeleton in regulating epithelial junctional integrity in vivo, unravel the mechanism underlying the barrier-protective function of SEPT9, and highlight that abnormal organization of the septin cytoskeleton in patients with IBD could be an important contributor to chronic mucosal inflammation.

## Results

### SEPT9 is expressed in different subtypes of IECs and localizes at epithelial junctions.

**Figure 1:**
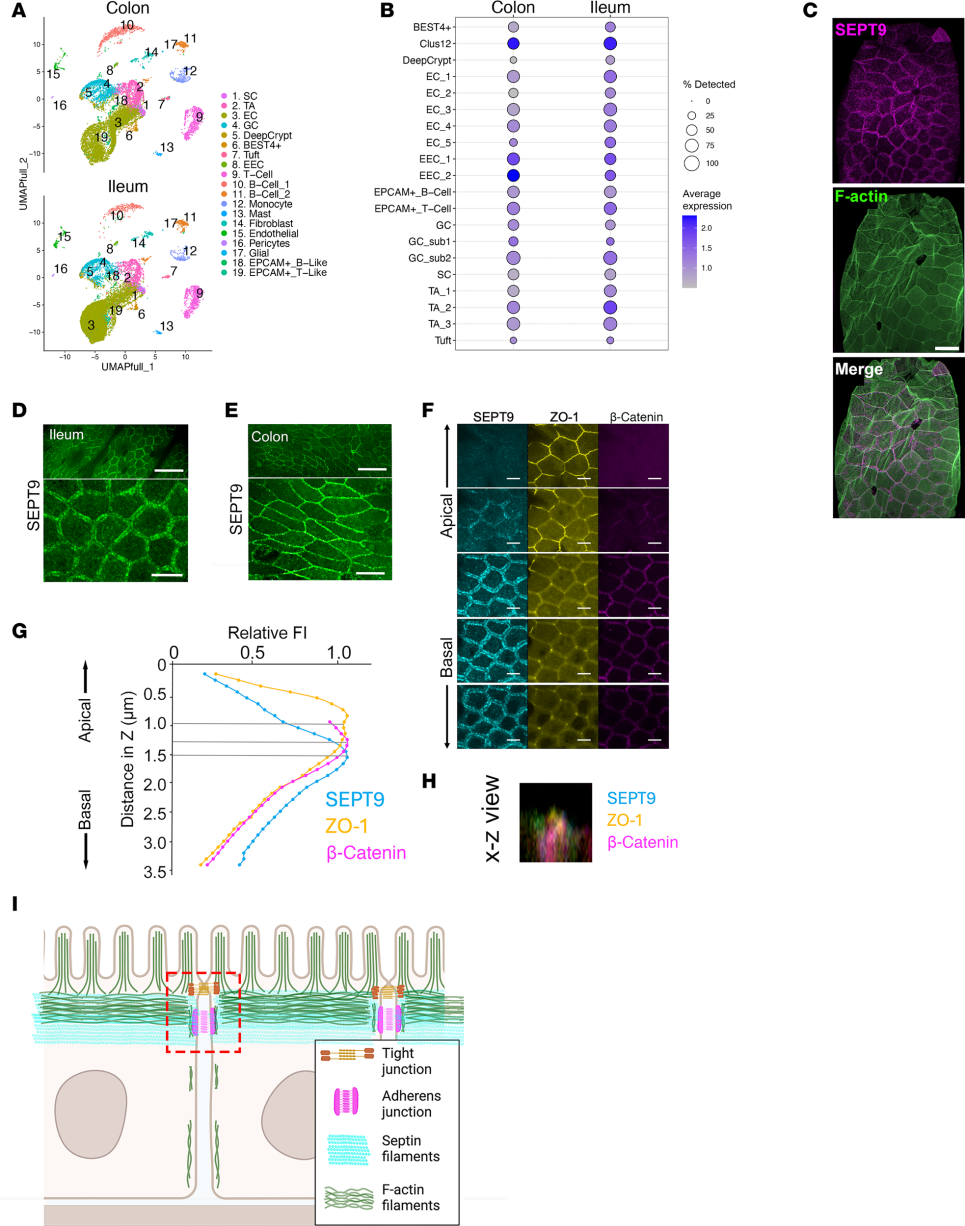
SEPT9 is enriched at epithelial cell-cell junctions in the intestine and overlaps with tight and adherens junctions. (**A**) UMAP plots showing only SEPT9-expressing cells (raw count > 0) from single-cell RNA-Seq of nondiseased human ascending colon and terminal ileum paired biopsies. Clusters corresponding to different populations of epithelial and nonepithelial cells are indicated and color coded. (**B**) Dot plot of SEPT9 transcript expression across annotated epithelial cell subtypes in human colon and ileum. Dot size reflects percentage of cells expressing SEPT9; color indicates average expression level. SEPT9 expression is enriched in several cell clusters (especially in EC and TA cells). (**C**) Confocal image of mouse small intestinal epithelium labeled for endogenous SEPT9 (magenta), F-actin (green), and merged image, showing junctional colocalization. Scale bar: 20 μm. (**D** and **E**) En face images of SEPT9 immunolabeling in ileal (**D**) and colonic (**E**) intestinal epithelium show continuous, junctional localization in differentiated epithelial cells. Scale bars: 50 μm (upper panels) and 10 μm (lower panels). (**F**) High-resolution confocal *Z*-stack images of ileal epithelial junctions showing localization of SEPT9 (cyan), ZO-1 (yellow), and β-catenin (magenta) in apical and basal optical sections. SEPT9 is enriched between tight (ZO-1) and adherens (β-catenin) junctions. Scale bars: 5 μm. (**G**) Quantification of fluorescence intensity profiles across the apical/basal axis shows that SEPT9 peaks between ZO-1 and β-catenin signals (*n* = 10 junctions from 3 mice). (**H**) Orthogonal (*x*–*z*) view of confocal stack at a position equivalent to dotted red box in (**I**), showing SEPT9-NG (cyan) localization relative to ZO-1 (yellow) and β-catenin (magenta). (**I**) Schematic model summarizing SEPT9 localization at epithelial junctions, associating with tight and adherens junctions and the cortical actin cytoskeleton.

A publicly available single-cell RNA dataset (GSE266616) was used to examine SEPT9 expression in different cell types in healthy human ileum and colon. SEPT9 was expressed in all major cell populations, including epithelial, immune, and stromal cells (Figure 1A). A deeper examination of different IEC subtypes revealed SEPT9 expression in all major differentiated and progenitor IECs in normal ileal and colonic mucosa being especially abundant in absorptive enterocytes (EC3, EC4), enterochromaffin cells (EEC1, EEC2), transient amplified cells (TA1-3), and an unidentified IEC population (Clus12) (Figure 1B). SEPT9 mRNA levels show positive correlation with the expression of other major septins, SEPT2, SEPT7, and SEPT11, in different ileal and colonic IEC subtypes (Supplemental Figure 1, A and B; supplemental material available online with this article; https://doi.org/10.1172/jci.insight.191538DS1), which could indicate coregulation and possible functional cooperation of these septin paralogs in human intestinal epithelium.

The subcellular localization of endogenous SEPT9 in mouse intestinal mucosa was evaluated by immunofluorescence labeling in WT mice, revealing that SEPT9 is enriched at the perijunctional F-actin belt (Figure 1C). We then generated CRISPR/Cas9 engineered knockin reporter mice, where an mNeonGreen fluorescent protein was tagged to the C-terminus of endogenous SEPT9. There are several SEPT9 isoforms that differ at their N-termini (17). A C-terminal tagging strategy was thus chosen to ensure that all SEPT9 isoforms are labeled. Importantly, several studies have confirmed that tagging SEPT9 at either C- or N-terminus does not affect its localization or function in cell lines (32–35). SEPT9-mNeonGreen in the intestinal epithelia of the mNeonGreen-tagged SEPT9 (SEPT9^mNG^) mice was enriched at epithelial apical junctions in both the ileum (Figure 1D) and colon (Figure 1E), supporting the likelihood that the mNeonGreen tag does not affect the localization and function of the endogenous SEPT9. We next sought to delineate the specific localization of SEPT9 relative to the IEC TJs and AJs. We immunolabeled ileal tissue from SEPT9^mNG^ mice for the major TJ protein, ZO-1, and the AJ protein, β-catenin, and imaged all 3 proteins relative to each other, using an approach used previously in inner ear epithelial tissue (14). Specifically, we acquired *Z*-stacks of confocal images of the apical junctional region of the ileal epithelium at 50 nm intervals (a subset of these images is shown in Figure 1F). To estimate the relative localization of each tagged protein along the *z* axis, we plotted the immunofluorescence intensity values and determined the points of fluorescence maxima. The immunofluorescence signal of ZO-1 along the *z* axis was most apical (Figure 1G), while β-catenin and SEPT9 were both more basal. Of note, the fluorescence intensity (FI) of SEPT9^mNG^ overlapped partially with ZO-1 and more prominently with β-catenin immunofluorescence (Figure 1, G and H, and Supplemental Figure 1C). These results are consistent with a SEPT9 network that overlaps with both TJs and AJs (Figure 1I).

### SEPT9 expression and localization are altered in the intestinal mucosa of patients with IBD.

**Figure 2:**
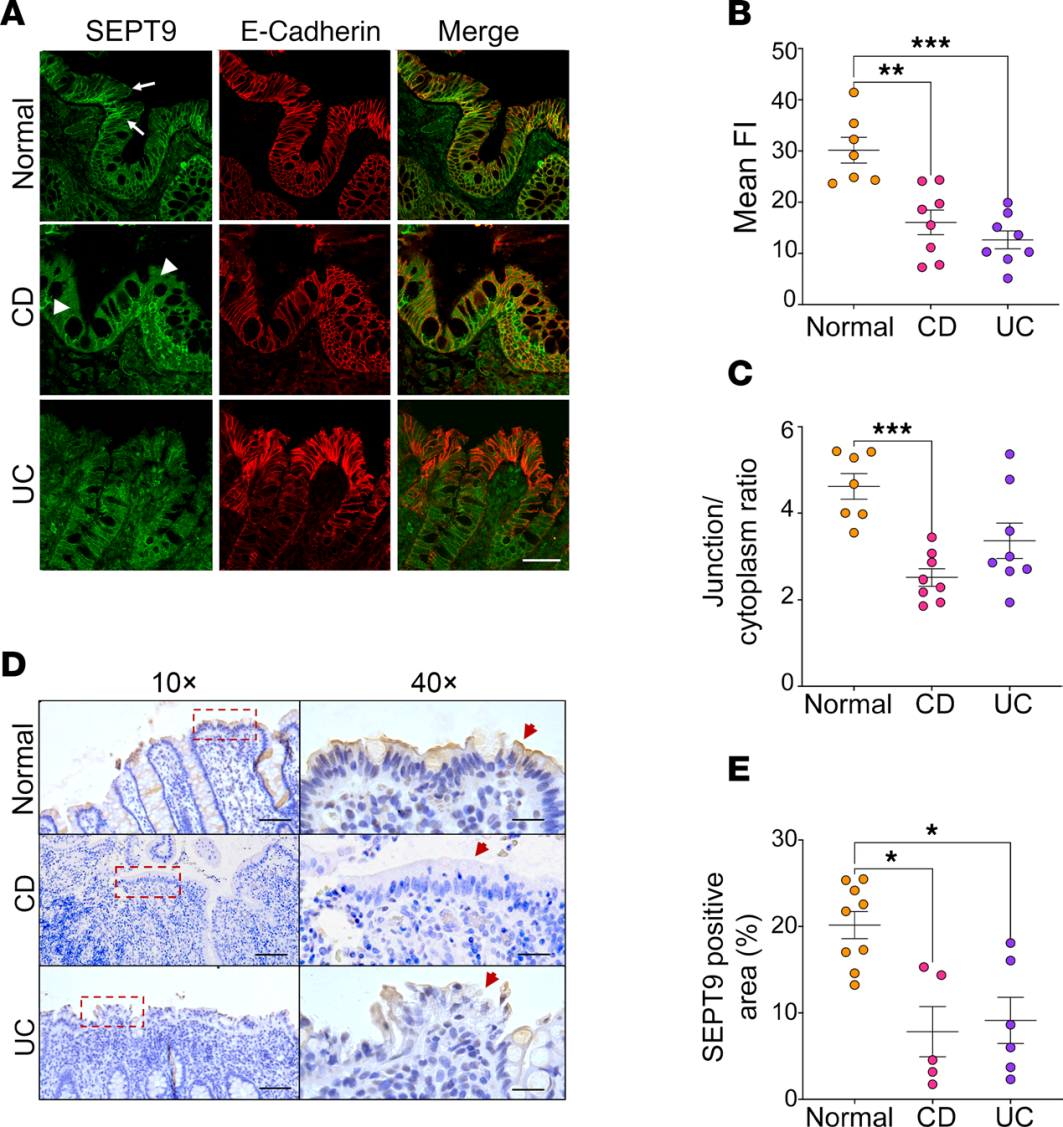
SEPT9 is downregulated and mislocalized from epithelial junctions in intestinal mucosa of patients with IBD. (**A**) Confocal images of human colonic mucosa from patients classified as healthy controls (Normal) or those with Crohn’s disease (CD) or ulcerative colitis (UC) immunolabeled for SEPT9 (green) and E-cadherin (red). In control samples, SEPT9 is prominently localized at apical junctions (arrows). In CD tissues, SEPT9 is mislocalized to the cytoplasm (arrowheads). Scale bar: 50 μm. (**B**) Quantification of mean fluorescence intensity (FI) of SEPT9 immunolabeling in normal and CD ileal and colonic tissue samples and UC colonic samples (*n* = 7–8 patients/group). (**C**) Quantification of junctional/cytoplasmic SEPT9 signal ratio across conditions (*n* = 7–8 patients/group). (**D**) Representative IHC images (10× and 40×) showing SEPT9 protein localization in colonic tissue of control and patients with IBD. Red arrowheads indicate SEPT9 signal in IECs. Dashed boxes in 10× panels denote regions shown at higher magnification. Scale bars: 50 μm (10×), 10 μm (40×). (**E**) Quantification of SEPT9^+^ epithelial area from IHC sections, showing decreased SEPT9 expression in CD and UC tissues (*n* = 5–9 patients/group). Data in **B**, **C**, and **E** are presented as mean ± SEM. *P < 0.05, **P < 0.01, ***P < 0.001 by 1-way ANOVA with Tukey’s multiple-comparison test.

Drawing from our observations in mouse tissue, where SEPT9 associates with apical epithelial junctions, we analyzed SEPT9 expression and localization in human intestinal mucosa of patients with CD or UC (Supplemental Table 1). We performed dual immunofluorescence staining for SEPT9 and E-cadherin on cryosections (Figure 2A), and IHC in paraffin-embedded sections (Figure 2D), using tissue specimens from patients with diverticulitis and normal margins of resected colorectal cancers as controls.

To guarantee accurate analysis, we focused strictly on areas with well-preserved epithelium, identified by continuous E-cadherin labeling (IF) or confirmed by adjacent PAS staining (IHC; Supplemental Figure 2). In healthy intestinal epithelium, SEPT9 was enriched at E-cadherin–labeled junctions (Figure 2A, arrows), recapitulating its junctional localization in mouse intestine (Figure 1). However, overall SEPT9 intensity was significantly reduced in ileal and colonic segments of patients with CD and in colonic samples of patients with UC (Figure 2, B and E), consistent with recent reports of SEPT9 gene methylation and downregulation in inflamed mucosa (29). Notably, junction-to-cytoplasmic SEPT9 ratios were decreased in CD intestinal mucosa (Figure 2A arrowheads and 2C), indicating diminished junctional accumulation of this protein and corroborating our hypothesis that SEPT9 integrity at apical junctions may be compromised during IBD.

### Loss of intestinal epithelial SEPT9 increases gut barrier permeability and alters junctional protein localization.

**Figure 3:**
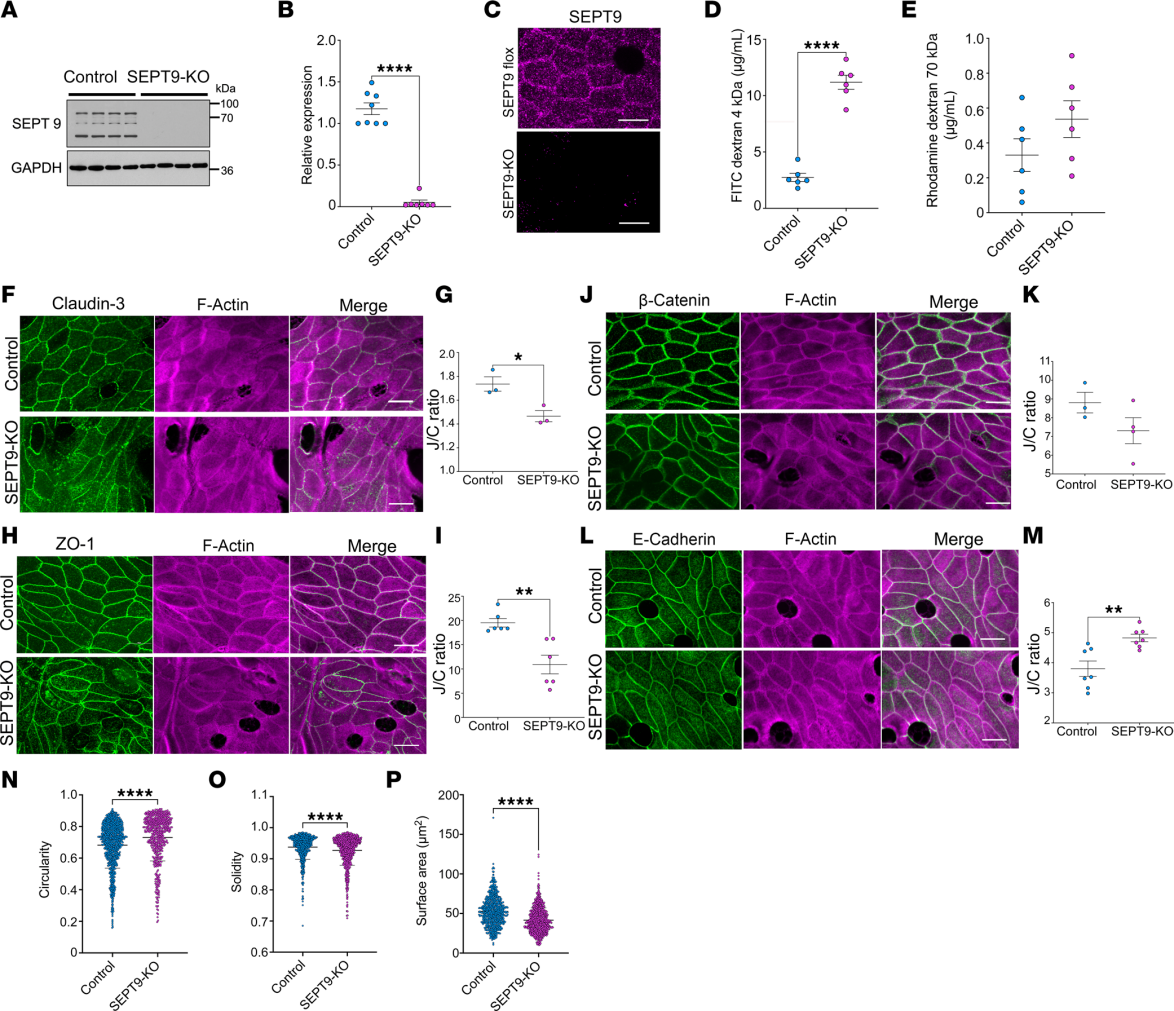
SEPT9 deletion in intestinal epithelium increases permeability and disrupts apical junction organization in vivo. (**A**) Representative immunoblot confirming loss of SEPT9 protein in colonic scrapes from SEPT9–conditional KO (SEPT9-KO) mice compared with control (fl/fl) littermates. GAPDH serves as a loading control (*n* = 4 mice per group). (**B**) Quantification of SEPT9 protein expression normalized to GAPDH from **A** (*n* = 8 mice per group). (**C**) Representative confocal images of SEPT9 immunostaining in colonic epithelium from control and SEPT9-KO mice. Scale bars: 10 μm. (**D** and **E**) Fluorescent tracer assays for intestinal permeability. SEPT9-KO mice show increased serum levels of orally administered FITC-dextran (4 kDa) (**D**), while no significant difference is observed for larger Rhodamine-dextran (70 kDa) (**E**). (**F**, **H**, **J**, and **L**) Confocal images of colon from control and SEPT9-KO mice immunolabeled for tight and adherens junction proteins (claudin-3, ZO-1, β-catenin, and E-cadherin; green), and F-actin (magenta) Scale bars: 5 μm. (**G**, **I**, **K**, and **M**) Quantification of junctional/cytoplasmic fluorescence intensity ratio (J/C ratio) for each marker (*n* = 3–7 mice per group). Statistical analysis was performed using nested ANOVA to account for multiple measurements per animal. (**N**–**P**) Morphometric analysis of IECs shows increased cell circularity (**N**), and decreased solidity (**O**) and apical surface area (**P**) in SEPT9-KO tissue, consistent with altered actomyosin tension. Data are presented as mean ± SEM. *P < 0.05, **P < 0.01, ****P < 0.0001.

Given the observed association of SEPT9 complexes with intestinal epithelial AJs and TJs in vivo, we next sought to investigate a potential role for SEPT9 in regulating gut barrier integrity using SEPT9^fl/fl^Vil1^CreERT2^ mice (referred to hereafter as SEPT9-KO), to enable tamoxifen-inducible IEC-specific KO of SEPT9. SEPT9^fl/fl^ littermates were used as controls for this study. Immunoblotting analysis of isolated IECs demonstrated an almost complete loss of SEPT9 protein after tamoxifen-induced *Cre* recombinase activation (Figure 3, A and B). This loss of SEPT9 was also validated by en face imaging of immunolabeled colonic mucosa of SEPT9-KO animals (Figure 3C).

SEPT9-KO mice did not show growth retardation or symptoms of spontaneous gastrointestinal disorders, such as diarrhea, rectal prolapses, or bleeding (data not shown). Consistently, they did not show gross abnormalities of their ileal and colonic mucosa (Supplemental Figure 3A) but did present with increases in Ki67^+^ proliferative cells (Supplemental Figure 3, B and C) and goblet cells (Supplemental Figure 3, D and E). To determine whether the intestinal epithelial barrier was compromised in SEPT9-KO mice, we used a fluorescent tracer-based gut barrier permeability assay. We observed an approximately 4-fold increase in the transmucosal flux of 4 kDa FITC-dextran in SEPT9-KO mice as compared with the control littermates (Figure 3D), indicating a leaky gut barrier in the absence of SEPT9. Intestinal permeability to a large tracer, 70 kDa Rhodamine-dextran, was not affected, suggesting that SEPT9-KO does not lead to nonspecific loss of IECs (Figure 3E).

We hypothesized that the observed increase in intestinal epithelial permeability can be attributed to dysfunctional IEC junctions, particularly TJs, in SEPT9-KO mice. Therefore, we used immunofluorescence labeling together with machine-learning image analysis (Figure 3, F–M, and Supplemental Figure 4, A and B) to examine the localization of key TJ and AJ proteins in mouse colonic mucosa. We observed mislocalization of TJ proteins claudin-3 and ZO-1 in IEC of SEPT9-KO mice, with decreased junctional enrichment of these proteins and their increased accumulation in small cytoplasmic vesicles (Figure 3, F–I). AJ proteins, β-catenin, and E-cadherin were differentially affected by loss of SEPT9, with β-catenin localization not affected (Figure 3, J and K) and increased junctional accumulation of E-cadherin (Figure 3, L and M). These observations are consistent with previous studies reporting increased internalization of TJ proteins in animal models with defective intestinal epithelial barrier and mucosal biopsies from patients with IBD (36–40). Of note, while their localization was affected, the expression levels of key AJ and TJ proteins were unchanged (Supplemental Figure 4, C and E). Furthermore, loss of SEPT9 did not affect expression of several other septin proteins in the colonic epithelium (Supplemental Figure 4, D and E).

In addition to mislocalization of junctional proteins in vivo, loss of SEPT9 caused noticeable alterations in IEC morphology (Figure 3, N–P). The shape of epithelial cell-cell contacts and the epithelial cell size is regulated by the tension in cell-cell junctions generated by the perijunctional actomyosin belt (41). We thus posited that alterations in the shape and size of SEPT9-depleted IEC cells could be a consequence of dysregulated junctional tension, which has also been shown to directly affect epithelial barrier permeability (16).

### IEC-specific KO of SEPT9 alters the transcription program in intestinal epithelium in vivo.

**Figure 4:**
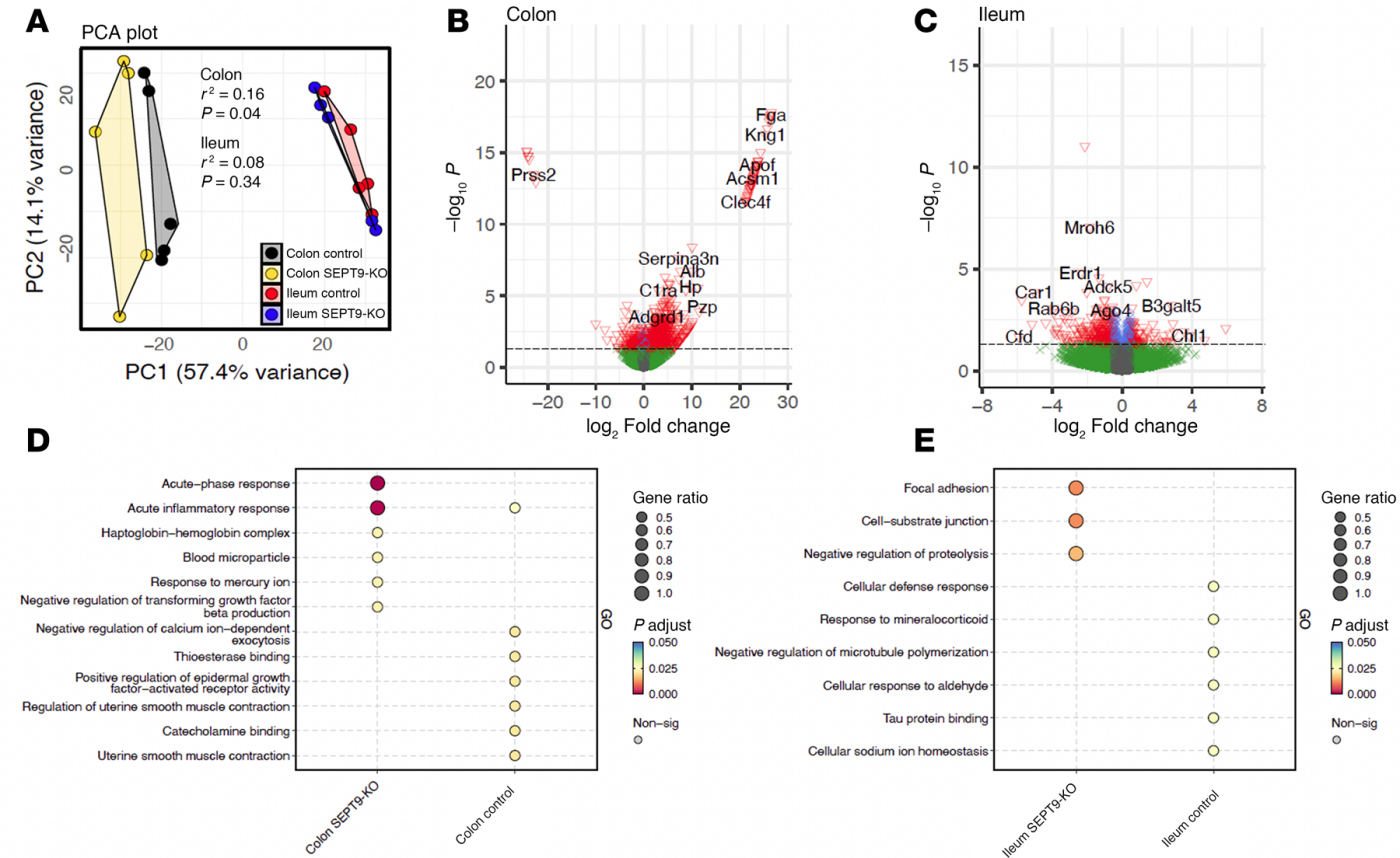
Loss of SEPT9 induces transcription reprogramming of intestinal epithelial cells in mice. (**A**) Principal Component Analysis (PCA) of gene expression profiles in colonic and ileal epithelial cells isolated from SEPT9-KO and control mice. (**B** and **C**) Volcano plots showing gene expression in colonic (**B**) and ileal (**C**) epithelium of SEPT9-KO as compared with control mice. Significant differentially (*P* value and log_2_FC cut-off) expressed genes are highlighted by red inverted triangles. (**D** and **E**) Bubble plots representing pathway enrichment analysis of genes with statistically significant differences in expression between the SEPT9-KO and control mice in the colonic (**D**) and ileal (**E**) epithelium. Each bubble corresponds to a specific pathway, with size indicating the gene ratio and color representing the significance of the enrichment. The analysis was performed using isolated IEC from 5 mice/group.

We next asked if the increased intestinal permeability in SEPT9-KO mice (Figure 3D) was accompanied by alterations in IEC homeostasis, using the cellular transcriptome as a readout. A bulk RNA-Seq (RNA-Seq) analysis was performed on epithelial cells isolated from the colon and ileum of SEPT9-KO mice and their control littermates. Principal component analysis (PCA) (Figure 4A) illustrated the clustering of the samples based on gene expression profiles, highlighting the distinct transcriptional landscapes in response to the SEPT9 KO. Loss of SEPT9 resulted in substantial transcriptional reprogramming of the colonic epithelium (Figure 4A) manifested by upregulation of 123 genes and downregulation of 18 genes in mouse colonic epithelial cells (Figure 4B). In mouse ileal epithelial cells, SEPT9 KO did not trigger global transcriptional changes (Figure 4A) but caused alterations of subset of genes (downregulation of 75 genes and upregulation of 59 genes) (Figure 4C). The list of the top 50 genes differentially expressed in SEPT9 versus control IECs is presented in the Supplemental Table 2. The pathway enrichment analysis revealed upregulation of transcripts related to acute phase response and acute inflammatory response and revealed downregulation of transcripts related to epidermal growth factor receptor signaling in colonic mucosa of SEPT9-KO mice (Figure 4D). In ileal epithelium, loss of SEPT9 increased expression of genes related to focal adhesions and other cell-matrix adhesions and downregulated microtubule-related genes (Figure 4E). These changes likely reflect both permeability-dependent and permeability-independent roles of SEPT9 in epithelial physiology. The observed alterations in microtubule, growth factor receptor, and matrix adhesion pathways are consistent with known septin functions in regulating other cytoskeletal elements and cell interactions with extracellular matrix (17–19).

### IEC-specific KO of SEPT9 exacerbates the severity of acute mucosal inflammation and cell death during experimental colitis.

**Figure 5:**
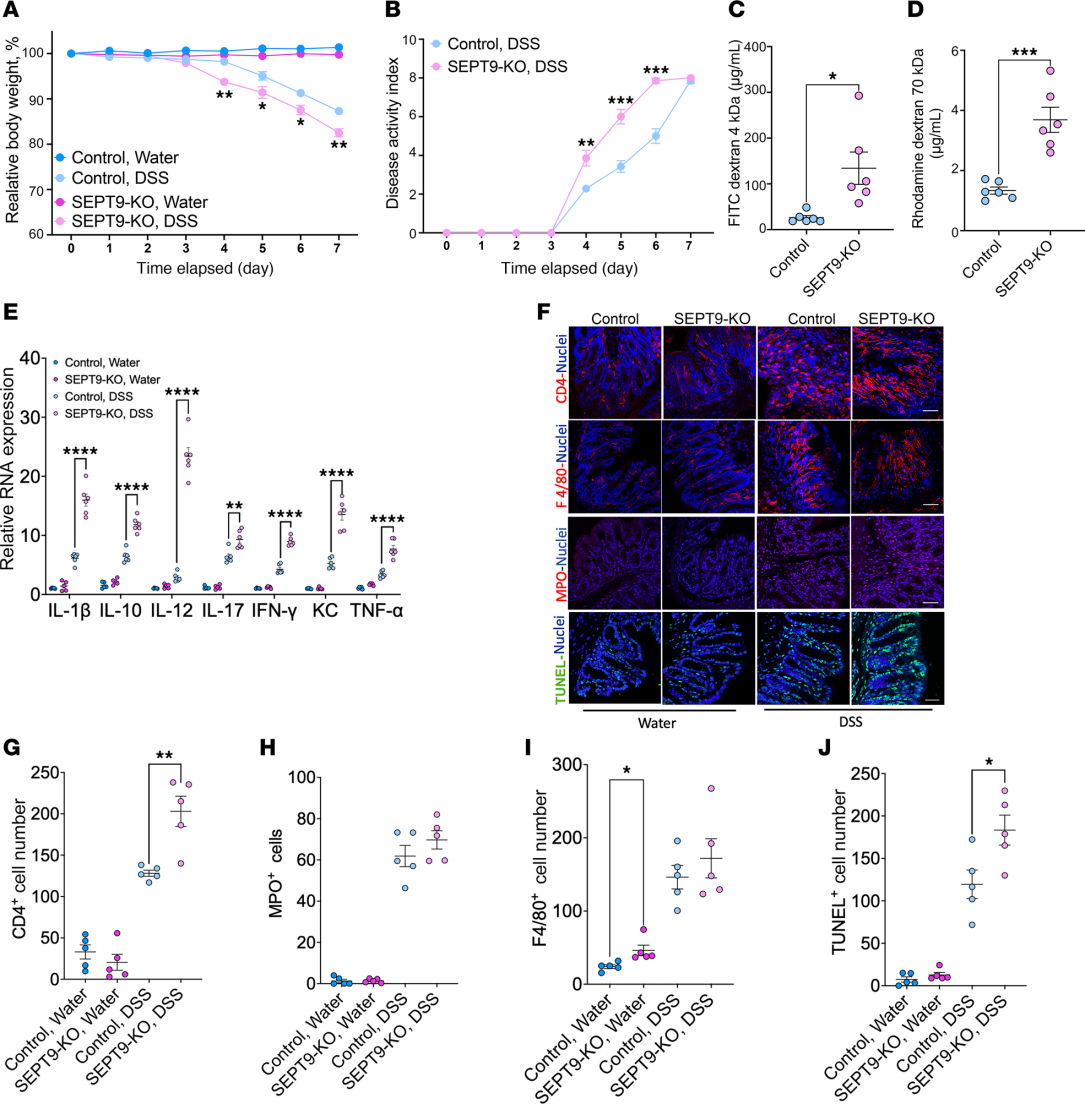
Loss of epithelial SEPT9 exacerbates colitis severity, intestinal barrier dysfunction, and mucosal inflammation. (**A** and **B**) DSS-induced colitis in control and SEPT9-KO mice. SEPT9-KO animals show significantly greater weight loss (**A**) and increased disease activity index (**B**) over 7 days of 3% DSS treatment. (**C** and **D**) Intestinal permeability assays showing increased serum concentrations of orally administered 4 kDa FITC-dextran (**C**) and 70 kDa Rhodamine-dextran (**D**) in DSS-treated SEPT9-KO mice compared with controls (*n* = 5–6 per group). (**E**) qPCR analysis of inflammatory cytokine and chemokine gene expression in colonic tissue following DSS treatment. SEPT9-KO mice exhibit significantly elevated levels of IL-1β, IL-10, IL-12, IL-17, IFN-γ, KC, and TNF-α (*n* = 5–6 mice per group). (**F**) Representative immunofluorescence images of colon sections stained for CD4^+^ T cells, F4/80^+^ macrophages, MPO^+^ neutrophils, and TUNEL^+^ apoptotic cells in water- or DSS-treated groups. Nuclei stained with DAPI (blue). Scale bar: 100 μm. (**G**–**J**) Quantification of immune cell infiltration and cell death in control and DSS-treated colons. Data are presented as mean ± SEM. *P < 0.05, **P < 0.01, ***P < 0.001, ****P < 0.0001 by unpaired 2-tailed *t* test or nested one-way ANOVA with Tukey’s multiple comparisons as appropriate.

Increased permeability of the intestinal epithelial barrier is known to exacerbate inflammatory response in the gut (4, 5, 42). Therefore, we next investigated whether gut barrier disruption observed in SEPT9-KO mice could modulate mucosal inflammation and injury using a dextran sodium sulfate (DSS) model of acute colitis. Exposure to DSS resulted in more severe intestinal disease in SEPT9-KO mice, as compared with their control littermates (Figure 5, A and B). The exaggerated disease was characterized by more pronounced body weight loss (Figure 5A) and a significantly higher disease activity index (Figure 5B), which is a composite of body weight loss, occurrence of diarrhea, and intestinal bleeding. Furthermore, SEPT9-KO mice demonstrated higher intestinal permeability to low and high molecular weight tracers on day 7 of DSS colitis (Figure 5, C and D). The increased permeability to large (70 kDa) molecules could reflect higher mucosal erosion and IEC loss in SEPT9-KO mice. Surprisingly, a cumulative histology score reflecting tissue inflammation and injury and based on the evaluation of H&E-stained sections of distal colon was not significantly elevated in DSS-treated SEPT9-KO mice after 7 days of acute colitis (Supplemental Figure 5, A and B). This is consistent with converged disease activity indexes in 2 experimental groups and may reflect the eventual leveling of the disease symptoms in SEPT9-KO and control mice at late stages of DSS colitis (Figure 5B).

Examining H&E-stained colonic tissue sections provides only a general snapshot of the intestinal architecture. Therefore, we next used a set of specific experimental approaches to evaluate important aspects of mucosal inflammation and tissue injury in DSS-treated animals. Expression of proinflammatory cytokines and chemokines was examined by quantitative PCR (qPCR) analysis in tissue samples of the distal colon collected on day 7 of DSS administration. mRNA expression of several major cytokines (TNF-α, IFN-γ, IL-1β, IL-10, IL-12, IL-17) and 1 keratinocyte-derived cytokine (KC), was significantly higher in tissue samples of DSS-treated SEPT9-KO mice, as compared with their control littermates (Figure 5E). Of note, mRNA levels of these mediators were not elevated in colonic tissue of control SEPT9-KO mice without DSS treatment (Figure 5E). This suggests that the increased permeability of the healthy gut barrier in these animals is insufficient to trigger spontaneous mucosal inflammation.

Since recruitment and activation of immune cells serve as a major driver of mucosal inflammation, we next examined whether loss of SEPT9 affects leukocyte infiltration into inflamed colonic tissue during DSS colitis. Immunofluorescence labeling of CD4, F4/80, and myeloperoxidase (MPO) antigens was utilized to detect T-lymphocytes, monocytes/macrophages, and neutrophils, respectively (Figure 5F). The number of monocytes/macrophages (Figure 5I), but not T cells (Figure 5G) or neutrophils (Figure 5H), was significantly increased in the unchallenged colonic mucosa of SEPT9-KO mice. While DSS colitis induced marked accumulation of all types in immune cells in inflamed colonic mucosa, only T cell numbers were significantly increased in SEPT9-KO mice compared with controls under DSS treatment (Figure 5, F–I). Immune cell infiltration and production of proinflammatory cytokines are known to cause excessive IEC death, thereby exacerbating epithelial barrier disruption and mucosal inflammation (38, 43). A TUNEL assay was used to allow detection of both apoptotic and nonapoptotic cell death (44). Expectedly, DSS administration caused a marked increase in TUNEL^+^ cells, indicating accelerated cell death (Figure 5, F and J). A predominant fraction of these TUNEL^+^ cells localized in the epithelial layer indicating IEC death in DSS-treated animals (Figure 5F). The magnitude of this mucosal cell death was significantly higher in DSS-treated SEPT9-KO mice compared with their controls (Figure 5, F and J). Together, our findings suggest that loss of SEPT9 in the intestinal epithelium markedly increases sensitivity to experimental colitis by propagating the inflammatory response and cell death in the intestinal mucosa.

### SEPT9 loss increases epithelial barrier permeability and alters the assembly of IEC apical junctions in vitro.

**Figure 6:**
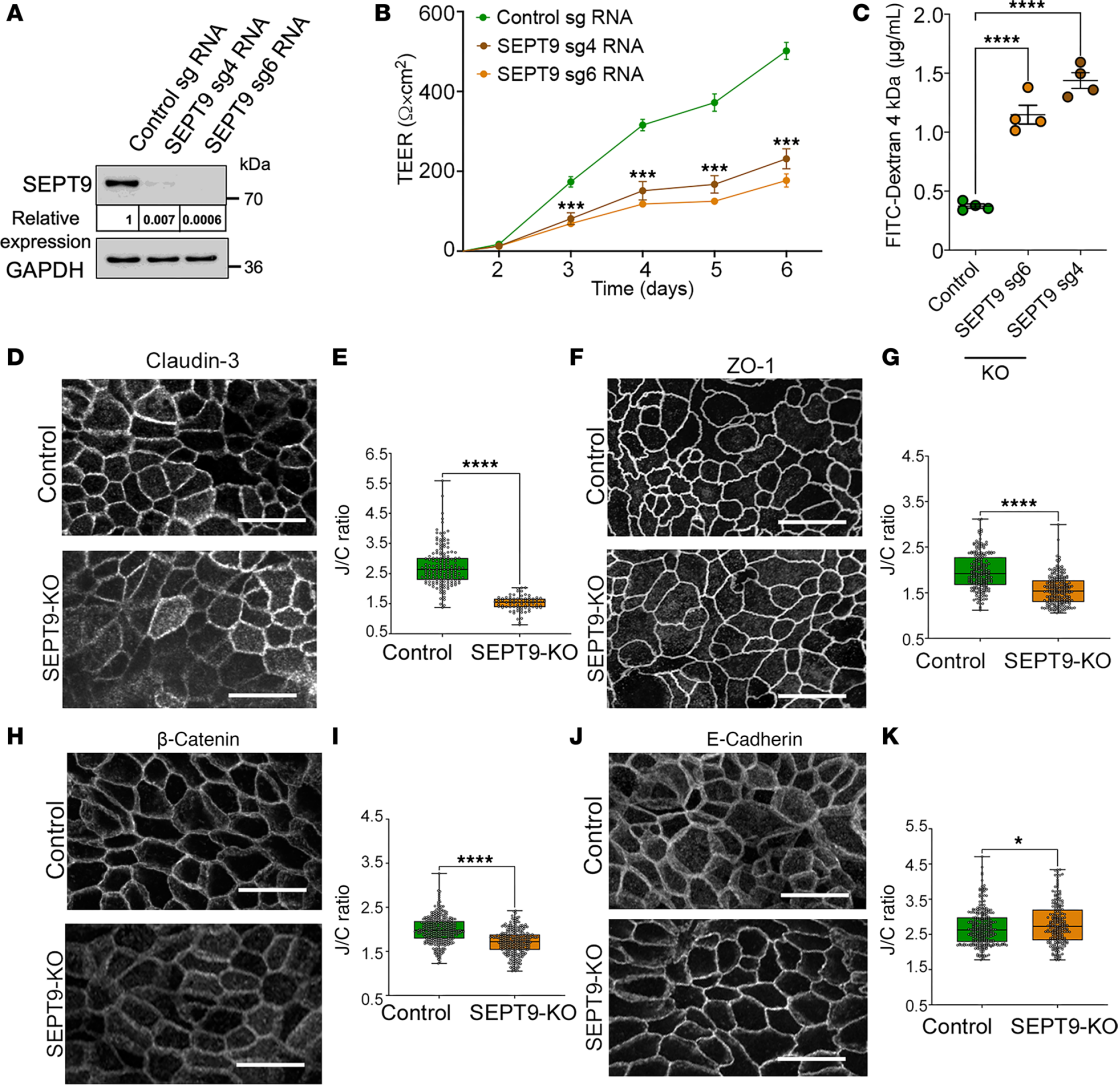
SEPT9 knockdown in model intestinal epithelial monolayers disrupts barrier function and apical junctional organization. (**A**) Immunoblot showing efficient SEPT9 knockdown in HT-29 cF8 cells using 2 independent sgRNAs (sg4 and sg6) compared with nontargeting control sgRNA. GAPDH serves as a loading control. Relative SEPT9 expression is normalized to control and GAPDH. (**B**) Transepithelial electrical resistance (TEER) measurements over 6 days show significantly impaired barrier formation in SEPT9-depleted HT-29 monolayers. (**C**) FITC-dextran (4 kDa) flux assay reveals increased paracellular permeability in SEPT9-KO monolayers (permeability data are representative of 3 independent experiments). (**D**–**K**) Representative confocal images (**D**, **F**, **H**, and **J**) and quantification (**E**, **G**, **I**, and **K**) of junction/cytoplasmic ratio (J/C ratio) of immunolabeled junctional markers in control and SEPT9-KO HT-29 cells, including claudin-3 (**D** and **E**), ZO-1 (**F** and **G**), β-catenin (**H** and **I**), and E-cadherin (**J** and **K**). Scale bars: 10 μm. Data are shown as mean ± SEM from ≥ 3 biological replicates. *P < 0.05, ***P < 0.001, ****P < 0.0001 by unpaired 2-tailed *t* test.

To establish whether the observed disruption of the gut barrier represents an autonomous effect of SEPT9 depletion in IECs and to develop an in vitro model system to probe the underlying molecular mechanisms, we created HT-29 cF8 human colonic epithelial cell lines with CRISPR/Cas9-mediated KO of SEPT9 (Figure 6A). Similar to the results obtained in murine intestinal mucosa, SEPT9-deficient IEC monolayers demonstrated significantly increased paracellular permeability based on TEER (Figure 6B) and FITC-dextran flux (Figure 6C) measurements. Barrier disruption in SEPT9 KO HT-29 cells was accompanied by decreased junctional accumulation of claudin-3, ZO-1, occludin and β-catenin and increased junctional recruitment of E-cadherin (Figure 6, D–K, and Supplemental Figure 6, A and B), without changes in expression levels of different AJ/TJ proteins (Supplemental Figure 6C). While the effects of SEPT9 deletion on apical junctions in model IEC monolayers were largely consistent with the in vivo effects of SEPT9 depletion in the intestinal epithelium, β-catenin expression at junctions was unchanged in SEPT9-KO colonic tissue but decreased in HT-29 cells. We hypothesize that this difference reflects cell type and tissue-context effects; in transformed HT-29 cells, SEPT9 loss may lead to destabilization of the cadherin–β-catenin complex at AJs, whereas in intact colonic epithelium, redundant pathways and tissue-level constraints likely buffer against such changes.

We next sought to assess the dynamics of TJ proteins as a readout of TJ stability. To do this, we expressed GFP-tagged claudin-7 in control and SEPT9-deficient HT-29 cells and performed live cell imaging. Strikingly, we found that, while claudin-7 was primarily localized at apical junctions in control cells, it was translocated into numerous cytoplasmic vesicles after SEPT9 depletion (Supplemental Video 1), supporting a role for SEPT9 in stabilizing IEC junctions. Since these claudin-7 containing vesicles predominantly accumulated in the vicinity of intercellular junctions, their appearance likely indicates either increased endocytosis or decreased recycling of claudin-7 at apical junctions of SEPT9-deficient IECs. In contrast to the increased proliferation observed in SEPT9-KO intestinal epithelia in vivo (Supplemental Figure 3, B and C), SEPT9 KO in HT-29 cells did not affect IEC proliferation (Supplemental Figure 6, D and E), potentially reflecting intrinsic differences between normal IECs and this colon cancer–derived cell line.

### SEPT9 interacts with nonmuscle myosin IIC and regulates junctional localization of this actin motor.

To investigate the mechanisms of SEPT9-dependent regulation of junctional stability and barrier permeability in the intestinal epithelium, we next determined the interactome of SEPT9 in IECs by performing affinity purification mass spectrometry (APMS) on pulldowns of SEPT9-GFP overexpressed in DLD-1 cells. In addition to interactions with several TJ and AJ proteins, analysis of the interactome revealed a strong association of IEC SEPT9 with several major components of the actomyosin cytoskeleton, including nonmuscle myosin II motors, NMIIC, and NMIIB (Supplemental Figure 7, A–C). These findings are consistent with our data showing altered cell shapes in the SEPT9-KO IECs (Figure 3, N–P), which could be due to altered actomyosin tension, as well as with reported interactions between septins and NMII in different mammalian cells (23, 45, 46). We next tested the hypotheses that loss of SEPT9 causes a reduction in NMII-associated forces at IEC apical junctions, leading to impaired TJ assembly. Since NMIIC is highly enriched in the murine intestinal epithelium in vivo (47), while NMIIB is not expressed (38), we focused on investigating the interactions between SEPT9 and NMIIC.

**Figure 7:**
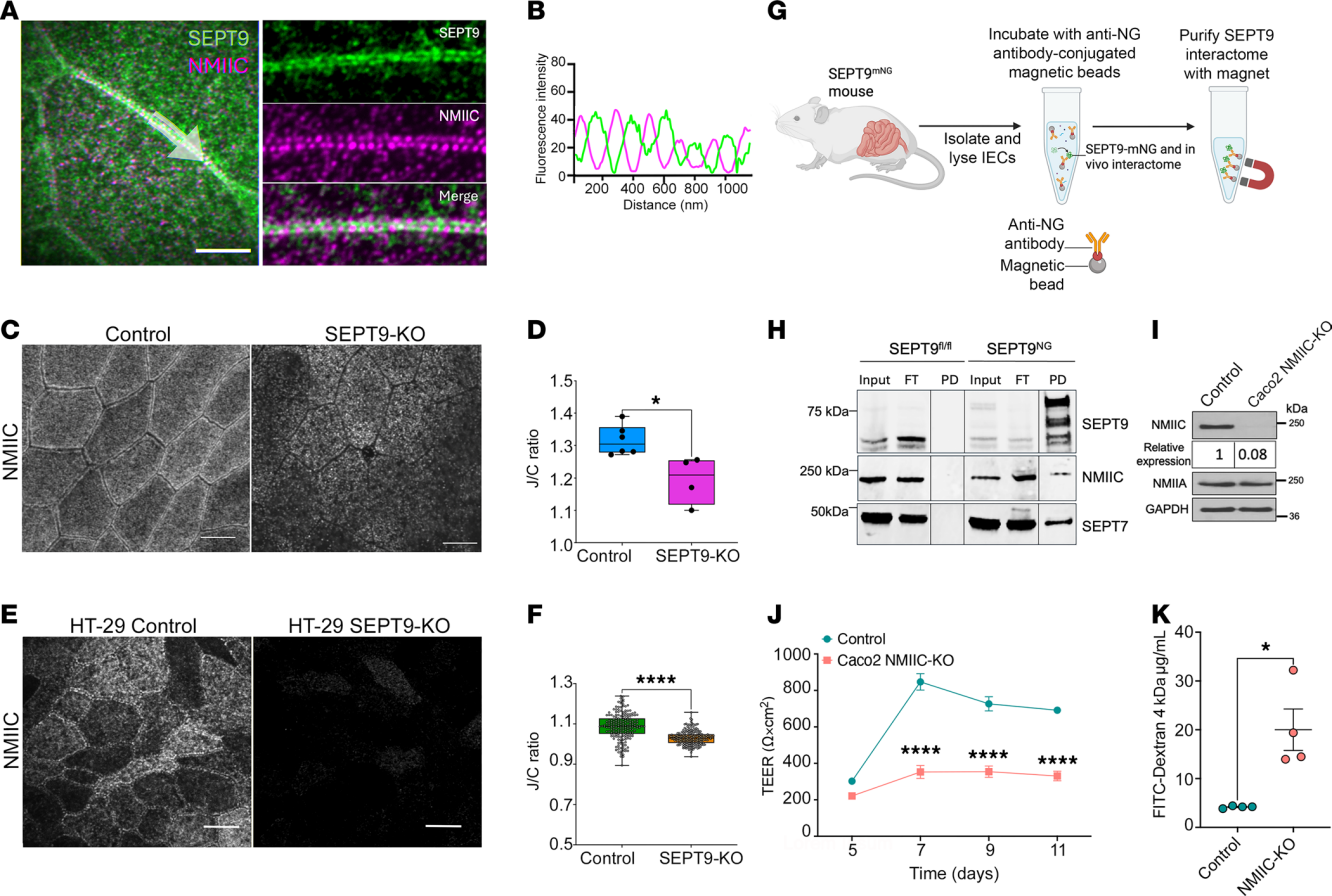
SEPT9 interacts with NMIIC and promotes its junctional enrichment in intestinal epithelial cells. (**A**) Superresolution microscopy image of SEPT9 (green) and NMIIC (magenta) in DLD-1 human colonic epithelial cells. (**B**) Fluorescence intensities profiles show SEPT9 and NMIIC signal intercalation along the cell-cell junction highlighted by the white arrow in **A**. (**C** and **D**) Representative image and junction/cytoplasmic ratio (J/C ratio) of immunolabeled NMIIC in colonic mucosa of SEPT9-KO and control mice. (**E** and **F**) Representative image and J/C ratio of immunolabeled NMIIC in control and SEPT9-KO HT-29 cF8 cells. (**G**) Schematics of in vivo interactome analysis using SEPT9^mNG^ knockin mice. Mouse colonic epithelial cells were isolated, lysed, and incubated with anti-mNeonGreen–conjugated magnetic beads to purify the native SEPT9 interactome. (**H**) Immunoblot analysis of anti-NG antibody pulldowns from SEPT9^fl/fl^ and SEPT9^mNG^ mouse colonic epithelial cells probed with anti- SEPT9, NMIIC and SEPT7 antibodies. Input- total cell lysates; FT-flow through; PD-pull down. (**I**) Western blot confirming specific deletion of NMIIC (but not NMIIA) in CRISPR/Cas9-edited Caco-2BBE epithelial cells. (**J** and **K**)Transepithelial electrical resistance (TEER) and FITC-dextran permeability assays reveal impaired barrier formation in NMIIC-deficient cell monolayers compared with controls. Data are shown as mean ± SEM. *P < 0.05, ****P < 0.0001 by unpaired 2-tailed *t* test.

Superresolution microscopy revealed a precise and periodic pattern of SEPT9 interdigitating with NMIIC sarcomeres at IEC apical junctions (Figure 7, A and B), supporting the hypothesis that these proteins interact. Additionally, when coexpressed in COS7 cells lacking endogenous NMIIC, SEPT9 and NMIIC both showed extensive colocalization along actin-rich stress fibers (Supplemental Figure 7D); these cells were chosen due to their suitability for high-efficiency transfection and high-resolution imaging, allowing us to visualize protein localization and interactions more clearly. As further validation of interaction between SEPT9 and NMIIC in vivo and in vitro, we observed a significant reduction in junctional NMIIC localization in the intestinal epithelium of SEPT9-KO mice (Figure 7, C and D) and SEPT9-deficient HT-29 cell monolayers (Figure 7, E and F), which was not accompanied by the altered total cellular levels of NMIIC and NMIIA (Supplemental Figure 4, C and E and Supplemental Figure 6C). These data highlight the selective association of SEPT9 and NMIIC at IEC junctions. We next sought to test whether SEPT9 and NMIIC interact biochemically. To this end, we pulled down SEPT9-mNeonGreen, and its in vivo interactome, from colonic epithelial cells of the SEPT9^nNG^ mouse using anti-NeonGreen antibody (Figure 7G). We confirmed a successful pulldown using antibodies against SEPT9; as expected, neither of these proteins was pulled down from tissue extracted from SEPT9^fl/fl^ animals (Figure 7H). We then confirmed that NMIIC coimmunoprecipitated with the SEPT9-mNeonGreen, along with a known SEPT9 interactor, SEPT7, which served as a positive control (Figure 7H). These results demonstrate a biochemical interaction between SEPT9 and NMIIC in mouse intestine.

Finally, CRISPR/Cas9 dependent KO of NMIIC in Caco-2BBE cells (Figure 7I) recapitulated the effects of SEPT9 deletion, resulting in epithelial barrier disruption as measured by significantly decreased TEER (Figure 7J) and increased FITC-dextran flux (Figure 7K). Overall, these data suggest that SEPT9 enhances the integrity of the IEC barrier by recruiting/stabilizing NMIIC motor at epithelial TJs.

## Discussion

Increased permeability of the intestinal epithelial barrier is known to exacerbate mucosal inflammation in chronic gastrointestinal disorders, most notably in IBD (1–3). IEC barrier integrity depends on proper assembly of junction-associated cytoskeleton, which is composed of elaborate filamentous networks. In this study, we identify a potentially novel cytoskeletal mechanism regulating permeability of the gut barrier under homeostatic conditions and during mucosal inflammation. This mechanism involves the SEPT9-based cytoskeleton that strengthens the IEC barrier through the recruitment or scaffolding of NMIIC within the apical junctional actomyosin belt, thereby promoting the assembly and stability of TJs and AJs.

SEPT9 is a unique component of the septin oligomers that self-assemble in high-order cytoskeletal structures with various cellular functions (22, 48, 49). Previous studies observed the association of different septins with AJs and reported conflicted effects of septin depletion on AJ protein expression. While loss of SEPT2 decreased VE-cadherin level in endothelial cells (26), SEPT2 and SEPT7 knockdown did not affect E-cadherin and β-catenin expression in cultured Caco-2 cells (25), and SEPT9 depletion decreased E-cadherin and β-catenin levels in MDCK kidney epithelial cells (24). We show for the first time to our knowledge that SEPT9 associates with both AJs and TJs in cultured human IEC and mouse intestinal epithelium and, importantly, that loss of SEPT9 increased intestinal epithelial permeability and impaired TJ assembly and dynamics in vitro and in vivo. In our study, loss of SEPT9 did not affect expression levels of major TJ and AJ proteins in mouse colonic epithelium and human IECs (Supplemental Figure 4, C and E, and Supplemental Figure 6C). This suggests a scaffolding function of SEPT9 in the intestinal epithelium that involves localized recruitment and/or stabilizing proteins at apical junctions.

While SEPT9 may anchor proteins to cell membranes through its phospholipid-binding capability (18), several lines of evidence suggest that it regulates AJ/TJ assembly and function by organizing the perijunctional actomyosin cytoskeleton (Figure 7 and Supplemental Figure 7). First, our interactome and imaging analyses show that SEPT9 associates with NMIIC motor protein. This interaction is consistent with findings in human breast cancer cells (46). Secondly, SEPT9 loss resulted in reduced enrichment of junctional NMIIC in IECs, in vivo and in vitro, coinciding with TJ instability. Finally, NMIIC loss mirrored the barrier-disruptive effects observed with SEPT9 deletion in IECs. NMIIC is highly expressed in differentiated IECs (38, 47) and is specifically enriched at the perijunctional actin belt (14, 50). Importantly, NMIIC was previously implicated in regulation of microvilli growth in cultured IECs (47). However, other epithelial functions of this actin motor remain uncharacterized. Our study provides the first evidence to our knowledge that NMIIC is essential for IEC barrier integrity and is recruited to epithelial TJs through interactions with SEPT9. These findings help to address a long-standing question about the mechanism by which the circumferential actomyosin belt is tethered to epithelial junctions. A recent study proposed that such a mechanism involves interactions between NMIIB and TJ scaffolding proteins, cingulin and paracingulin, in renal epithelial cells (41). However, this mechanism cannot be applied to IECs that do not express NMIIB. Our study reveals an alternative mechanism that mediates the actomyosin tethering and mechanoregulation of epithelial apical junctions that involves the cortical septin cytoskeleton.

Additionally, we observed that SEPT9 loss altered the transcriptional programming of IECs in mice, leading to upregulation of inflammatory pathways in colonic epithelial cells and increase in cell matrix adhesion pathways in ileal epithelial cells (Figure 4, D and E). The increase in the levels of inflammatory related genes in SEPT9-KO colonic epithelial cells could be an indirect consequence of enhanced barrier permeability that increases mucosal translocation of luminal pathogens and triggers proinflammatory signaling. The colonic selectivity of this response is consistent with a much higher abundancy of colonic versus ileal microbiota. The upregulation of matrix adhesion–related genes in SEPT-9 KO ileal epithelial cells could be a compensatory mechanism responding to altered cell-cell adhesion and mechanotransduction. Alternatively, SEPT9 may directly regulate gene expression, as it was reported recently for the SEPT9-mediated myogenic differentiation of immature myoblasts (51).

The increased gut permeability in SEPT9-KO animals was not sufficient to cause spontaneous intestinal inflammatory disorders. These data are consistent with previous studies with junctional adhesion molecule A–KO mice (52) and mice with conditional KO of β-cytoplasmic actin in IEC (53), and they suggest development of compensatory mechanisms restricting activation of the mucosal immune response under conditions of mildly leaky gut. We found, however, that loss of SEPT9 increases severity of the disease symptoms and enhances mucosal injury and inflammation in acute DSS colitis (Figure 5). While the only previous study reported antiinflammatory features of septins during Shigella infection in zebrafish (54), our findings highlight the role of the septin cytoskeleton in preventing mucosal damage, gut barrier disruption, and inflammation in a human IBD-relevant colitis model. These data are in line with previous studies showing exaggerated animal responses to acute colitis in animals deficient in key molecular constituents of the IEC actomyosin cytoskeleton (38, 53). Importantly, our data suggest the dysfunction of the junction-associated septin cytoskeleton in the intestinal epithelium of patients with IBD. This is particularly evident in CD, where SEPT9 is downregulated and displaced from epithelial junctions (Figure 2). It is likely that such dysfunction of the junction-association cytoskeleton contributes to well-documented gut barrier disruption in patients with IBD and could increase severity of the disease.

The present study adds another level of complexity to the mechanisms of epithelial barrier regulation, which involves the coordinated interplay of transmembrane junction proteins, cytoplasmic adaptors, the actomyosin cytoskeleton, and membrane trafficking (55). While we focused on the role of SEPT9 in coupling apical junctions with NMIIC at the perijunctional actomyosin belt, it is likely that SEPT9 intersects with other known regulators of barrier function in healthy gut and during mucosal inflammation. For example, loss of SEPT9 may impair the junctional recruitment of key TJ components, including ZO-1, ZO-2, occludin, claudins, and JAM-A, which are often mislocalized in the intestinal mucosa of patients with IBD and in DSS-challenged mouse models (56). This disruption likely occurs through defective vesicular trafficking of these proteins to epithelial junctions. Consistent with this, we observed altered perijunctional trafficking of claudin-7 in SEPT9-deficient IECs in vitro (Supplemental Video 1). Furthermore, our proteomic analysis demonstrating SEPT9 interactions with cytoplasmic actins and a major actin crosslinker, α-actinin-4, suggests the direct role of SEPT9 in regulating the structure and turnover of junction-associated F-actin bundles. Importantly, our study also reveals the complexity of myosin-dependent regulation of IEC barrier by describing the previously unknown barrier-promoting function of NMIIC (Figure 7, J and K), as well as the SEPT9-dependent junctional recruitment of this cytoskeletal motor (Figure 7, C and E). Previous studies have highlighted the critical involvement of a homologous motor protein, NMIIA, in regulating IEC barrier, junctional integrity, and gut barrier disruption during DSS colitis (38, 50). It would be important to characterize the functional interplay between NMIIA- and NMIIC-dependent cytoskeletal mechanism in regulating normal IEC barrier and barrier disruption in IBD and other inflammatory disorders.

In conclusion, this study reveals a potentially novel mechanism that regulates integrity of the IEC barrier under homeostatic conditions and plays a protective role in the intestinal mucosa during inflammation. The mechanism involves the SEPT9-based cytoskeleton that associates with apical junctions and regulates TJ and AJ assembly and their coupling with the cortical actomyosin belt. Disruption of this junction-associated SEPT9 cytoskeleton, as observed in patients with IBD, likely contributes to gut barrier dysfunction and exacerbated mucosal inflammation, hallmarks of these diseases. These findings highlight the critical role of SEPT9 in maintaining intestinal barrier integrity and suggest that targeting the septin cytoskeleton may offer therapeutic strategies for IBD.

## Methods

### Sex as a biological variable.

Human tissue samples obtained from both male and female patients were analyzed together. The ratio of male and female patients in the patient groups reflects the IBD patient demographic in both specimen collecting sites. For animal experiments, approximately equal numbers of male and female mice were included in each experimental group, but we did not specifically investigate sex-specific differences in their responses.

### Animals.

Mice from C57BL/6J backgrounds were kept in the animal care facilities at the University of Virginia and Lerner Research Institute under controlled temperature and humidity using 12:12-hour light/dark cycles. Animals were supplied with water and food ad libitum. See the Supplemental Methods for the details about generation of tamoxifen-inducible, intestinal epithelium-specific knockout mice (SEPT9-KO) and SEPT9-mNeonGreen reporter mice.

### Human IBD samples.

Deidentified freshly resected full-thickness human intestinal tissue specimens were procured from patients with active (inflamed or strictured) CD, active UC, and non-IBD controls (diverticulosis or healthy margins of colorectal cancer resections). Given that the specimens were derived from intestinal resections, the degree of inflammation in the patients with CD or UC was uniformly moderate to severe. The non-IBD controls had no inflammation. Due to the uniformly high degree of inflammation, no specific inflammation scoring was performed. The procured samples were provided by the human tissue procurement facility of the Lerner Research Institute of Cleveland Clinic through the services of the Biorepository Core and University of Virginia Biorepository and Tissue Research Facility. Patients provided informed consent for the tissue sample collections. The demographic information for different patient groups is provided in Supplemental Table 1.

### DSS colitis.

Experimental colitis was induced in 8- to 10-week-old SEPT9-KO mice and control littermates by administering a 3% (w/v) solution of DSS in drinking water. Unchallenged animals received tap water ad libitum. Animals were weighed daily and monitored for signs of intestinal inflammation. The disease activity index was calculated by averaging numerical scores of body weight loss, stool consistency, and intestinal bleeding. Detailed information about disease activity index, histology score, and measurement of other parameters of mucosal inflammation is provided in the Supplemental Methods. The in vivo intestinal permeability assay was performed in SEPT9-KO and control mice under normal conditions and after DSS colitis by administering different size fluorescent molecular tracers. For details, see the Supplemental Methods.

### Generation of SEPT9- and NMIIC-KO cell lines.

SEPT9-KO HT-29 cF8 and NMIIC-KO Caco-2BBE cell lines were engineered using the CRISPR/Cas9 V2 system. Single-guide RNAs (sgRNA) were designed with a CRISPR design website (http://crispr.mit.edu/), provided by the Feng Zhang Lab (McGovern Institute, Massachusetts Institute of Technology, Boston, Massachusetts, USA). Their sequences are as follows: SEPT9 sg4 5′-ACGGAACGAGAAGGCCCCGG-3′, sg6 5′-AGAGGGTCCACTTCAAACAG-3′, NMIIC sg1 5′-CCTCGGTCACTGCGTACACG-3′, control sg 5′-GACCGGAACGATCTCGCGTA-3′. SEPT9, NMIIC, and control sgRNAs were cloned into a BbsI restriction site of the lentiCRISPR v2 vector (Addgene, catalog 52961) and confirmed by sequencing. Lentiviruses were produced in HEK293T cells cotransfected with packaging plasmids pCD/NL-BH*DDD (Addgene, catalog 17531), and pLTR-G (Addgene, catalog 17532) using a TransIT-293 transfection reagent (Mirus Bio). Stable SEPT9 and NMIIC-depleted IEC cells were generated by transducing HT-29 and Caco-2BBE cells respectively with lentiviruses containing corresponding sgRNAs and subsequent puromycin selection (5 μg/mL) for 7 days. Control IEC were generated via transduction with the control sgRNA containing lentivirus and puromycin selection.

### Transepithelial electrical resistance (TEER) and FITC-dextran permeability measurements in vitro.

IECs were seeded on the 24-well Costar Transwell membrane inserts with 3-micron pore size (Corning Inc., catalog 3415) coated with rat tail collagen I. The TEER was measured using an epithelial ohmmeter (EVOM2, World Precision Instruments). The resistance of cell-free collagen-coated filters was subtracted from each experimental point. *In vitro* FITC-dextran permeability assay was performed at indicated times as previously described (57). IEC monolayers cultured on Transwell filters were apically exposed to 1 mg/ml of FITC-labeled dextran (4 kDa) in HEPES-buffered Hanks’ balanced salt solution (HBSS+). After 120 min of incubation, samples were collected from the lower chamber, and FITC FI was measured using a BioTek Synergy H1 plate reader, at excitation and emission wavelengths 485 nm and 544 nm, respectively. The amount of FITC-dextran translocated across the epithelial cell monolayer was calculated based on a calibration curve plotted with serial dilutions of the FITC-dextran stock.

### Immunofluorescence labeling and microscopy of whole-mount mouse intestine.

For whole-mount tissue staining for *en face* imaging, formalin-fixed whole tissues were rinsed and cut to the desired size. The tissue samples were then permeabilized in 0.5% Triton X-100-PBS solution (PBST) for 45–60 min at room temperature. The tissues were blocked in 10% normal goat serum for 2 hours at room temperature and then incubated with primary antibodies overnight at 4°C. Next, the tissues were labeled with AlexaFluor-conjugated secondary antibodies for 1 hour at room temperature. Confocal microscopy was conducted using a CSU-W1 Yokogawa spinning disk field scanning confocal system. For image acquisition from fixed tissue sections, a sampling frequency of 30–60 nm was achieved at the camera sensor. Image acquisition was controlled with NIS-Elements software (Nikon Instruments).

### Image processing and segmentation.

Machine learning–based morphological analysis of images stained with junctional markers was conducted using a customized U-Net model implemented in PyTorch (58). This model was specifically trained and optimized to segment epithelial cells based on their junctional staining patterns. Following the generation of binary segmentation masks, the resulting data were imported into ImageJ (v1.54f) (59) for quantitative analysis. Individual segmented cells were then inspected, and any cells displaying suboptimal segmentation were manually excluded from further analyses. The remaining high-quality segmentations were used to quantify cell shape descriptors, including cell area, circularity, and solidity, as well as the average FI. To streamline this process, we implemented customized macros that automated the quantification across all segmented images. See the Supplemental Methods for detailed description of image acquisition and analysis for all other immunolabeling and confocal microscopy studies.

### Statistics.

The quantified data from ImageJ was then imported into Python (v3.10) for further processing and statistical analysis. We developed custom Python scripts to gather and process the data efficiently. Subsequently, these processed data were input into GraphPad Prism (v10.4.0) for statistical analysis and visualization. All scripts and code utilized in this study are available upon request. Data are displayed as a mean ± SEM. The statistical significance of the difference between 2 sets of data was evaluated using the 2-tailed unpaired Student’s *t* test when data were distributed normally. Multigroup comparisons were performed using 1-way ANOVA on animal-level averages. When analyzing immunolabeling of human and animal tissues, nested ANOVA was used to account for clustering within animals.

### Study approval.

All animal studies were reviewed and approved by the IACUC of the UVA School of Medicine and the Lerner Research Institute of Cleveland Clinic. Both male and female mice were used, and all mice were maintained in accordance with guidelines of the UVA School of Medicine and the Lerner Research Institute with regard to the care, welfare, and treatment of laboratory animals. All experiments met or exceeded the standards of the Association for the Assessment and Accreditation of Laboratory Animal Care, International (AALAC), the US Department of Health and Human Services, and all local and federal animal welfare laws. Human tissue collection was approved by Cleveland Clinic IRB under minimal risk IRB approval no. 17-1167. The human tissue harvested at the University of Virginia was approved by the University of Virginia’s Institutional Biosafety Committee (IBC) (protocol no. 9987-22).

### Data availability.

All supporting data values associated with the main manuscript and supplement material, including values for all data points shown in graphs and values behind any reported means, are provided in the Supporting Data Values file. The raw and processed bulk RNA-Seq data generated and reported in this work are available in the NCBI’s Gene Expression Omnibus (GEO) database (Submission ID: SUB14911003; BioProject ID: PRJNA1195935). The mass spectrometry proteomics data have been deposited to the ProteomeXchange Consortium via the PRIDE (60) partner repository with the dataset identifier PXD059782.

## Author contributions

AII and SE conceptualized the study, designed the experiments, interpreted the data, wrote the manuscript, and supervised the study. NGN and GH performed most of the experiments, analyzed and interpreted the data, and contributed to the writing of the manuscript. KM performed cell transfections and pulldowns for mass spectrometry analysis. DR developed machine-learning algorithms and performed image analysis and quantification. YO performed Western blot experiments. EJ, SB, and GS performed mass spectrometry and analysis. AZ performed leukocyte infiltration immunolabeling and quantification. SL performed human tissue immunolabeling and quantification. NS performed RNA-Seq analysis of mouse IECs. RM performed bioinformatic analysis of septin expression using a human single-cell RNA-Seq dataset. LS generated and characterized SEPT9-KO human IECs. AMM performed Western blot experiments and densitometric data analysis. FR supervised patient sample selection and analysis. All authors reviewed the manuscript.

## Funding support

This work is the result of NIH funding, in whole or in part, and is subject to the NIH Public Access Policy. Through acceptance of this federal funding, the NIH has been given a right to make the work publicly available in PubMed Central.

- National Institutes of Health (NIH) National Institute of Diabetes and Digestive and Kidney Diseases (NIDDK) grants R01 DK132038 to AII and FR and P30 DK097948 to FR.
- Owens Family Foundation and the Center for Cell and Membrane Physiology, School of Medicine, University of Virginia through a start-up grants to SE.

## Supplementary Material

Supplemental data

Unedited blot and gel images

Supplemental video 1

Supporting data values

## Version 1 10/07/2025

In-Press Preview

## Version 2 11/24/2025

Electronic publication
