# Supplementary material for: The septin cytoskeleton is a regulator of intestinal epithelial barrier integrity and mucosal inflammation
Source: JCI Insight. 2025 Oct 7;10(22):e191538. doi: 10.1172/jci.insight.191538 (PMC12643519; doi:10.1172/jci.insight.191538)
Supplement: Supplemental data [file jciinsight-10-191538-s123.pdf]

Supplementary Figure 1

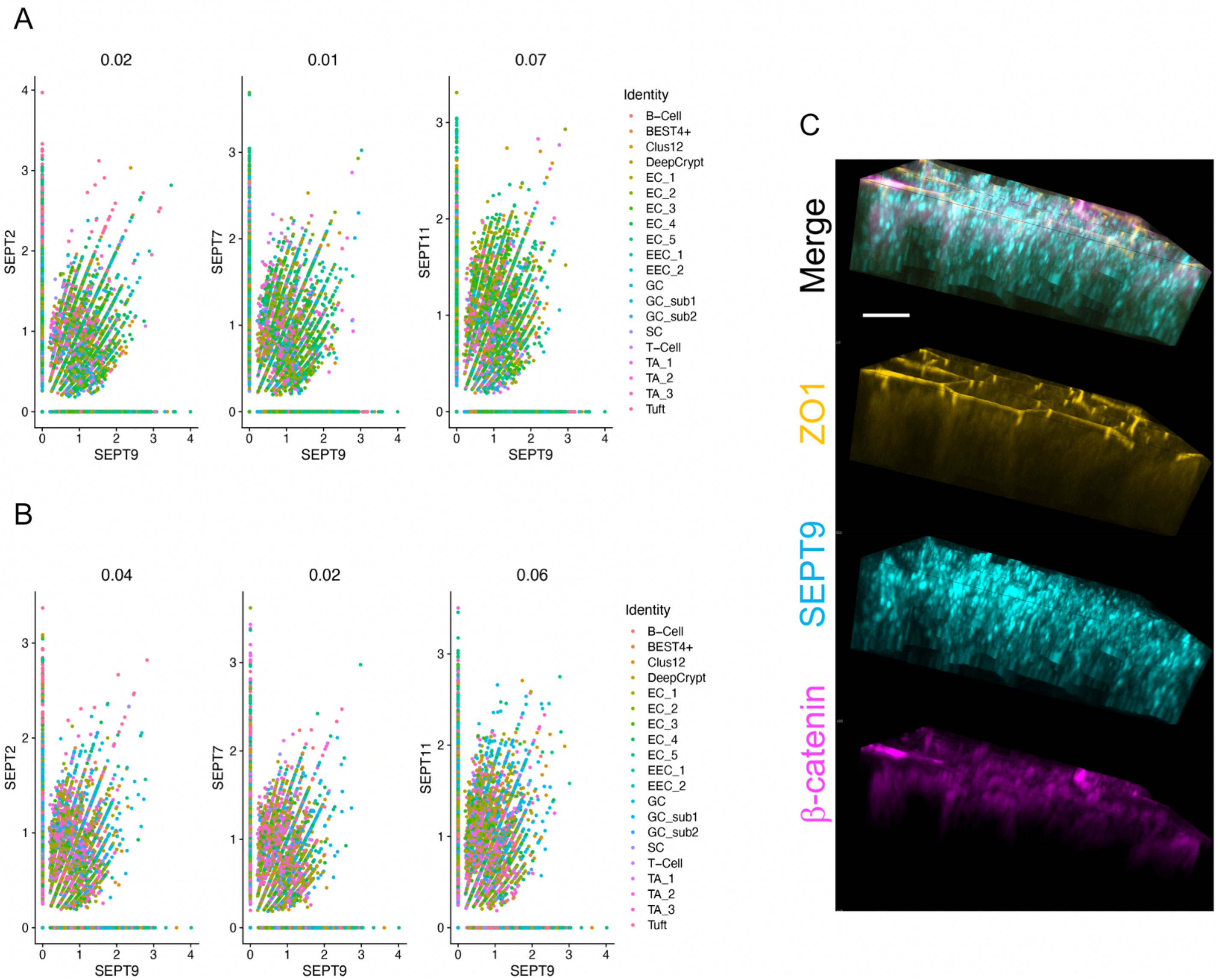

Supplementary Figure 1. SEPT9 correlates with expression of other septins and localizes to apical junctions in the intestinal epithelium. The plots show correlation of SEPT9 transcript with the levels of SEPT2, SEPT7 and SEPT11 transcripts in epithelial cell subpopulation in normal human ileum (**A**) and normal human colon (**B**). Numbers of the top of the graphs are Pearson correlation between two genes. Cell cluster abbreviations are: DeepCrypt, deep crypt secretor; EC, enterocytes; EEC, enteroendocrine; GC, Goblet; SC, stem; TA, transit amplifying; Tuft, tuft cells. (**C**) 3D confocal reconstruction of the apical surface of ileal epithelium from a SEPT9-NG mouse, showing SEPT9-NG (cyan), overlaps with both immune-stained ZO-1 (yellow), and β-catenin (magenta). Scale bar: 10 µm.

Supplementary Figure 2

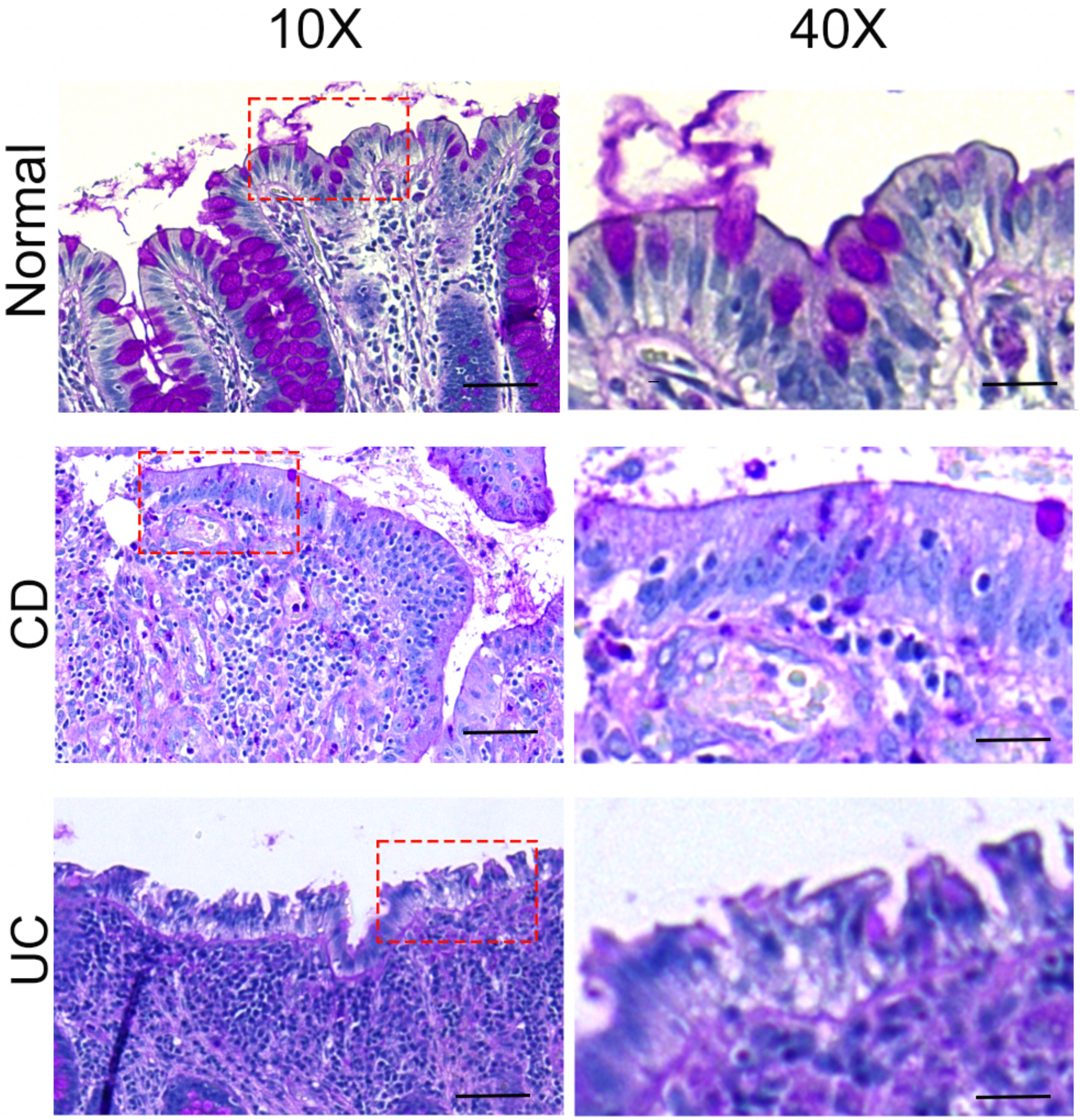

Supplementary Figure 2: PAS staining confirms epithelial integrity in normal and IBD colonic tissues. Representative periodic acid–Schiff (PAS)-stained colonic sections from healthy individuals (Normal) and patients with Crohn's disease (CD) or ulcerative colitis (UC). Images shown at 10X and 40X magnification. Intact epithelial layers with distinguishable apical brush borders and preserved crypt architecture are evident across all samples, supporting the use of these tissues for analysis of epithelial SEPT9 expression. Red dashed boxes in 10X panels indicate areas shown at higher magnification in 40X panels. Scale bars: 50 µm (10X), 10 µm (40X)

Supplementary Figure 3

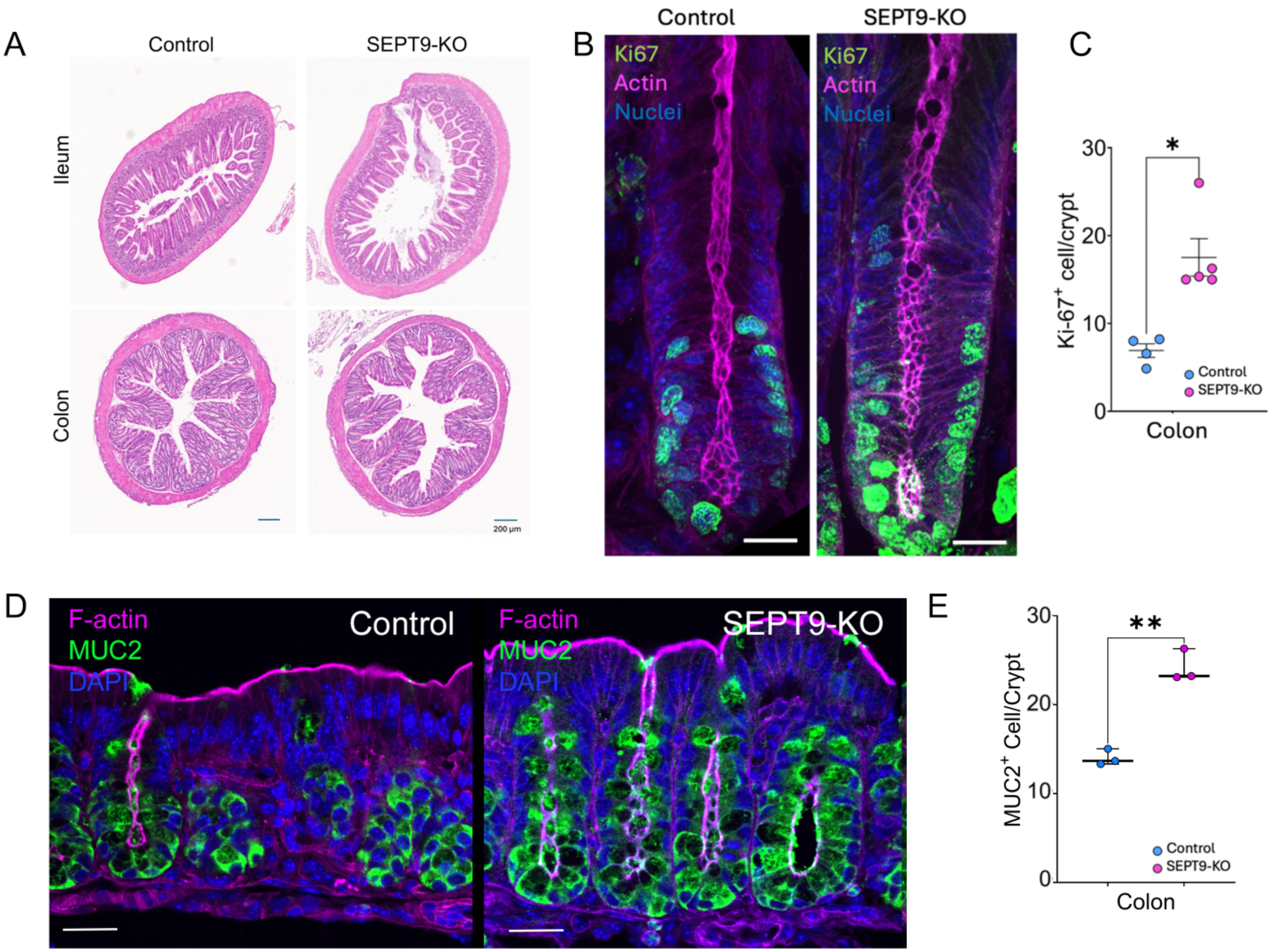

Supplementary Figure 3: Effects of SEPT9 loss on the intestinal architecture, epithelial proliferation, and goblet cell numbers. (**A**) Representative H&E-stained sections of ileum and colon from control and SEPT9 conditional knockout (SEPT9-KO) mice. (**B**) Immunofluorescence images of colonic crypts labeled for Ki-67 (green, proliferating cells), F-actin (magenta), and nuclei (blue) in control and SEPT9-KO mice. Scale bar = 10µm. (**C**) Quantification of Ki-67^{+} cell numbers per crypt (n= 4-5 mice/group). Student's t-test. *p<0.05. (**D**) Immunolabeling of colonic sections for MUC2 (green, goblet cells), F-actin (magenta), and nuclei (blue). (**E**) Quantification of goblet cell numbers per crypt (n= 3 mice/group). Student's t-test. **p<0.01. Scale bars: 200 µm (A), 20 µm (B, D).

Supplementary Figure 4

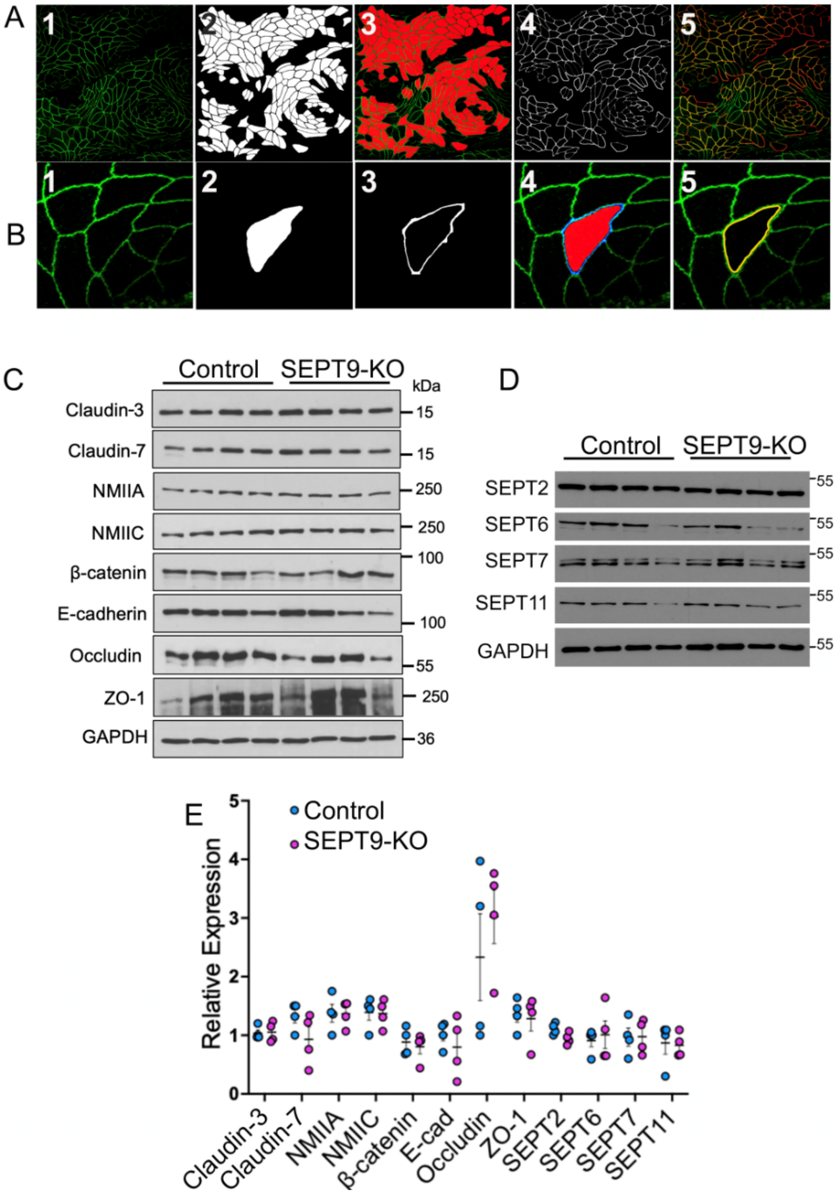

Supplementary Figure 4: Machine-learning segmentation of colonic epithelium and expression of junctional and cytoskeletal proteins in colonic mucosa of control and SEPT9-KO mice. (A and B) Representative images of *en face* intestinal epithelial segmentation in entire and detailed images. (A1) Representative image of junctional protein quantification. 0.6µm of wholemount colon tissue labeled with ZO-1 antibody was employed as an example (A2) Binary mask of cells generated from the original image with additional manual filtration. (A3) Overlay of the original image (green) with the binary mask (red). (A4) Binary mask of the junctions highlighting the intercellular borders. (A5) Overlay of the original image (green) with the junctional mask (red) in yellow. (B1) Representative image of junctional protein quantification. 0.6µm of wholemount colon tissue labeled with ZO-1 antibody was employed as an example (B2) Binary mask of the cell created from the original image. (B3) Binary mask of the junction of the cell of interest. (B4) Overlay of the original image (green) with the binary mask of the cell (red) and the binary mask of the junction (blue). (B5) Marked area (yellow ring) where junctional FI was measured using ImageJ. (**C and D**) Immunoblotting analysis of junctional proteins, myosins (**C**) and septins (**D**) expression in colonic scrapes obtained from control and SEPT9-KO mice. **(E)** densitometric quantification of protein expression (n= 3-4/group). The intensities of the bands for each sample were normalized to those for GAPDH, and the intensity of the bands in the control group was assigned a value of 1

Supplementary Figure 5

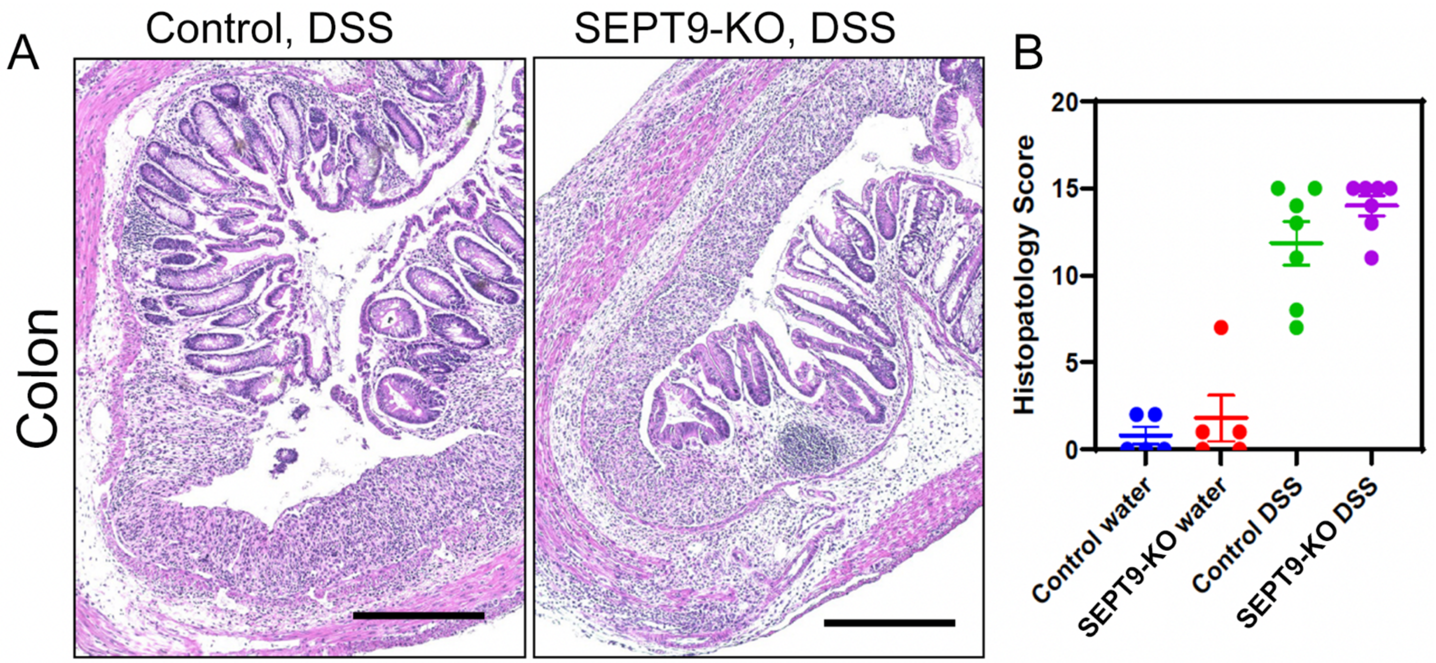

Supplementary Figure 5: DSS-induced colonic injury and inflammation in control and SEPT9 knockout mice. (A) Representative H&E-stained colon sections from DSS-treated control and SEPT9-KO mice. Scale bars: 200 µm. **(B)** Histopathological scoring of colonic tissue across conditions (mean ± SEM; n=5–7 mice per group).

Supplementary Figure 6

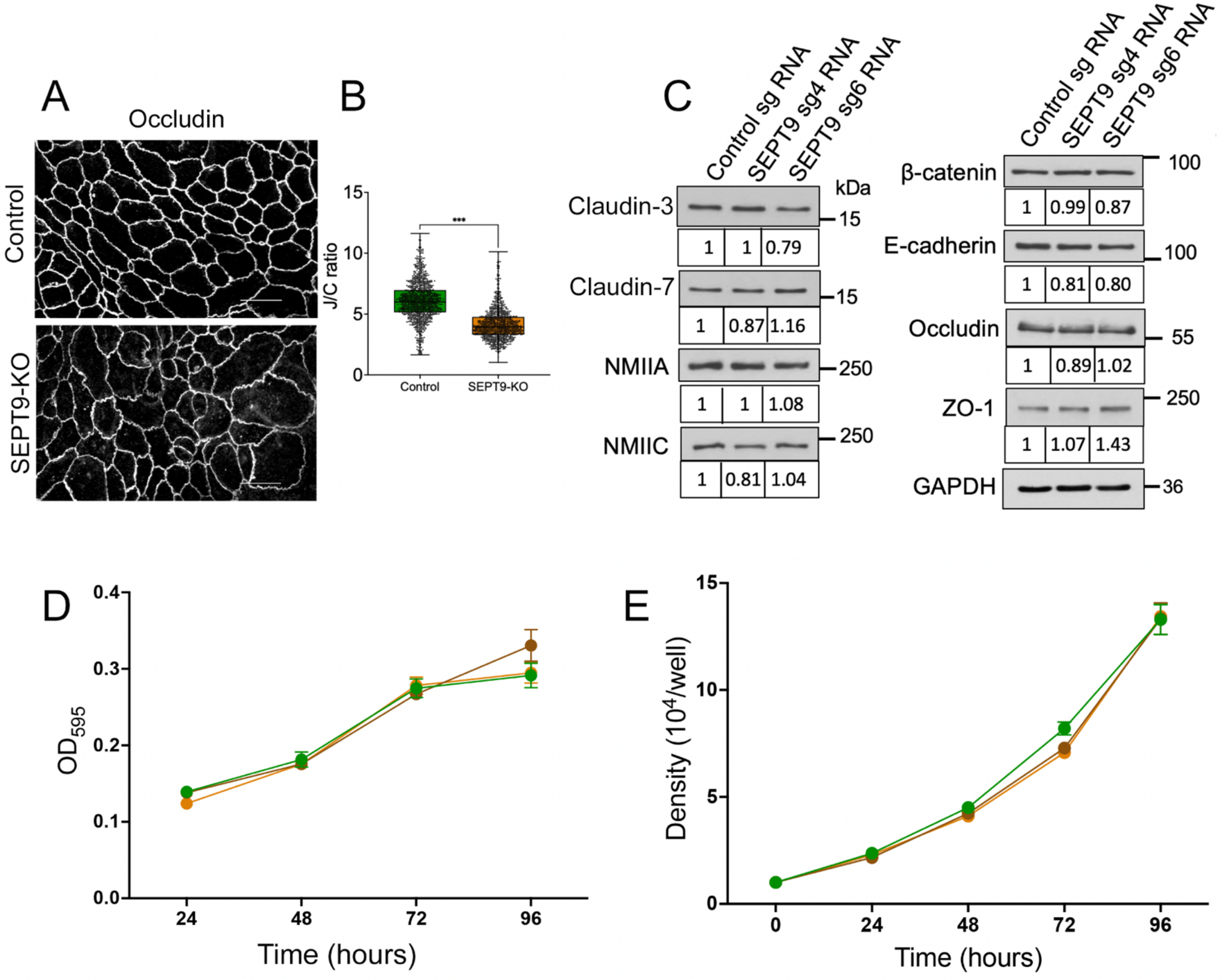

Supplementary Figure 6: SEPT9 depletion in human IECs decreases junctional recruitment of occludin but does not alter steady-state expression of major junctional or cytoskeletal proteins and does not affect epithelial cell proliferation. (A and B) Representative confocal images (**A**) and quantification of junction-to-cytoplasmic ratio (J/C Ratio) (**B**) of immunolabeled occludin in control and SEPT9-KO HT-29 cells. (**C**) Immunoblots of HT-29 cF8 lysates following CRISPR-mediated knockdown of SEPT9 using two independent sgRNAs (sg4 and sg6) compared to non-targeting control. Expression levels of tight junction proteins (claudin-3, claudin-7, occludin, ZO-1), adherens junction proteins (E-cadherin, β-catenin), and myosin motors (NMIIA, NMIIC) are shown. GAPDH serves as a loading control. Densitometric values represent signal intensity normalized to control and GAPDH, averaged across biological replicates. **(D)** MTT assay showing similar metabolic/proliferation activity over time in control and SEPT9-depleted HT-29 cells. **(E)** Cell counting over 96 hours of control and SEPT9-depleted HT-29 cells shows that SEPT9 knockdown does not impair epithelial cell proliferation. Data are mean ± SEM from triplicate wells.

Supplementary Figure 7

A

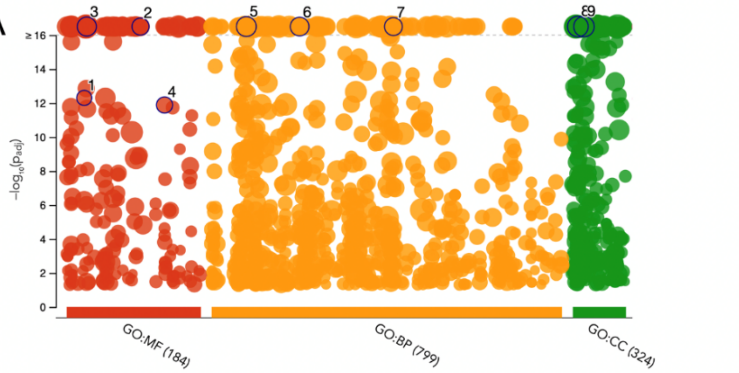

ID	Source	Term ID	Term Name	p_{adj} (query_1)
1	GO:MF	GO:0005200	structural constituent of cytoskeleton	4.995×10^{-13}
2	GO:MF	GO:0045296	cadherin binding	3.145×10^{-89}
3	GO:MF	GO:0008092	cytoskeletal protein binding	7.441×10^{-30}
4	GO:MF	GO:0051015	actin filament binding	1.305×10^{-12}
5	GO:BP	GO:0007010	cytoskeleton organization	7.522×10^{-42}
6	GO:BP	GO:0030029	actin filament-based process	3.954×10^{-23}
7	GO:BP	GO:0051493	regulation of cytoskeleton organization	1.874×10^{-23}
8	GO:CC	GO:0005856	cytoskeleton	5.831×10^{-48}
9	GO:CC	GO:0030054	cell junction	2.838×10^{-40}

C

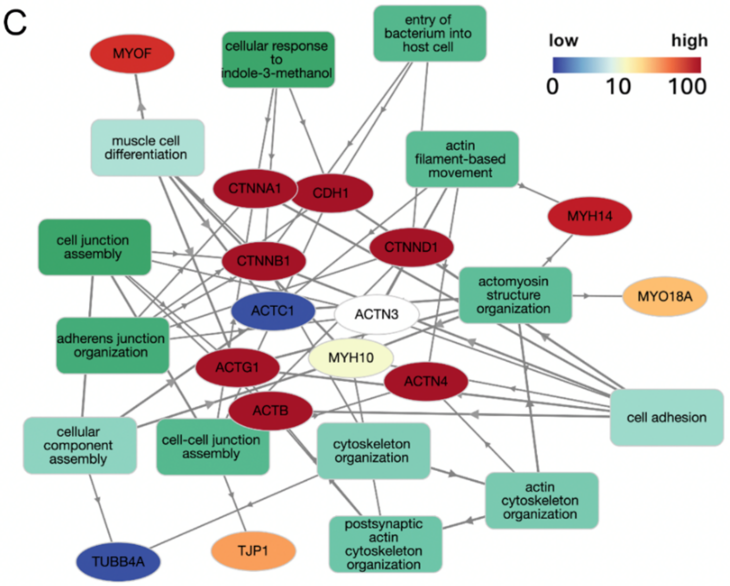

B

Protein (Gene name)	#PSMs (control)	#PSMs (SEPT9)
MYH14	NA	12
LMO7	NA	12
MYH10	NA	11
LUZP1	NA	7
CFAP36	NA	7
ACTN3	NA	6
MYO18A	NA	2
MYO5C	NA	2
ACTB	129	297
ACTG1	128	294
ACTC1	43	113
ACTN4	15	38
TJP1	8	24
MYOF	30	42
MYO1D	3	19
CDH1	5	12
CTNNA1	20	45
CTNND1	15	36
CTNNB1	10	28
TUBB4A	52	368

D

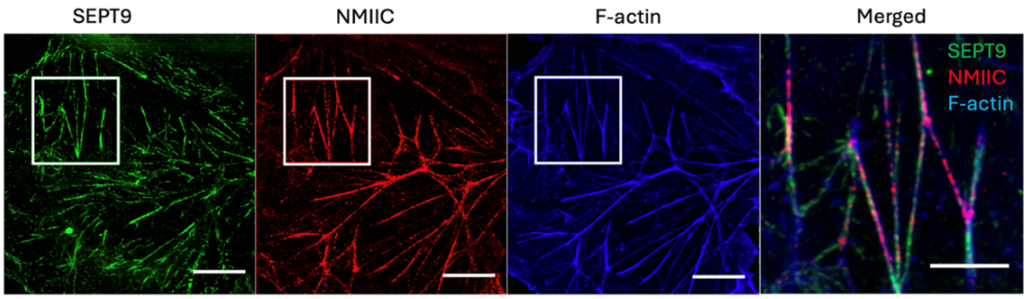

Supplementary Figure 7: Interactome of SEPT9 and co-assembly of SEPT9 and NMIIC within F-actin bundles. (**A**) Gene Ontology (GO) enrichment analysis of proteins enriched in SEPT9 immunoprecipitates compared to control. GO terms were grouped by category: Molecular Function (MF, red), Biological Process (BP, orange), and Cellular Component (CC, green). Based on SEPT9 localization at apical junctions, we focused on cytoskeletal and junctional-associated GO terms, which are listed in table below. **(B)** Table of cytoskeletal and junctional proteins detected in control versus SEPT9 IP samples. (**C**) Functional network of identified SEPT9-associated proteins. Nodes represent individual proteins (ovals) or enriched GO terms (rectangles). Node color corresponds to normalized spectral counts (as indicated by color scale); arrows indicate directional interactions or pathway relevance. (**D**) Confocal microscopy images of COS-7 cells over-expressing SEPT9-GFP (green), NMIIC-mCherry (red), and stained with phalloidin to label F-actin (blue). White boxes indicate regions shown at higher magnification in merged panels. SEPT9 and NMIIC colocalize along actin stress fibers. Scale bars: 20 µm (full image), 5 µm (zoom-in).

Supplementary Table 1

Table 1. Patient information

Cleveland Clinic Patient Cohort				
**Groups**	**Sample number (*n*)**	**Gut segment**	**Age, years, Mean (range)**	**Male sex, n (%)**
Crohn's Disease	8	Colon (5) and ileum (3)	54 (26-70)	2 (25)
Ulcerative colitis	8	Colon	40 (24-57)	6 (75)
Non-IBD control	7	Colon (6) and ileum (1)	64 (14-82)	4 (57)
**University of Virginia Patient Cohort**				
**Groups**	**Sample number (*n*)**	**Gut segment**	**Age, years, Mean (range)**	**Male sex, n (%)**
Crohn's Disease	5	Colon	33 (26-45)	3 (60)
Ulcerative colitis	6	Colon	54 (12-83)	0 (0)
Non-IBD control	9	Colon	59 (44-72)	3 (33)

Supplementary Table 2

Table 2: Bulk RNASeq results of differentially expressed genes in SEPT9-KO versus control colon and ileum epithelium

Gene	log2FoldChange	p	Gene	log2FoldChange	p	Gene	log2FoldChange	p	Gene	log2FoldChange	p
**Downregulated in SEPT9-KO**						**Upregulated in SEPT9-KO**					
**Colon**			**Ileum**			**Colon**			**Ileum**		
Pnlip	-24.75093425	2.68E-06	Cela3b	-27.05364178	1.73E-10	Fga	26.58606699	3.00E-07	Try4	26.15890616	9.82E-10
Cpb1	-24.64210408	2.90E-06	Prss3b	-24.6371633	8.57E-09	Mup3	26.51476016	3.09E-07	Try5	18.04148212	2.49E-05
Prss3b	-23.82252536	7.88E-06	Cpa1	-21.97523572	2.82E-07	Serpina3k	26.47502481	3.09E-07	Fgb	16.96574573	6.00E-05
Cpa1	-23.55771671	1.02E-05	Cfd	-20.75542039	6.52E-08	Gc	26.39674968	3.21E-07	Aox3	16.50799862	0.000104
Cela3b	-23.24422986	1.41E-05	Svs5	-18.71375926	8.56E-06	Fgb	26.36965332	3.21E-07	Cyp1a2	16.42228556	0.000115
Try5	-22.387409	3.25E-05	Amdhd1	-16.89670507	3.56E-07	Fgg	26.02324176	4.89E-07	Fga	15.1881094	0.000387
Prss2	-22.13915231	4.25E-05	Semg1	-16.88287758	7.29E-05	Mug1	25.84561588	6.09E-07	Itih3	14.80656175	0.000541
Try4	-21.80014904	6.16E-05	C8a	-16.6551601	9.18E-05	Kng1	25.14393094	1.58E-06	Apon	14.72042787	0.000233
Mir1938	-3.929909503	0.048611422	Slc10a1	-16.21287094	0.000146763	Cyp2c50	24.72431361	2.68E-06	Serpina6	14.45919389	0.000729
Rnase1	-3.6775699	0.000447864	Slco1b2	-16.06474892	5.25E-09	Serpina1c	24.70872998	2.71E-24	Chl1	3.936913331	0.004619
Snora57	-3.611680069	0.017820835	Cel	-15.92880158	1.30E-06	Serpina1d	24.49611033	7.59E-23	Krt14	3.795880891	0.003295
Gm43697	-3.538886764	0.000815988	Ahsg	-15.88730071	0.0002005	Serpina1a	24.3138309	1.49E-21	mt-Co3	3.537090256	0.009466
mt-Rnr2	-2.996302407	0.044706577	Apoc1	-15.83824356	1.69E-10	Sult2a8	24.12021475	5.68E-06	Gm4518	3.313663888	0.004328
Mal	-2.137129876	0.00837475	Serpind1	-15.46713678	0.000301413	Slc27a5	24.06879177	2.36E-16	Fam184b	3.140826156	0.005767
Fgr	-2.125550347	0.010473716	Orm1	-15.11135253	0.000414072	Mug2	24.06532472	5.92E-06	Scara5	2.993558971	0.009809
Sypl2	-1.975537608	0.000871387	Inmt	-14.59340695	2.64E-05	Apof	23.80080849	7.88E-06	Chi3l1	2.965948331	0.003444
Sis	-1.450910127	0.006661549	Apof	-14.43939473	0.000741011	C8a	23.78974305	7.88E-06	Ak4	2.939243894	0.006005
Dab1	-1.38928102	0.006661549	Car3	-5.744448561	0.00095315	Mup20	23.76843849	7.88E-06	Foxd2os	2.926878228	0.007265
			Fabp4	-5.157791815	0.007861761	Ugt3a1	23.49999703	1.08E-05	B3galt5	2.790114389	1.51E-05
			Car1	-5.077182756	2.57E-06	Cyp2c54	23.47746811	1.08E-05	Fosl1	2.745337184	0.002216
			Mir7675	-3.929082724	0.00200077	Cyp2j5	23.39970479	3.56E-18	Gm37648	2.639397924	0.003394
			Rab6b	-3.884426089	3.21E-05	Cfhr2	23.38975023	2.85E-21	Scd2	1.851188231	0.003538
			Plin4	-3.855912508	0.00339182	Uroc1	23.29133907	1.15E-06	Mamld1	1.765318292	0.003918
			Ttn	-3.793026025	0.001438644	C8b	23.21886411	8.82E-08	Gm43305	1.745086788	0.000879
			Gm13199	-3.706535246	0.003443078	Slc10a1	23.19611944	1.47E-05	Exd1	1.472595556	0.009136
			Mir6240	-3.617187734	0.000937772	Cyp2c37	23.03143899	1.79E-05	Hk2	1.398114695	2.79E-05
			Gm47021	-3.533723307	0.000158439	Slco1a1	22.91118028	2.05E-05	Fut2	1.219218774	0.001549
			Gm50289	-3.50576865	0.009150166	Apcs	22.86425525	2.14E-05	Lrp8	1.211157433	0.008985
			Mir7651	-3.311039376	0.001666631	Aox3	22.77173274	2.36E-05	Gm46620	1.162857202	0.00747
			Necab2	-3.304011457	0.00354054	Acsm1	22.67485901	2.57E-05	Nol6	1.160517169	0.00708
			Zfp114	-3.247413375	0.009782597	Serpina3m	22.6574056	2.57E-05	Tm4sf20	1.009377629	0.009393
			A930004D18Rik	-3.227537839	0.00686643	Gm29966	22.60723597	2.64E-05	Pcdhgc4	0.989431809	0.006504
			Prkag3	-2.864343554	0.008223124	Cyp7a1	22.6062906	2.64E-05	Afap1	0.946471418	0.005028
			Gm47232	-2.661374787	0.005022345	Slc6a13	22.5370879	2.83E-05	Ppfibp1	0.801131342	6.03E-06
			Gm6119	-2.550110259	0.009525292	Svs5	22.51146692	2.87E-05	Ppargc1b	0.700920248	0.002209
			Erdr1x	-2.546316526	5.99E-05	Serpina6	22.42915388	3.14E-05	Ubiad1	0.668307762	0.002458
			Dcdc2b	-2.426382395	0.001974087	Svs4	22.35858841	3.32E-05	Lamc2	0.662612558	0.00571
			Pip5kl1	-2.322643865	0.009881924	Adh4	22.32521577	8.72E-27	Atp10b	0.660320939	0.007947
			Erdr1y	-2.26729425	0.002763425	F13b	22.29090518	2.08E-14	Fndc3b	0.612723316	0.002022
			4930481A15Rik	-2.140742898	2.50E-12	Cfhr1	22.28674383	3.58E-05	Lrrk1	0.585357244	0.004356
			Slc6a14	-2.132428542	0.008261511	Glyat	22.22654877	4.73E-16	Ppp1r13l	0.585357244	0.002678
			Cfap74	-2.027934365	0.000185901	F9	22.10241513	4.40E-05	Tgfbr2	0.533091047	0.005301
			Pde4c	-1.98684963	0.005686249	Ftcd	22.06658389	4.45E-05	Usp24	0.533091047	0.007959
			Gm47405	-1.881242676	0.005256702	Mbl1	22.06650087	4.45E-05	Acot11	0.529095126	0.005727
			Mroh6	-1.874029622	0.001754077	Klkb1	22.05323524	5.54E-26	Tspan15	0.529095126	0.005806
			Vmn2r-ps126	-1.796642296	0.002359094	Semg1	21.76713508	6.35E-05	Prkag2	0.46130771	0.005962
			Gm16299	-1.580550097	0.00737038	Slc22a26	21.53892663	8.38E-05	Dpep1	0.419703522	0.004552
			5830444B04Rik	-1.545014347	0.005013555	Mug-ps1	21.42550691	9.56E-05	Zfyve21	0.413136477	0.001174
			Gm45540	-1.542364751	0.004180707	Lect2	21.31989628	8.72E-27	Prr15	0.402255725	0.001836
			Smim9	-1.485644991	0.002458786	Mup7	21.27171521	0.000113692	Fbxl6	0.19265125	0.004539

Supplementary Methods

Antibodies and other reagents
The following primary antibodies were used to detect septins, junctional, cytoskeletal and leukocyte proteins: SEPT9 (Sigma-Aldrich, St. Louis, MO, Cat# HPA042564, for immunoblotting and immunofluorescence and Proteintech, Rosemont, IL, Cat# 10769-1-AP for immunohistochemistry, SEPT7 (Sigma-Aldrich, Cat# HPA023308 or IBL, Cat# 18991), SEPT2 (Sigma-Aldrich, Cat# HPA018481), SEPT6 (Proteintech, Cat# 12805-1-AP), SEPT11 (Proteintech, Cat# 14672-1-AP), ZO-1 (Proteintech, Cat# 21773-1-AP, for immunoblotting, ThermoFisher Scientific, Waltham, MA, Cat# 33-9100 for immunofluorescence labeling), occludin (Proteintech, Cat# 13409-1-AP), claudin-7 (Thermo Fisher, Cat# 34-9100), claudin-3 (Thermo Fisher, Cat# 34-1700), NMIIA (BioLegend, San Diego, CA, Cat# 909801), ZO-1 (Thermo Fisher, Cat# 40-2200), myeloperoxidase (Abcam, Cambridge, UK, Cat# ab9535), Ki-67 (Abcam, Cat# ab15580), mucin 2 (Abcam, Cat# ab272692), GAPDH (Cell Signaling Technology, Danvers, MA, Cat# 2118S), E-cadherin (R&D Systems, Minneapolis, MN, Cat# AF648 for human tissue immunolabeling, BiCell Scientific, Maryland Heights, MO, Cat# 00101 for mouse tissue immunofluorescence, Thermo Fisher, Cat# PA5-32178 for human cells immunofluorescence), NMIIC (Cell Signaling Technology, Cat# 8189S for immunoblotting, Thermo Fisher, Cat# PA5-64345 for immunofluorescence), β-catenin (BD Biosciences, Franklin Laker, NJ, Cat# 610153), CD4 (BD Biosciences, Cat# 550278), F4/80 (Bio-Rad Laboratories, Hercules, CA, Cat# MCA497GA,) AlexaFluor-488/555/568/647-conjugated secondary antibodies, were obtained from Thermo Fisher Scientific. Horseradish peroxidase-conjugated (HRP) goat anti-rabbit and anti-mouse secondary antibodies were acquired from Bio-Rad Laboratories. Fluorescein isothiocyanate (FITC)-4 kDa dextran (cat# FD4) and Rhodamine B isothiocyanate-70 kDa dextran (cat# R9379) were purchased from Sigma-Aldrich. The ApopTag® Fluorescein in Situ Apoptosis Detection kit was obtained from Sigma-Aldrich (Cat# S7110). All other chemicals were obtained from Sigma-Aldrich or Thermo Fisher.

Generation of SEPT9-mNeonGreen reporter mice
The genetic knock-in mice were generated by inserting the mNeonGreen (mNG) tag into the C-terminus of the SEPT9 sequence. The mice were inbred over three generations, and the fluorescence tag was checked under the fluorescent microscope.

Generation of Tamoxifen-inducible, Intestinal Epithelium-specific Knockout Mice
Inducible, intestinal epithelium-specific knockout mice ($SEPT9^{fl/fl}Vil1^{CreERT2}$, SEPT9-KO) were generated by crossing with $SEPT9^{fl/fl}$ strain (provided by Dr. Ernst-Martin Füchtbauer, University of Aarhus, Denmark) and $Vil1^{CreERT2}$ strain (The Jackson Laboratory, Bar Harbor, ME, stock # 020282). For the $SEPT9^{fl/fl}$ mice, a loxP site was inserted upstream of exon 2. An FRT-flanked neomycin resistance cassette with a 5' loxP site was inserted downstream of exon 5. Flp-mediated recombination removed the neomycin resistance cassette and left exons 2 through 5 floxed (1). $Vil1^{CreERT2}$ transgenic mice express a tamoxifen-inducible Cre expression driven by the 9-kb mouse Vil1 promoter. When crossed with a strain containing a LoxP site flanked sequence of interest, the offspring are useful for generating tamoxifen-induced, Cre-mediated targeted deletions. Tamoxifen induces Cre recombination in the intestinal epithelia of the duodenum, jejunum, ileum, proximal and distal colon (2). Tamoxifen administration was carried out according to the established protocol (3). Specifically, animals received intraperitoneal injections of tamoxifen (10 mg/ml; 100 microlites per mice) for 5 consecutive days. The animals were used for experiments or tissue collections on days 7-14 after the last tamoxifen injections. The efficiency of SEPT9 knockout was validated by immunoblotting of colonic epithelial scrapes obtained from SEPT9-KO and control animals. The animal colonies were maintained under pathogen-free conditions in the vivarium of the Lerner Research Institute of Cleveland Clinic and University of Virginia School of Medicine. Standard feed and tap water were available, *ad libitum*. The mouse room was on a 12 h light/dark cycle (lights on at 7:00 A.M.). At the beginning of colitis experiments, mice weighed 20-25 g, with no meaningful difference between the body masses of mice of different genotypes. All procedures were conducted under an animal research protocol approved by the Lerner Research Institute (IACUC protocol # 00001872) and University of Virginia Animal Care and Use Committees (Protocol# 4382) in accordance with the National Institutes of Health Animal Care and Use Guidelines.

Induction and characterization of dextran sodium sulfate (DSS) colitis

Experimental colitis was induced in 8–10-week-old SEPT9-KO mice and control $SEPT9^{fl/fl}$ littermates by administering a 3% (w/v) solution of DSS (molecular weight range 35,000-50,000 kDa; Thermo Fisher, Cat# J14489.22) in drinking water. Unchallenged animals received tap water *ad libitum*. Both male and female mice were used at roughly equal numbers. Animals were weighed daily and monitored for signs of intestinal inflammation. The disease activity index was calculated as previously described, by averaging numerical scores of body weight loss, stool consistency, and intestinal bleeding (4). With regards to body weight, no weight loss was scored as 0, loss of 1-5% was scored as 1; 5-10% weight loss as 2; 10-15% as 3, and more than 15% weight loss was scored as 4. For stool consistency, well-formed pellet was scored as 0, soft and semi-formed stool as 2, and liquid stool or diarrhea scored as 4. For intestinal bleeding, no blood was scored as 0, hemoccult-positive stool as 2, and gross rectal bleeding was scored as 4. On day 7 of DSS administration, animals were euthanized, with their colonic tissue harvested and separated into several segments. The samples were either fixed in a 4% formaldehyde solution, snap-frozen in liquid nitrogen or embedded in an optimal cutting temperature medium and snap-frozen for subsequent histological and biochemical examination. Formalin-fixed samples were paraffin-embedded, sectioned, and stained with hematoxylin and eosin (H&E). The tissue injury index was calculated based on microscopic examination of H&E sections, as previously described (5). The H&E-stained slides were scored by a gastrointestinal pathologist in a blinded fashion. The index represents the sum of individual scores reflecting severity and extent of inflammation, extent of crypt damage and percentage of the involved area.

Measuring intestinal permeability in vivo using fluorescently labeled tracers

The in vivo intestinal permeability assay was performed in SEPT9-KO and Control mice following the method described previously (6). Food-deprived animals were given a mixture of FITC-labeled dextran (4 kDa; 80mg/100 g body weight) and rhodamine-labeled dextran (70 kDa; 40mg/100g body weight) dissolved in phosphate-buffered saline (PBS). Control mice received PBS only. Animals were euthanized 3 hours later for blood collection via cardiac puncture, blood serum was obtained by centrifugation, and FITC and Rhodamine fluorescence intensities were measured using a Biotech Synergy H1 plate reader (Agilent Technology, Santa Clara, CA) with excitation and emission wavelengths at 495/525nm and 555/585nm, respectively. The fluorescence intensity of dextran-free serum was subtracted from each measurement. The concentration of fluorescent tracers in blood serum was calculated using the standard curves prepared via serial dilutions of stock solutions of FITC-dextran and Rhodamine-dextran in PBS.

Cell culture

HT-29 clone cF8, a well-differentiated clone of HT-29 human colonic epithelial cells, were provided by Dr. Judith M Ball (College of Veterinary and Biomedical Sciences, Texas A&M University, College Station, TX)(7, 8). DLD-1 (Cat# CCL-221) and Caco-2BBE (Cat# CRL-2102) human colonic epithelial cells and COS-7 fibroblasts (Cat# CRL-1651) were obtained from the American Type Culture Collection (ATCC, Manassas, VA). DLD-1, HT-29 and Caco-2BBE cells were cultured in DMEM (ATCC, Cat# 30-2002) medium and COS-7 cells were cultured in DMEM/F-12 medium (Thermo FisherCat# 11330032). All the culture media were made according to the complete medium recipe on ATCC with penicillin-streptomycin antibiotic. The cells were seeded on Costar Transwell filters (Corning Incorporated, Kennebunk, ME, Cat# 3415), or glass coverslips for permeability measurements and immunolabeling experiments; and on 6-well plastic plates for other functional and biochemical studies. All cultured cells were mycoplasma-free according to the mycoplasma PCR detection assay (PromoCell, Heidelberg, Germany, Cat# PK-CA20-700-20). Different cell models were used in this study for the following reasons: HT-29 cF8 cells served as the primary intestinal epithelial model suitable for CRISPR-Cas9–mediated SEPT9 knockout. These cells rapidly polarize and form barrier within 6 days after plating. Caco-2BBE cells provided an additional polarized model in which we successfully deleted NMIIC. These cells were cultured for up to 11 days after plating to promote junctional assembly and barrier formation; DLD-1 cells were used because they readily assemble apical junctions and allow efficient plasmid transfection. COS-7 cells, which do not express NMIIC, were selected for reconstitution experiments and high-resolution imaging.

SEPT9-NG in vivo pulldown and co-IP experiments

For in vivo pulldown of SEPT9-associated proteins, colonic epithelial tissue was harvested from $SEPT9^{NG}$ knock-in mice or $SEPT9^{fl/fl}$ mice as a negative control to assess nonspecific binding. Tissues were homogenized in ice-cold lysis buffer (50 mM Tris-HCl pH 7.5, 150 mM NaCl, 1% NP-40, 1 mM EDTA, 10% glycerol) supplemented with protease and phosphatase inhibitors (Roche, Basel, Switzerland, Cat# 11836170001). Lysates were cleared by centrifugation (16,000 × g, 15 min, 4°C), and equal amounts of protein (500–800 µg) were incubated overnight at 4°C with NeonGreen-Trap magnetic agarose beads (Proteintech) to immunoprecipitate SEPT9-NG complexes. After washing, bound proteins were eluted in SDS sample buffer and analyzed by SDS–PAGE and western blotting. Antibodies used for immunoblotting included anti-SEPT9 (Proteintech), anti-NMIIC (Thermo Fisher), and anti-SEPT7 (IBL) as a positive control and known interactor of SEPT9.

Plasmid transfection

The COS-7 cells were cultured on coverslips until 50%~60% confluency and transfected with Lipofectamine 2000 (Thermo Fisher, Cat# 11668019). Briefly, the Opti-MEM medium (Thermo Fisher, Cat# 31985070,) was pre-mixed with 1.6 µg of target plasmid DNA (SEPT9v2-GFP, Origene, Rockville, MD Cat# RG225979 and NMIIC-mCherry, Addgene, Watertown, MA, Cat# 55108) or 4µL Lipofectamine 2000. The diluted plasmid DNA and Lipofectamine 2000 were combined and incubated for 10min at room temperature. The mixture was then added to the cells and incubated for 5 hours. The medium was then replaced with fresh medium, and the cells were further cultured for 16 hours before harvesting and imaging.

Cell proliferation assay

The cell proliferation profiles were measured by either cell counting using hemocytometer or by the MTT assay using the MTT cell growth assay kit (Sigma-Aldrich, Cat# CT01) according to the manufacturer instructions. The MTT optical density was measured using the Biotech Synergy H1 plate reader. The cells numbers and MTT absorbance were determined daily for 4 days after cell plating.

Quantitative real-time RT-PCR analysis of inflammatory gene expression in mouse colonic tissues

Total RNA was isolated from distal colonic segments of SEPT9-KO and Control animals using a RNeasy mini kit (Qiagen) followed by DNase treatment to remove genomic DNA. Total RNA (1 µg) was reverse transcribed using an iScript cDNA synthesis kit (Bio-Rad Laboratories). Quantitative real-time RT-PCR was performed using iTaq Universal SYBR Green Supermix (Bio-Rad Laboratories) in a CFX96 Real-time PCR System (Bio-Rad Laboratories). The threshold cycle number (Ct) for specific genes of interest and a housekeeping gene was determined based on the amplification curve representing a plot of the fluorescent signal intensity versus the cycle number. The relative expression of each gene was calculated by a comparative Ct method based on the inverse proportionality between Ct and the initial template concentration (2-ΔΔCt), as previously described (9). This method is based on two-step calculations of ΔCt = Cttarget gene - CtGAPDH and ΔΔCt = ΔCte-ΔCtc, where index e refers to the sample from any DSS or water-treated SEPT9-KO, or Control mice, and index c refers to the sample from a water-treated control animal assigned as an internal control. The primer sequences are described in our published study (10)

Immunoblotting analysis

Mouse colonic segments were harvested, longitudinally dissected, opened, and washed with ice-cold PBS. Epithelial cells were collected by gently scraping the exposed interior with razor blades, then snap frozen in liquid nitrogen for further analysis. Intestinal epithelial scrapings were lysed and homogenized in RIPA buffer containing a protease inhibitor cocktail, phosphatase inhibitor cocktails 2 and 3, and phenylmethylsulfonyl fluoride (all from Sigma-Aldrich). The samples were diluted with the 2x SDS sample loading buffer and boiled. Total lysates of cultured human IEC cells were prepared similarly to the mouse tissue lysates. SDS-polyacrylamide gel electrophoresis was conducted using standard protocols with an equal amount of total protein loaded per lane (10 µg), followed by transfer to a nitrocellulose membrane at 4°C for either 1 or 12 h depending on protein size. After transfer, the membranes were blocked in 5% TBST-20 buffered skim milk solution for 1 h at room temperature and then incubated with primary antibodies either for 1 hour at room temperature or overnight at 4°C. Primary antibodies were used at 1:500 or 1:1,000 dilution. Membranes were washed, incubated with HRP-conjugated

secondary antibodies (1:10,000 dilution) at room temperature, and the labeled proteins were visualized using standard enhanced chemiluminescence reagents (Sigma-Aldrich) and X-ray films. Protein expression was quantified via densitometry using ImageJ 1.51K software (National Institutes of Health, Bethesda MD). Signal intensities of each protein were normalized by the signal intensity of a housekeeping protein, GAPDH, in the same sample. The data are presented as normalized values, where expressions of either a reference control mouse sample or a human control sgRNA sample were taken as 1.

Affinity mass spectrometry of SEPT9 interactome

DLD-1 cells were grown to sub-confluency and transfected with human SEPT9-GFP plasmid (OriGene, Cat# RG225682). The transfected cells were harvested 24 h after transfection and lysed. SEPT9-GFP protein was purified by coimmunoprecipitation, digested with trypsin, and desalted using ChromoTek iST GFP-Trap IP-MS sample preparation kit (Proteintech, Cat gtak-iST). Desalted samples were analyzed in duplicate by nanoLC-MS/MS using a Dionex Ultimate 3000 (Thermo Fisher Scientific) coupled to an Orbitrap Eclipse Tribrid mass spectrometer (Thermo Fisher Scientific). Peptides were loaded onto an Acclaim PepMap 100 trap column (300 µm × 5 mm × 5 µm C18) and gradient-eluted from an Acclaim PepMap 100 analytical column (75 µm × 25 cm, 3 µm C18) equilibrated in 96% solvent A (0.1% formic acid in water) and 4% solvent B (80% acetonitrile in 0.1% formic acid). The peptides were eluted into the mass spectrometer at 300 nL/min up to 90% B over a period of 2 hours. MS2 spectra were acquired using a data-dependent instrument method with the following settings: positive ion mode was used with 2.0 kV at the spray source, RF lens at 30% and data dependent MS/MS acquisition with XCalibur version 4.3.73.11. Full MS scans were acquired in the Orbitrap from 375 to 1500 *m*/*z* with 120,000 resolution. Data dependent selection of precursor ions was performed in Cycle Time mode, with three seconds in between Master Scans, using an intensity threshold of 2e4 ion counts and applying dynamic exclusion ($n = 1$ scans within 30 s for an exclusion duration of 60 s and ±10 ppm mass tolerance). Monoisotopic peak determination was applied, and charge states 2–6 were included for HCD MS2 scans (quadrupole isolation mode; 1.6 *m*/*z* isolation window, normalized collision energy at 30%). The resulting fragments were detected in the Orbitrap at 15,000 resolution with standard AGC target and dynamic maximum injection time mode. Raw MS data was searched using Proteome Discoverer against a custom fasta database that contained all Gencode v41 protein sequences as well as SEPT9 isoform sequences. The following parameters were used: trypsin with maximum 2 missed cleavage sites, 10 ppm precursor mass tolerance and 0.02 Da fragment ion mass tolerance, dynamic amino acid modifications (Oxidation / +15.995 Da (M) and Phospho / +79.966 Da (S, T, Y)) and dynamic n-terminal modifications (Acetyl / +42.011 Da, Met-loss / -131.040 Da (M), and Met-loss+Acetyl / -89.030 Da (M)). A static modification was used on Cysteine (Carbamidomethyl / +57.021 Da). To identify SEPT9-specific interactors, we performed parallel immunoprecipitation-mass spectrometry using GFP-tagged Rab11, a vesicle-associated protein with no known interaction with septins, as a negative control.

Examining leukocyte infiltration cell proliferation and death in mouse tissues by fluorescence labeling and confocal microscopy

Immunofluorescence labeling of the T-cell marker, CD4, the macrophage marker, F4/80, and the proliferation marker Ki-67 were performed using frozen sections of mouse colonic mucosa. The sections were fixed with absolute ethanol at -20°C for 20 minutes and rinsed 3 times in cold PBS. The sections were blocked for 60 minutes at room temperature in Hanks HEPES-buffered salt solution containing 1% bovine serum albumin, followed by overnight incubation at 4°C with primary antibodies. Samples were then washed and incubated with Alexa dye-conjugated secondary antibodies for 60 minutes, then rinsed with blocking buffer. For MPO staining, and TUNEL labeling, formalin-fixed paraffin embedded tissue sections were deparaffinized following by the antigen retrieval using a R-Universal Epitope Recovery Buffer (Electron Microscopy Sciences Hatfield, PA, Cat# 62719-20). The sections were blocked for 60 minutes in 1% bovine serum albumin in Hanks HEPES-buffered and stained as described above. TUNEL labeling was performed using the ApopTag Fluorescein in Situ Apoptosis Detection Kit, according to the manufacturer's instructions. All labeled samples were mounted on slides using ProLong Antifade mounting reagent with DAPI (Thermo Fisher Scientific, Cat #P36941). Fluorescently labeled tissue sections were imaged using Leica HCX PL APO 40xPH3CS (1.25 NA) OIL immersion objective and Leica TCS SP8 AOBS confocal laser scanning system attached to a Leica DMi8 inverted epifluorescence microscope

(Wentzler, Germany). The Alexa Fluor 488 and 555 signals were acquired sequentially in frame-interlace mode, to eliminate cross talk between channels.

Immunofluorescence labeling of SEPT9 in human intestinal tissue samples
Surgically resected full-thickness sections of human intestine were fixed in 10% neutral-buffered formalin for 24h. After fixation, formalin was replaced with a 10% sucrose solution for 2h and dehydration was continued by sequential incubation of the samples with 20% and 30% sucrose solutions for 2h each. Finally, the samples were transferred into 50% OCT/30% sucrose solution, embedded into OCT and snap frozen on dry ice. The frozen blocks were used to prepare 10 μm sections by using a Leica cryostat and the sections were processed for immunofluorescence labeling. Briefly, frozen sections were permeabilized with 0.5% of Triton-X100 for 5 min at room temperature, blocked for 60 min in a blocking buffer (PBS containing 1% bovine serum albumin, pH 7.4) followed by 60 min incubation with anti-SEPT9 rabbit polyclonal antibody and anti E-cadherin goat polyclonal antibody diluted in the blocking buffer (1:200 dilution). Afterwards, the samples were washed three times with the blocking buffer, incubated with Alexa-Fluor-488–conjugated donkey anti-rabbit and AlexaFluor-647–conjugated donkey anti-goat secondary antibodies at 1:1,000 dilution in the blocking buffer for 60 min, rinsed three times with the blocking buffer, and mounted on slides with ProLong™ Gold Antifade mounting medium with DAPI. Immunofluorescence labeled tissues were imaged using Leica 100x CS2 (1.4NA) OIL immersion objective and Leica TCS SP8 AOBS confocal laser scanning system attached to a Leica DMi8 inverted epifluorescence microscope (Wentzler, Germany). The Alexa Fluor 488 and 647 signals were acquired sequentially in frame-interlace mode, to eliminate cross-talk between channels.,

Immunohistochemical (IHC) labeling of SEPT9 in paraffin embedded samples
Harvested human colon samples were paraffin-embedded, sectioned at 5μm thickness, and stained for SEPT9 (Proteintech, Cat# 10769-1-AP,) as described previously (11). Briefly, the slides were blocked using 10% normal goat serum and incubated with the primary antibodies overnight at 1:200 dilution at 4°C. The slides were incubated with a biotinylated universal secondary antibody and then streptavidin-peroxidase complex (Vector Laboratories, Newark, CA, Cat# PK-7800,) followed by staining with 3,3′-diaminobenzidine (Thermo Fisher Scientific, Cat# 34002,) and counter-staining with hematoxylin (Sigma-Aldrich, Cat# 51275). Finally, the finished slides were dehydrated in gradient ethanol and xylene and mounted in a mounting medium. An optical microscope captured the non-overlapping images from the intestinal mucosa. The percentage of positively stained area for the markers was determined using ImageJ (version 2.0.0, National Institutes of Health).

Bulk RNA sequencing of mouse intestinal epithelial cells.
Ileal and colonic epithelial cells were isolated from SEPT9-KO and control mice by the sequential dithiothreitol/EDTA extraction protocol as described previously (12, 13). Total RNA was extracted using the Qiagen RNeasy Plus Mini kit (Qiagen, Cat#74134), using a modified version of the manufacturer's protocol. Sample lysates were added to QIAShredder columns and centrifuged, and the homogenized lysates were added to gDNA eliminator columns. The columns were centrifuged, and the lysates were combined with ethanol; the samples were then added to RNeasy Plus Mini spin columns and centrifuged. The membranes were washed six times with RPE buffer and centrifuged after each wash. RNA was eluted using 25 μl of nuclease-free water. The extracted RNA concentrations and the 260/280 absorption ratio were measured using the Qubit assays and Nanodrop. The Total RNA libraries were prepared in the LRI Genomic Core following the manufacturer's protocol (Illumina, Document #1000000124514). In brief, ribosomal RNA was depleted, and the RNA underwent fragmentation and denaturation. Subsequently, cDNA was synthesized, followed by end repair, adenylation, and adaptor ligation, culminating in PCR amplification. The libraries were evaluated for quality, quantified, and sequenced at 20 million reads per sample on the Illumina sequencer Novaseq 6000.

Bioinformatic analysis of bulk RNAseq data
Raw sequencing reads were initially assessed for quality using FastQC (FastQC v0.12.1). Following quality assessment, reads were quality-trimmed to remove low-quality bases and adapter sequences using Trimmomatic (version v0.39). The parameters used for trimming were set as follows: a minimum quality score of 20 and a sliding window of 4 bases. The trimmed reads were subsequently re-evaluated for quality using FastQC to confirm

that all quality metrics met the acceptable thresholds. To eliminate contaminating host (GRCm39 (Mouse Genome Reference Consortium, Version 39) reads, we utilized BBMap (version 38.99). This step involved aligning the trimmed reads against a mouse reference genome (source) to remove any reads originating from the host. The "filterbyname" option retained only non-host reads for downstream analysis. This ensured that the subsequent mapping and analysis primarily focused on the target transcriptome without interference from host sequences. The cleaned reads were mapped to the mouse genome using the STAR aligner (version 2.7.10.A). STAR was chosen for its speed and accuracy in handling RNA-seq data. Mapping parameters were optimized to include two-pass mapping to improve splice site detection and reduce the number of multi-mapping reads. The output from STAR included aligned reads in BAM format. The count matrix was generated from the STAR output using the featureCounts function from the Subread package in R (version 2.0.1). This step involved using the mouse genome's corresponding gene annotation file (GTF format). The featureCounts summarized the number of reads mapping to each gene, which was crucial for subsequent differential expression analysis. Differential abundance analysis was conducted using the DESeq2 package in R (version 1.38). The raw count data was normalized for differences in sequencing depth and library composition using the median of ratios method implemented in DESeq2. A negative binomial generalized linear model was used to identify differentially expressed genes (DEGs), and genes with an adjusted p-value of < 0.05 and a fold change ≥ 2 were considered significant. Pathway enrichment analysis was performed using the enrichR R package. Enrichment analysis was conducted with several pathway databases, including, GO, KEGG and Reactome, to identify biologically relevant pathways associated with the identified DEGs. The top enriched pathways were selected based on a p-value threshold of < 0.05. Results were visualized using ggplot2 (version 3.4.2), a powerful R package for data visualization. Key visualizations included heatmaps for normalized count data of the top DEGs, volcano plots depicting the significance and magnitude of expression changes, and pathway analysis plots illustrating the enriched biological processes. The sequence data were uploaded to the NCBI GEO database. Submission ID: SUB14911003; BioProject ID: PRJNA1195935.

Single cell RNAseq data analysis

Single-cell RNA alignment files from 6 non-IBD patients (paired ascending colon & terminal ileum) were downloaded from GEO database (GSE266616). Samples were aligned to the human reference genome (GRCh38-2020-A) using CellRanger v7.0.1. All downstream analysis was performed using Seurat v5.1.0 in R (v4.3.1). Cells with mitochondrial ratios less than 40% (removes low-quality/dying cells) and total RNA transcript counts less than 50k were removed (removes potential multi-plets). The remaining cells from each sample were normalized for sequencing depth using the NormalizeData function and variable features from each sample were identified using the FindVariableFeatures function. To reduce computational time and memory usage, a 'sketch' assay was generated using the top 5,000 candidate cells from each sample by calculating a 'LeverageScore' using the SketchData function. The 'sketch' assay was then integrated together to remove batch effects using the standard integration pipeline in Seurat to create a UMAP. A total of 19 clusters were identified that included immune cells & fibroblasts (11 clusters, deemed 'Other') and standard epithelial lineage cells (8 clusters, deemed 'Epithelial' by predominant expression of EPCAM). Marker genes for each cluster were determined using the wilcoxauc function from the 'presto' package (v1.0.0). Both the integrated UMAP coordinates, and cluster identification calculated for the 'sketch' assay were projected onto the full dataset for subsequent analysis. For further refinement and identification of epithelial subtypes, the 8 'Epithelial' clusters were subset off and ran through the same analysis pipeline, which resulted in 20 unique clusters.

Quantitative Image Analysis

An open-source software QuPath v.0.3.2 (16) was used to quantify the number of F4/80, CD4, MPO, Ki-67 and TUNEL positive cells. Whole microscopic images (at × 40 magnification) were imported to QuPath, and positive cells were detected using optical density sum in the red (or green for the TUNEL assay) and DAPI channels of the obtained images. For each type of labeling, the numbers of the red or green channel positive cells were counted in 5 different images per animal, and the obtained values were averaged. Tissue samples collected from 5-7 different animals per each experimental group were examined. The animal numbers for each experimental group are presented in the figure legends. To quantify the SEPT9 signal intensity at the cell-cell junctions in human tissue sections, the images were imported into ImageJ 1.51K, and a small rectangular area manually drawn over

the junctional contacts labeled by E-cadherin (converted to red color). The selected area was transferred over the SEPT9 image (green). The signal intensity of SEPT9 at the cell-cell contact junctions was measured along with signal intensity of the same rectangular area of the adjacent cytoplasmic region. The fluorescence intensity ratio between cell-cell junctions and the cytoplasm was calculated. To calculate the overall intensity of the SEPT9 signal, fluorescence intensities of the paired junctional and cytoplasmic areas were averaged. 10 different measurements were taken per each confocal image and 3 different images per each tissue sample were analyzed resulting in 30 measurements per patient sample that were averaged for one biological replicate. A one-way ANOVA with Bonferroni's post hoc test was used (to compare CD and UC patients; with the normal controls). p values < 0.05 were considered statistically significant and all statistical analysis was performed using GraphPad Prism 10.

Supplementary References

1. Füchtbauer A, et al. Septin9 is involved in septin filament formation and cellular stability. *Biol Chem*. 2011;392(8–9):769–777.

2. el Marjou F, et al. Tissue-specific and inducible Cre-mediated recombination in the gut epithelium. *Genesis*. 2004;39(3):186–193.

3. The Jackson Laboratory. Intraperitoneal Injection of Tamoxifen for Inducible Cre-Driver Lines [Internet]. *The Jackson Laboratory*. https://www.jax.org/research-and-faculty/resources/cre-repository/tamoxifen. Accessed August 30, 2024.

4. Rhee L, et al. Expression of TNFAIP3 in intestinal epithelial cells protects from DSS- but not TNBS-induced colitis. *Am J Physiol Gastrointest Liver Physiol*. 2012;303(2):G220-227.

5. Xuan L, et al. Cytomegalovirus Infection Exacerbates Experimental Colitis by Promoting IL-23 Production. *Inflammation*. 2020;43(1):326–335.

6. Chanez-Paredes SD, et al. Differentiating Between Tight Junction-Dependent and Tight Junction-Independent Intestinal Barrier Loss In Vivo. *Methods Mol Biol*. 2021;2367:249–271.

7. Lechuga S, et al. A myosin chaperone, UNC-45A, is a novel regulator of intestinal epithelial barrier integrity and repair. *FASEB J*. 2022;36(5):e22290.

8. Mitchell DM, Ball JM. Characterization of a spontaneously polarizing HT-29 cell line, HT-29/cl.f8. *In Vitro Cell Dev Biol Anim*. 2004;40(10):297–302.

9. Ivanov AI, et al. Prostaglandin E2-synthesizing enzymes in fever: differential transcriptional regulation. *American Journal of Physiology-Regulatory, Integrative and Comparative Physiology*. 2002;283(5):R1104–R1117.

10. Naydenov NG, et al. Nonmuscle Myosin IIA Regulates Intestinal Epithelial Barrier in vivo and Plays a Protective Role During Experimental Colitis. *Sci Rep*. 2016;6:24161.

11. Hu G, et al. Renomedullary exosomes produce antihypertensive effects in reversible two-kidney one-clip renovascular hypertensive mice. *Biochemical Pharmacology*. 2022;204:115238.

12. Zeineldin M, Neufeld K. Isolation of Epithelial Cells from Mouse Gastrointestinal Tract for Western Blot or RNA Analysis. *Bio Protoc*. 2012;2(22):e292.

13. Ge Y, Zadeh M, Mohamadzadeh M. Dissociation and flow cytometric isolation of murine intestinal epithelial cells for multi-omic profiling. *STAR Protoc*. 2023;4(1):101936.

14. Bankhead P, et al. QuPath: Open source software for digital pathology image analysis. *Sci Rep*. 2017;7(1):16878.
