# Supplementary material for: The septin cytoskeleton is a regulator of intestinal epithelial barrier integrity and mucosal inflammation
Source: JCI Insight. 2025 Oct 7;10(22):e191538. doi: 10.1172/jci.insight.191538 (PMC12643519; doi:10.1172/jci.insight.191538)
Supplement: Unedited blot and gel images [file jciinsight-10-191538-s124.pdf]

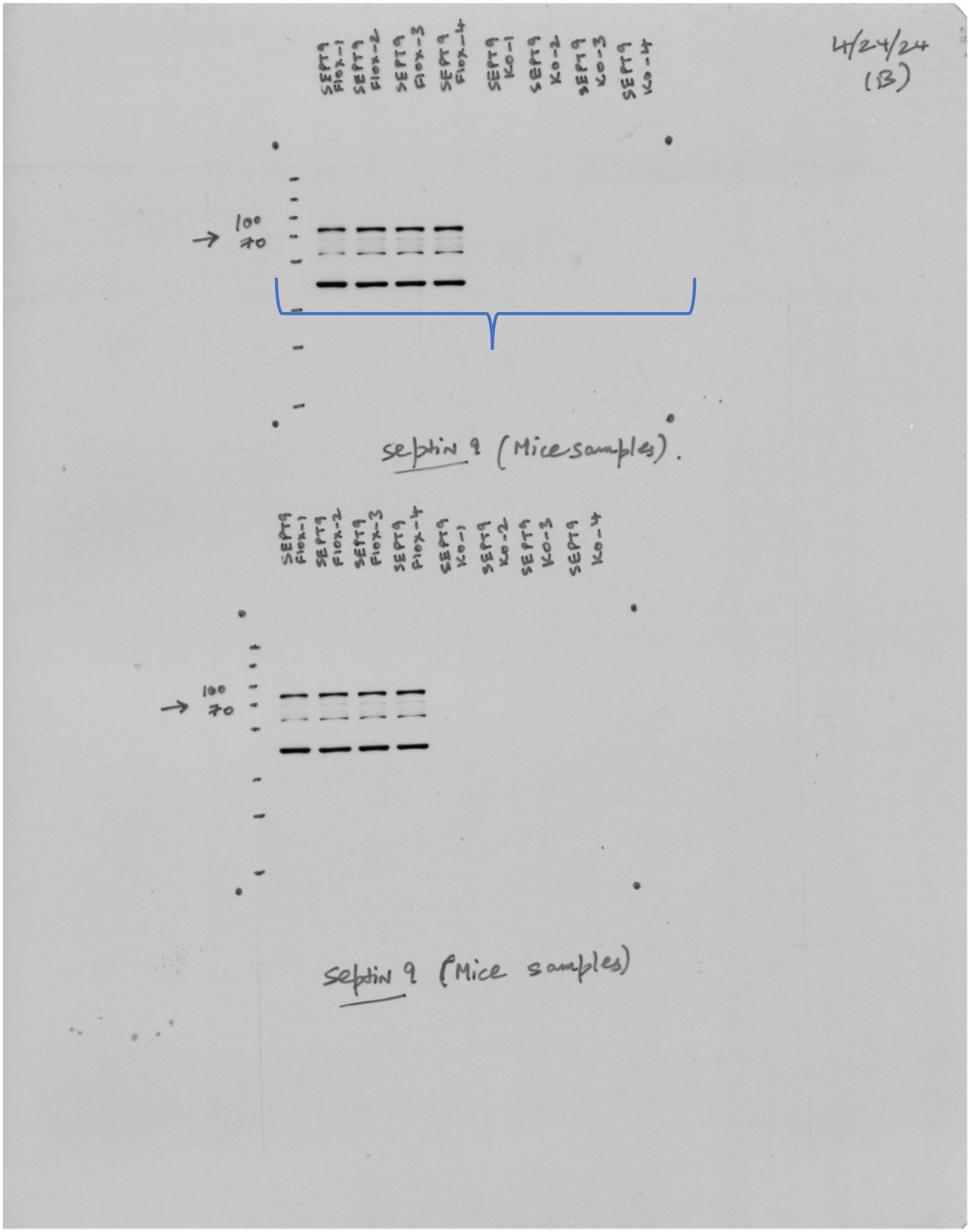

Full unedited gel for Figure 3A (Septin9)
Antibody Sigma-Aldrich HPA042564

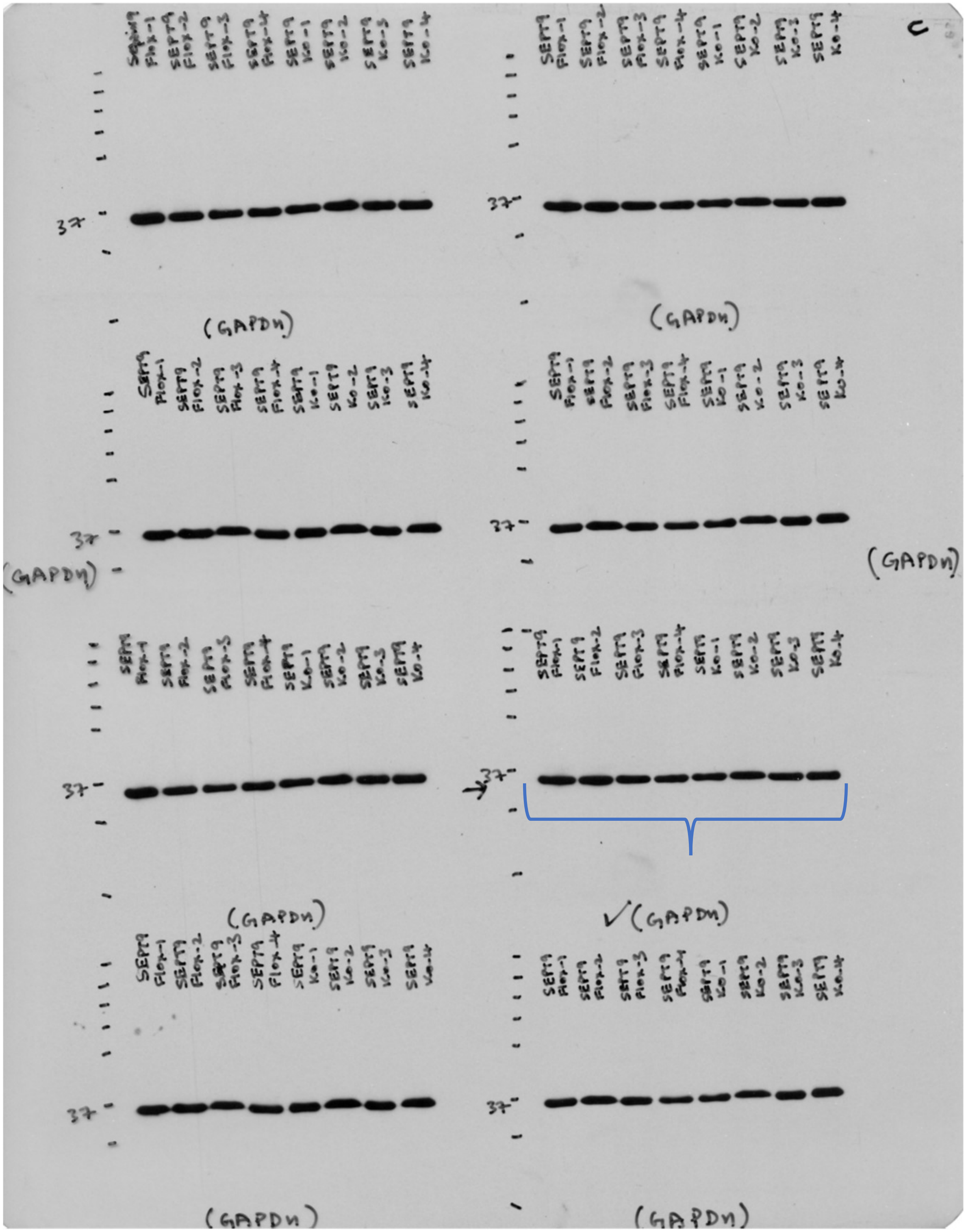

Full unedited gel for Figure 3A (GAPDH)
Antibody Cell Signaling Technology 2118S

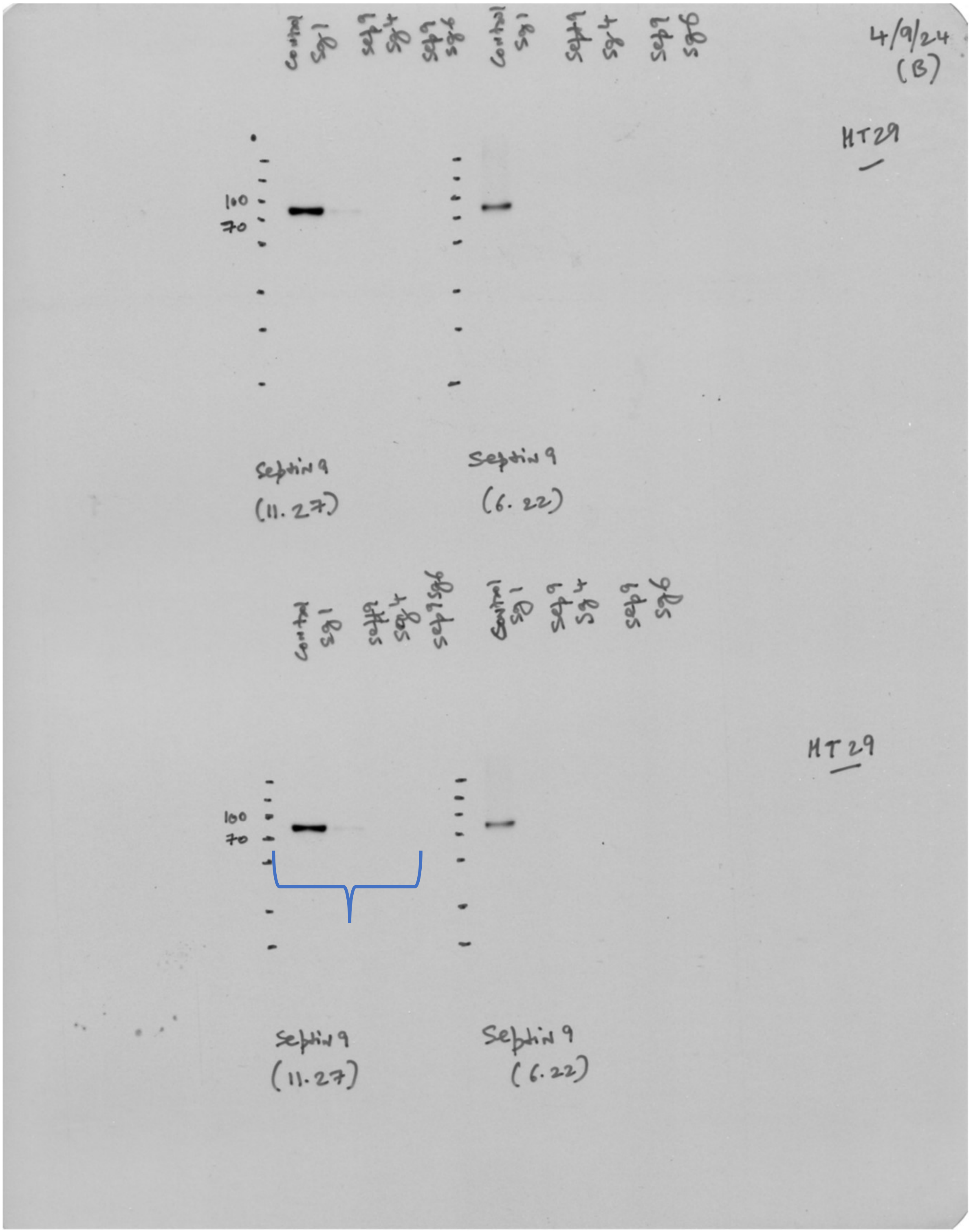

Full unedited gel for Figure 6A (Septin9)
Antibody Sigma Aldrich HPA042564

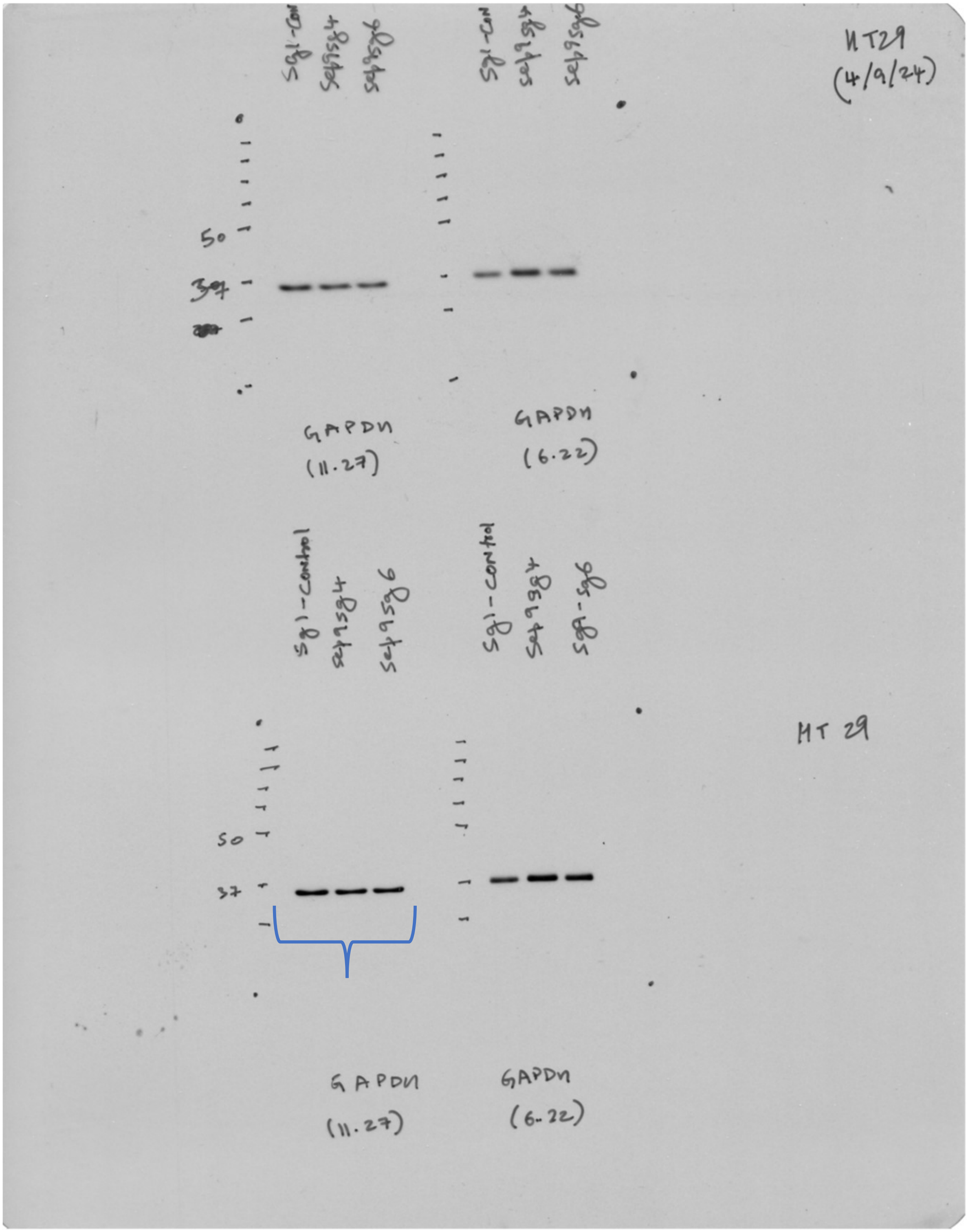

Full unedited gel for Figure 6A (GAPDH)
Antibody Cell Signaling Technology 2118S

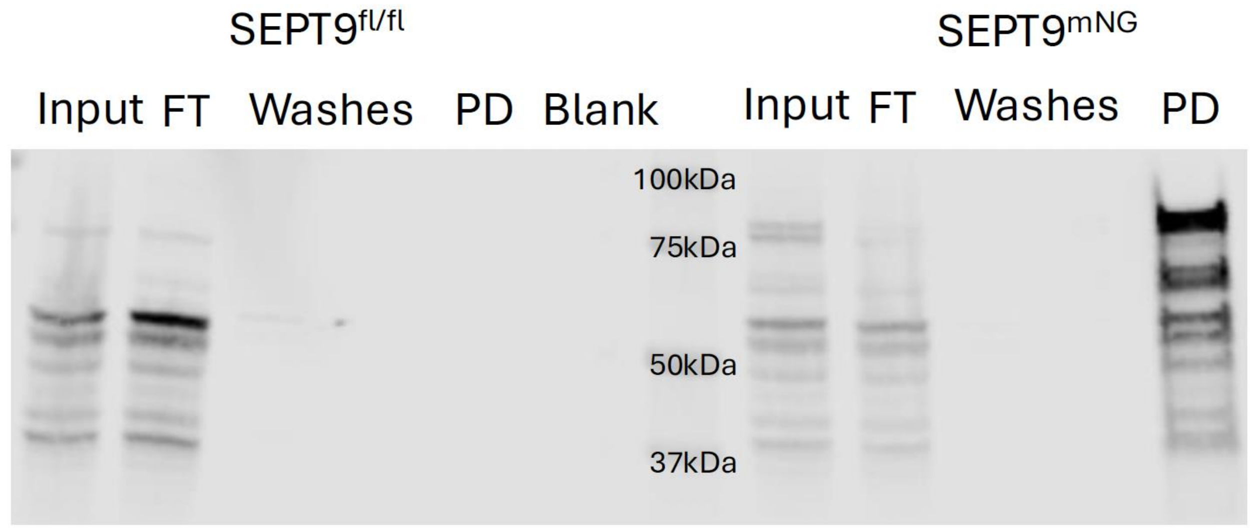

SEPT9-pAb, 1:1000 O/N 4C

Full unedited gel for Figure 7H (SEPT9)

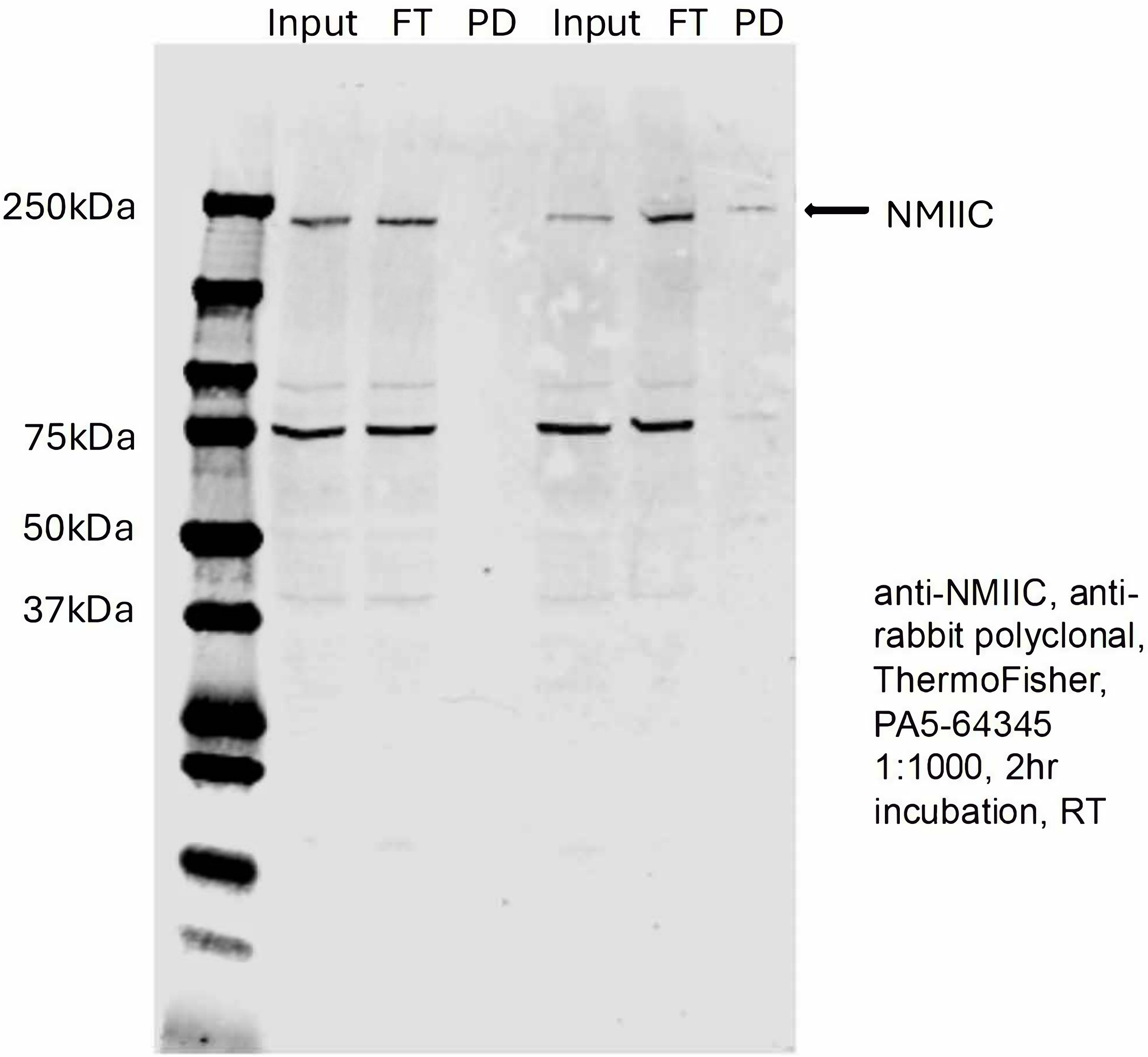

Full unedited gel for Figure 7H (NMIIC)

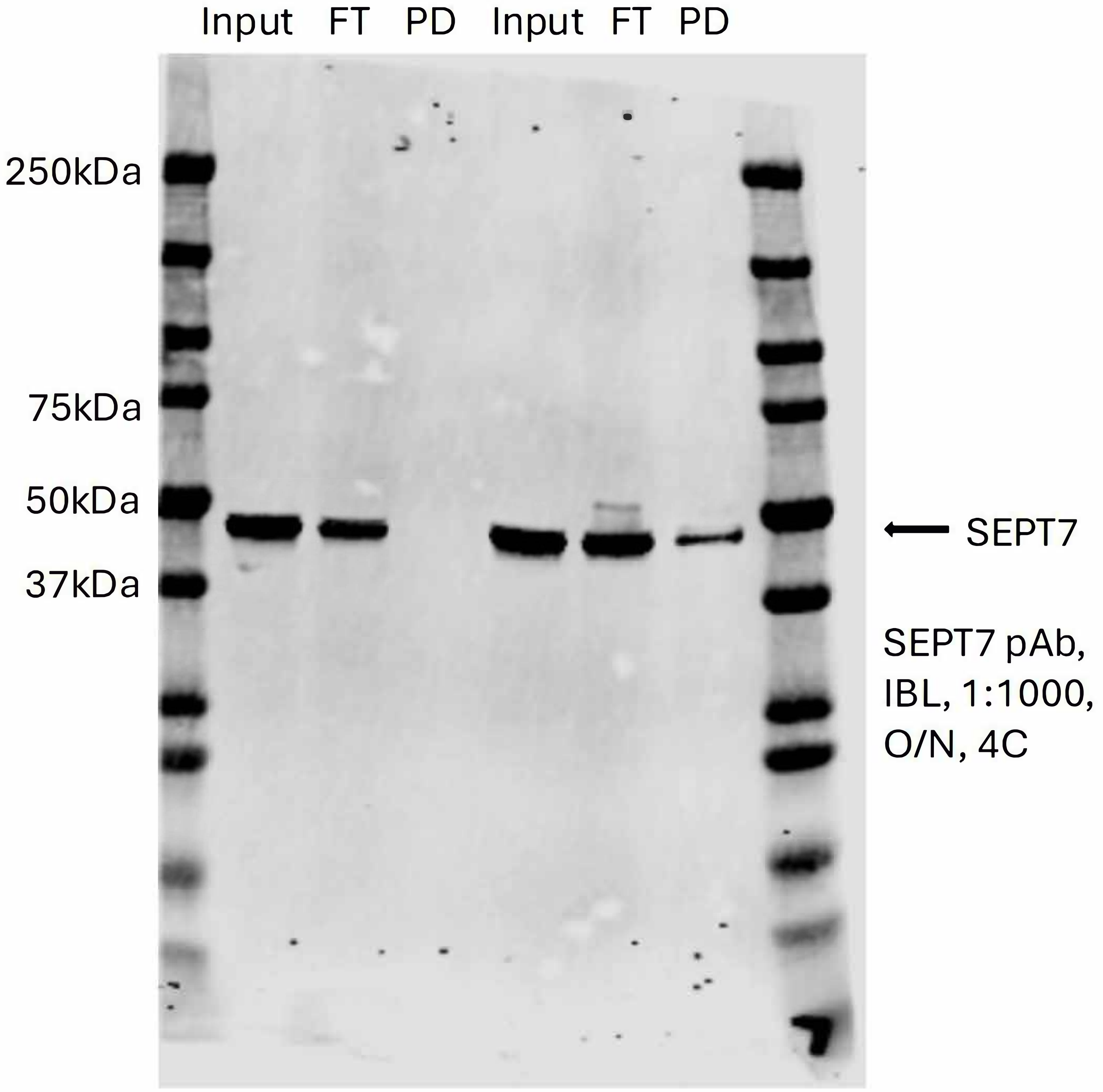

Full unedited gel for Figure 7H (SEPT7)

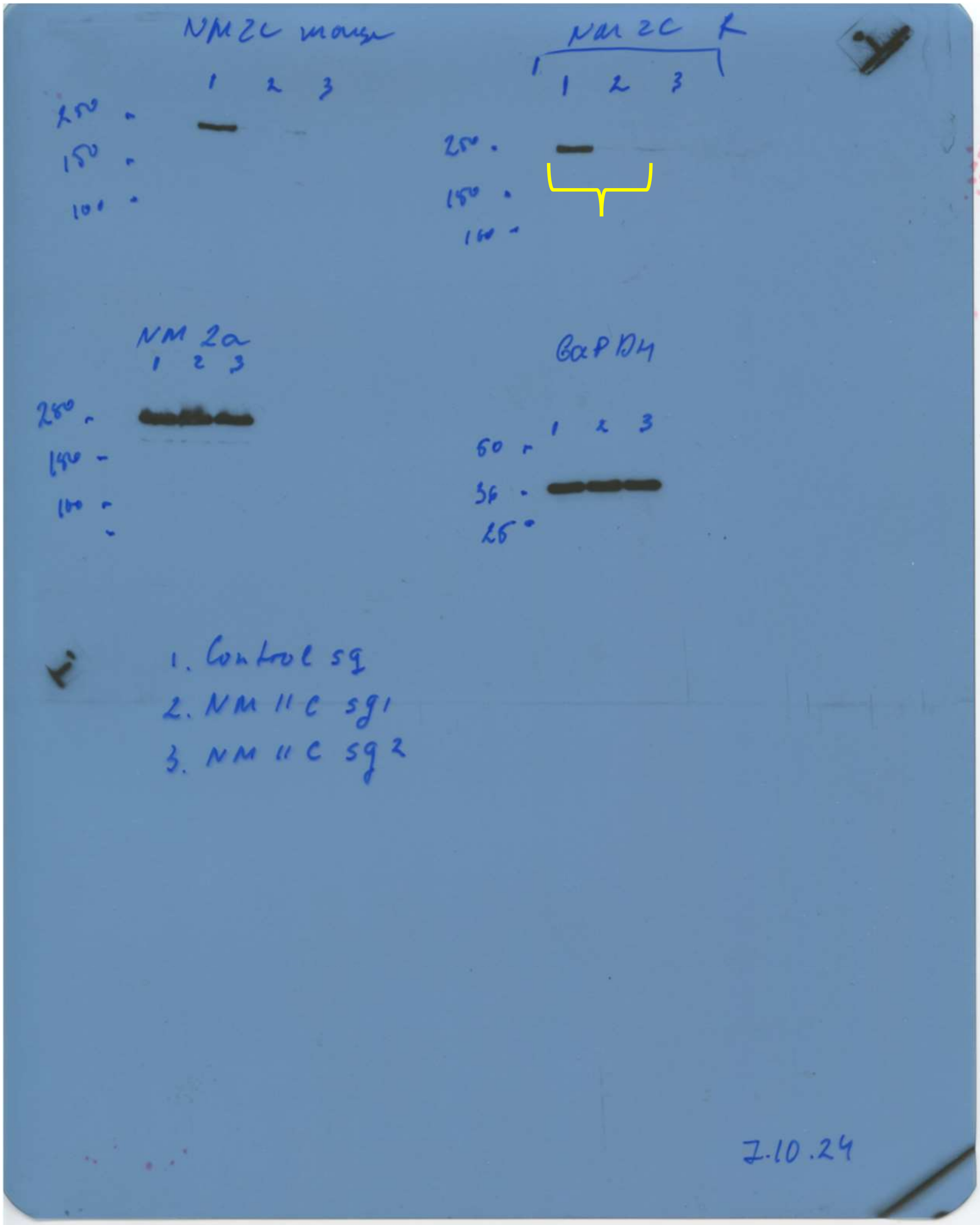

Full unedited gel for Figure 7I (NM IIC)
Antibody Cell Signaling Technology 8189S

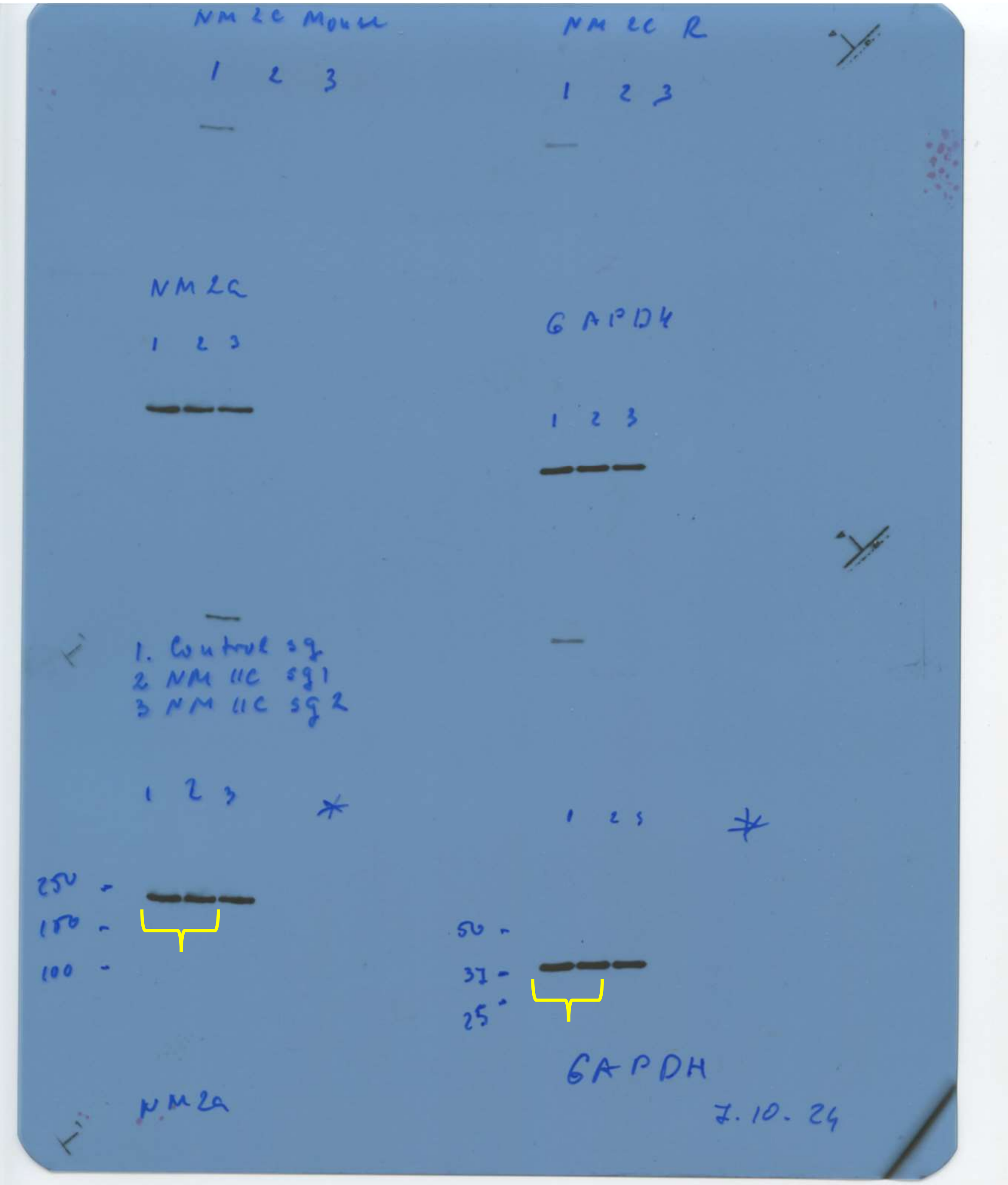

Full unedited gel for Figure 7I
(NM IIA)
Antibody BioLegend 909801

Full unedited gel for Figure 7I
(GAPDH)
Antibody Cell Signaling
Technology 2118S

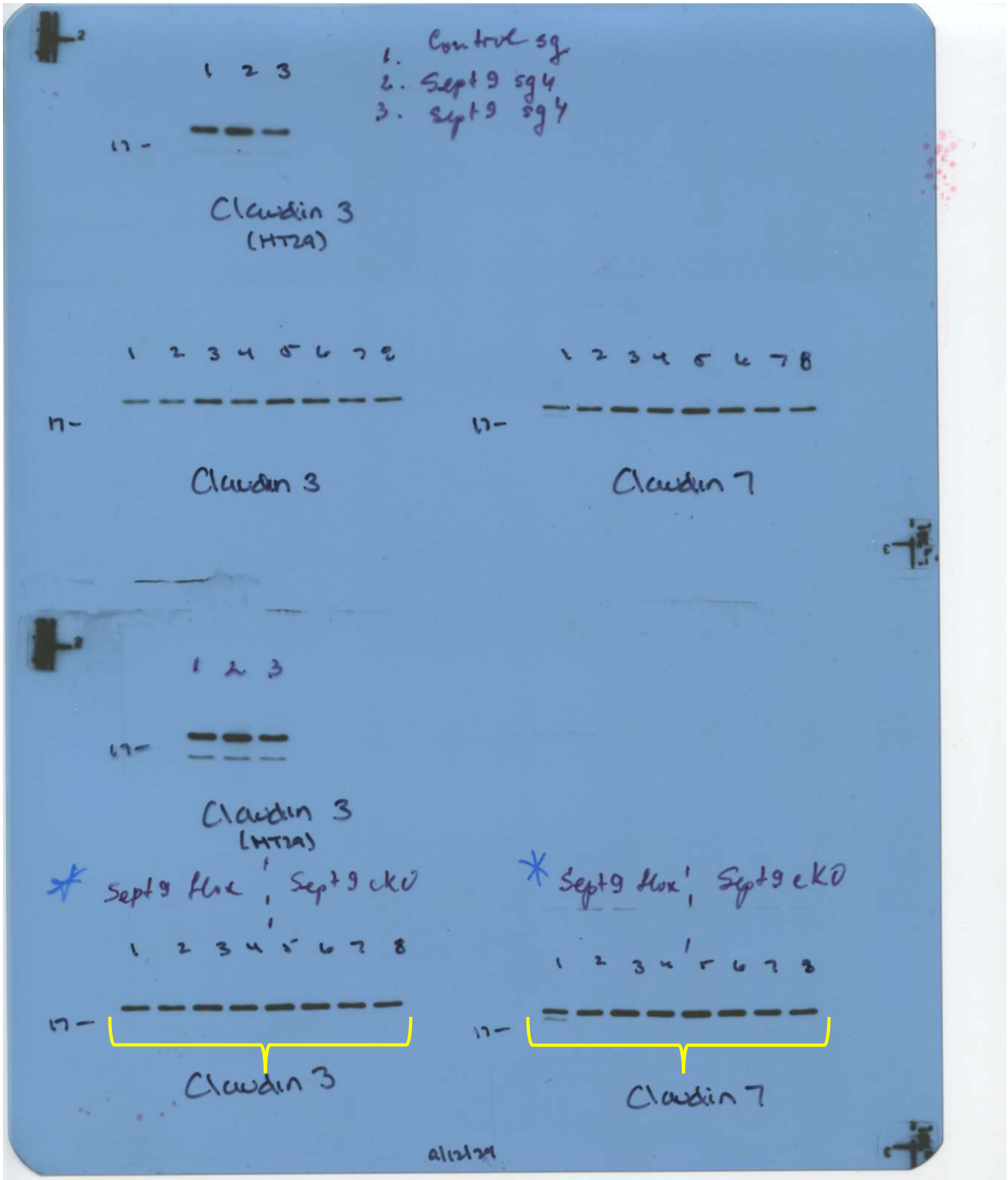

Full unedited gel for Fig.S3 C (Claudin3)
Antibody Thermo Fisher 34-1700

Full unedited gel for Fig.S3 C (Claudin7)
Antibody Thermo Fisher 34-9100

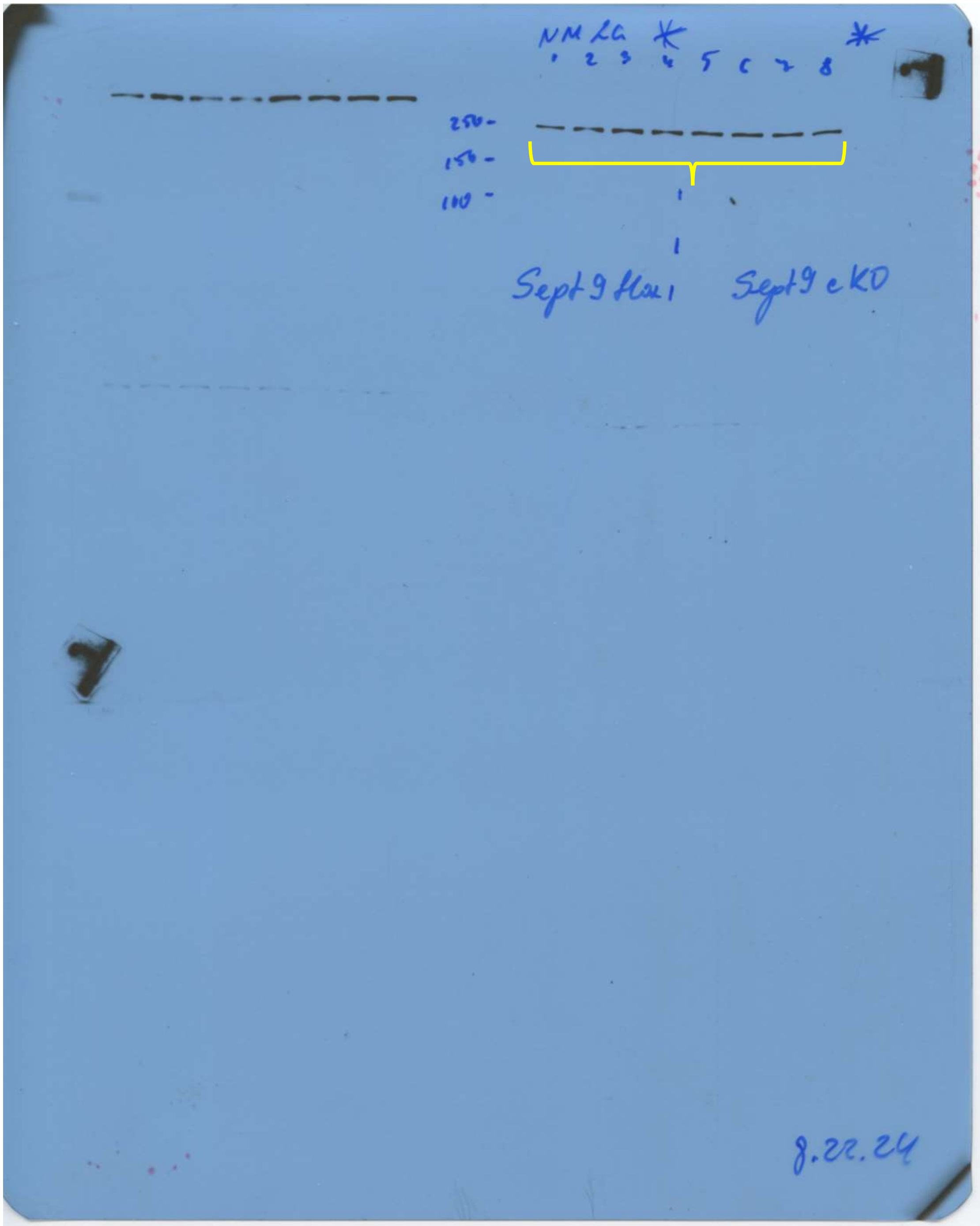

Full unedited gel for Figure S3 C
(NM IIA)
Antibody BioLegend 909801

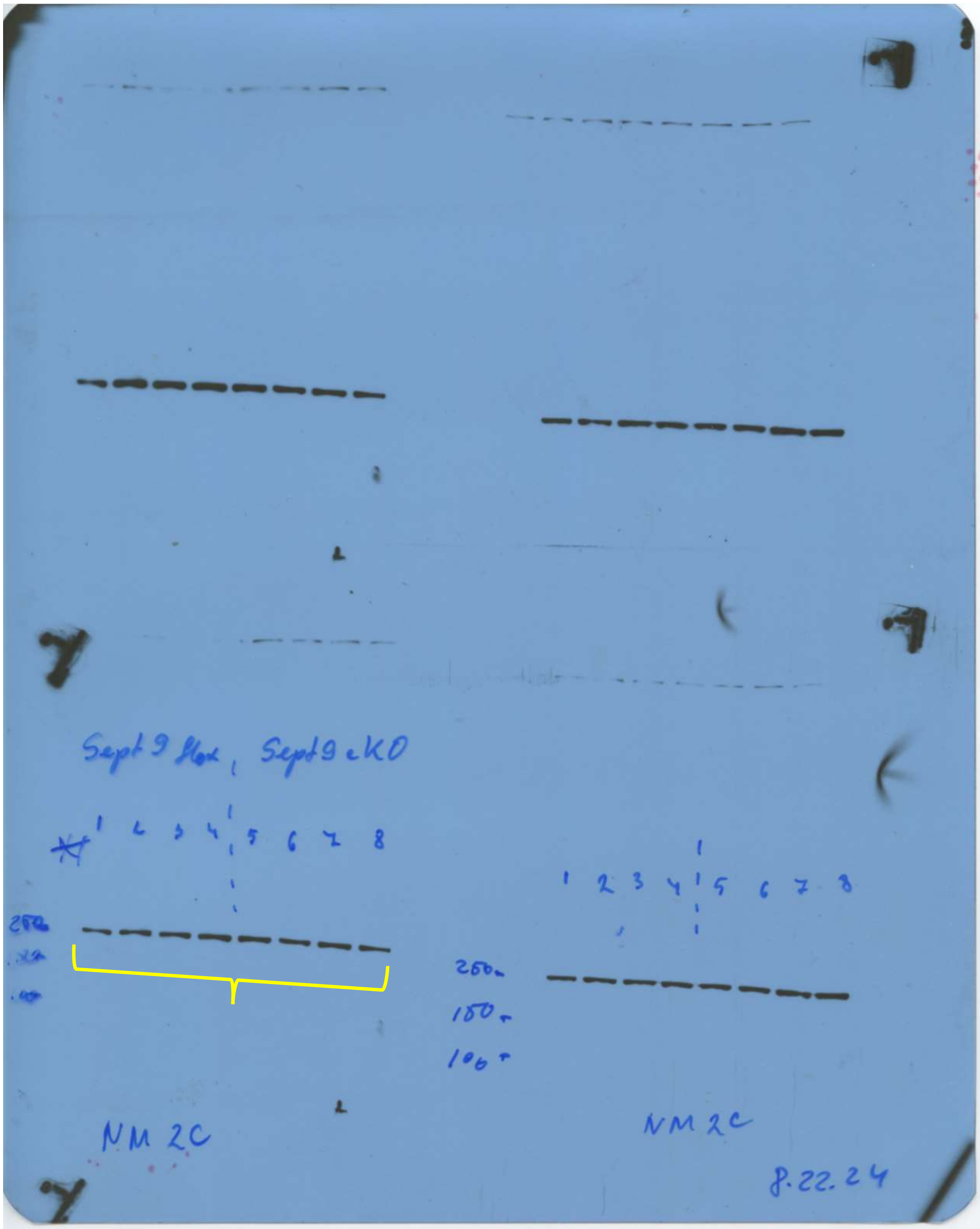

Full unedited gel for Figure S3 C
(NM IIC)
Antibody Cell Signaling
Technology 8189S

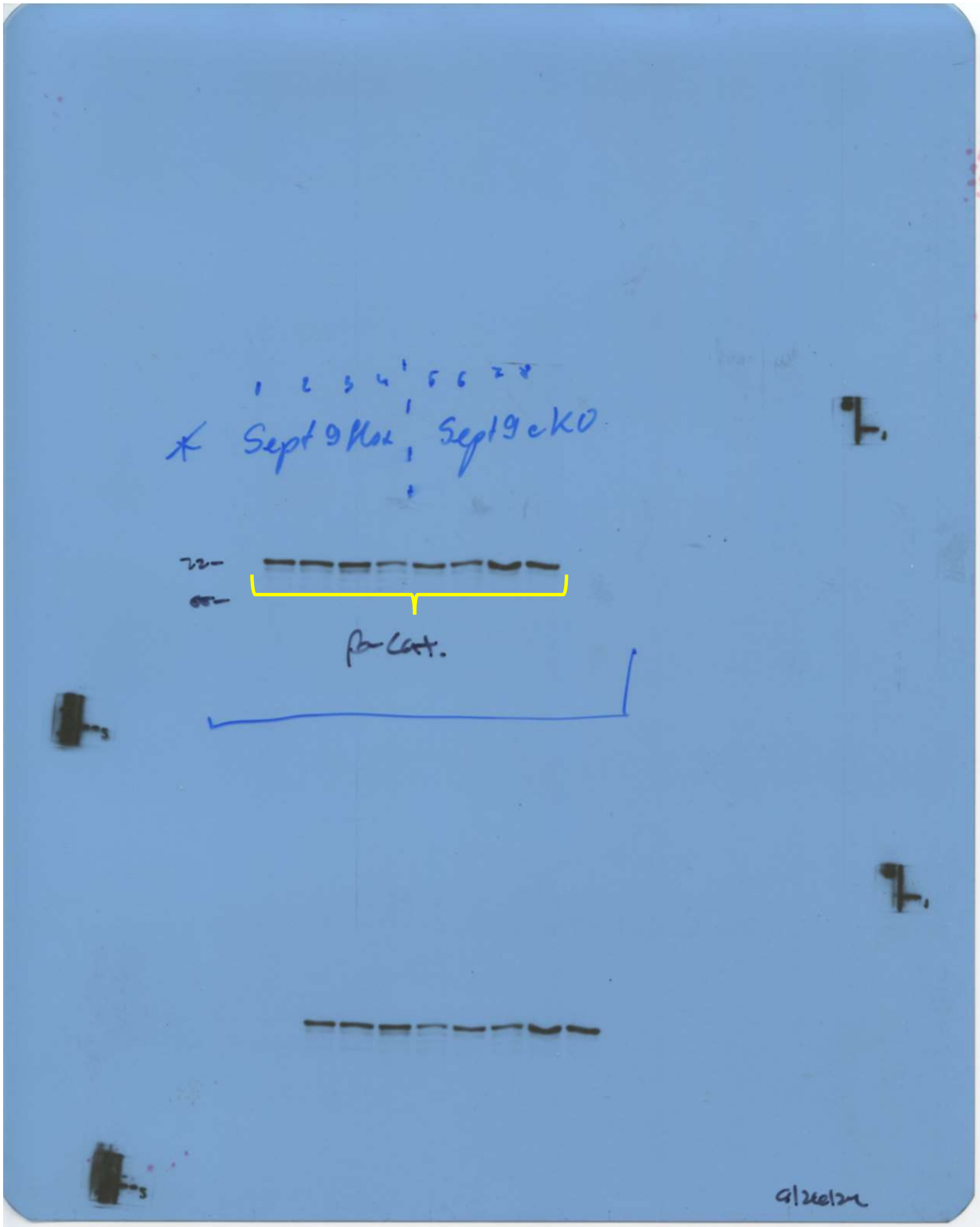

Full unedited gel for Figure S3 C
(β-catenin)
Antibody BD Biosciences 610153

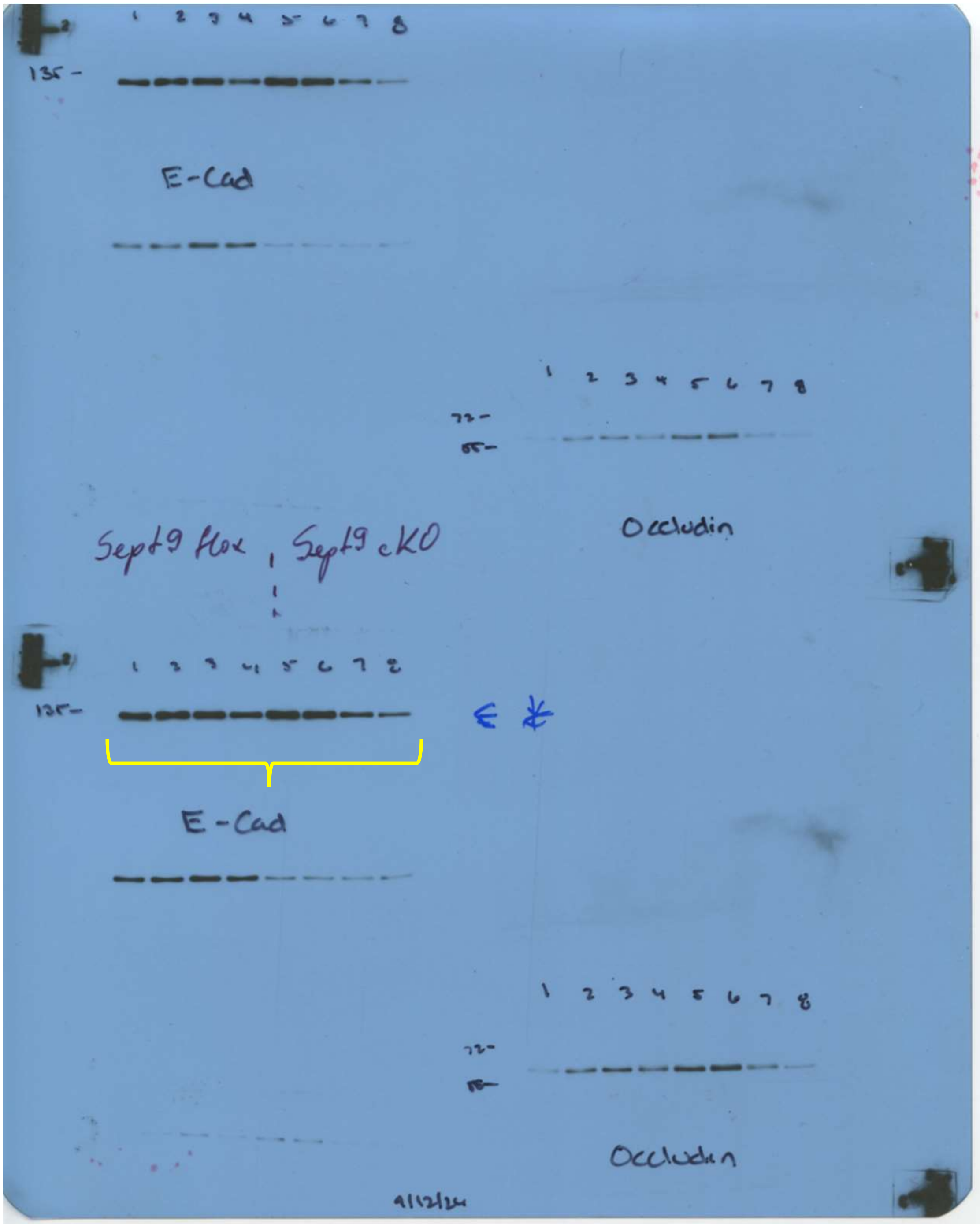

Full unedited gel for Figure S3 C
(E-cadherin)
Antibody BD Biosciences 610182

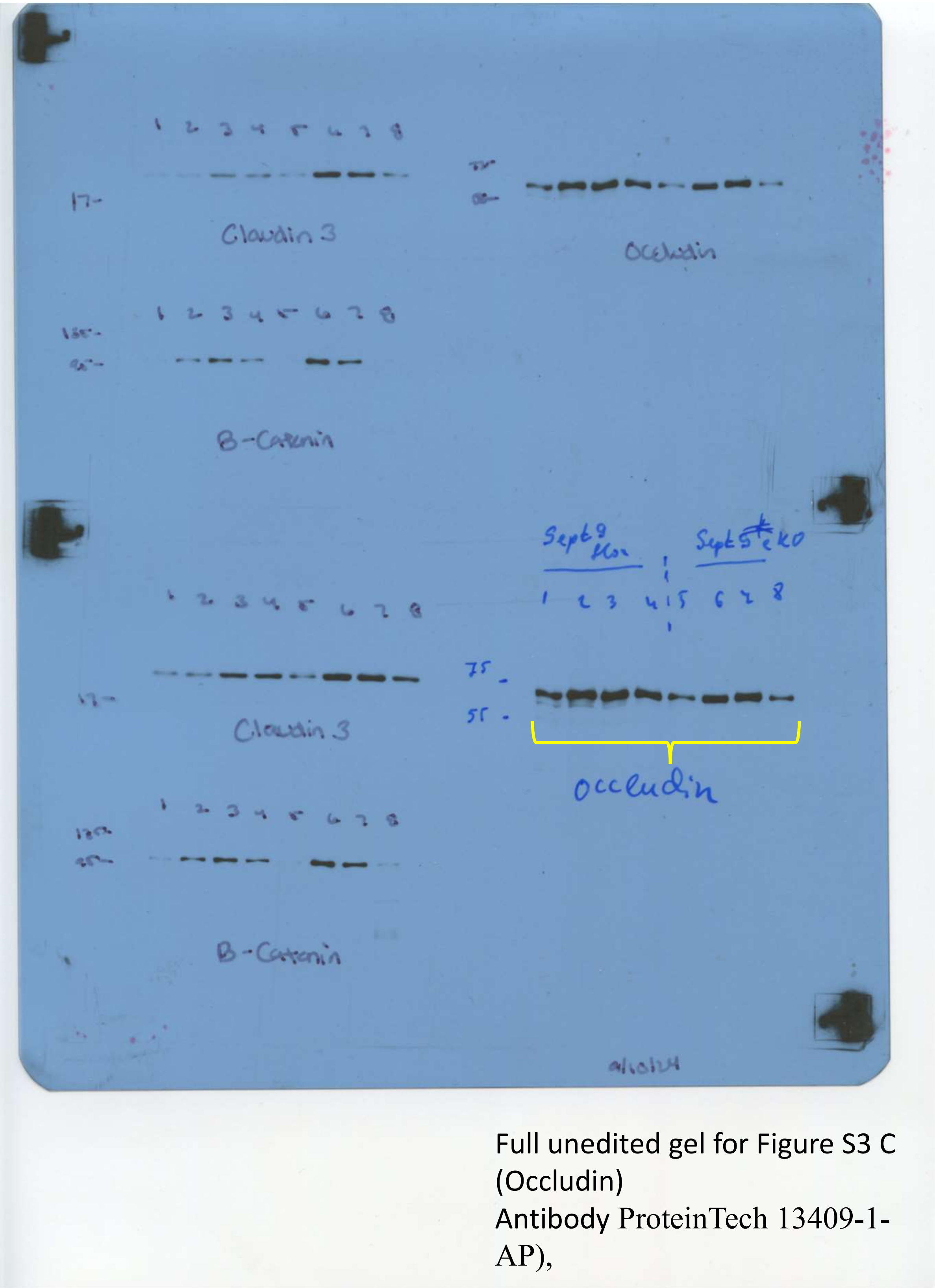

Full unedited gel for Figure S3 C (Occludin)
Antibody ProteinTech 13409-1-AP),

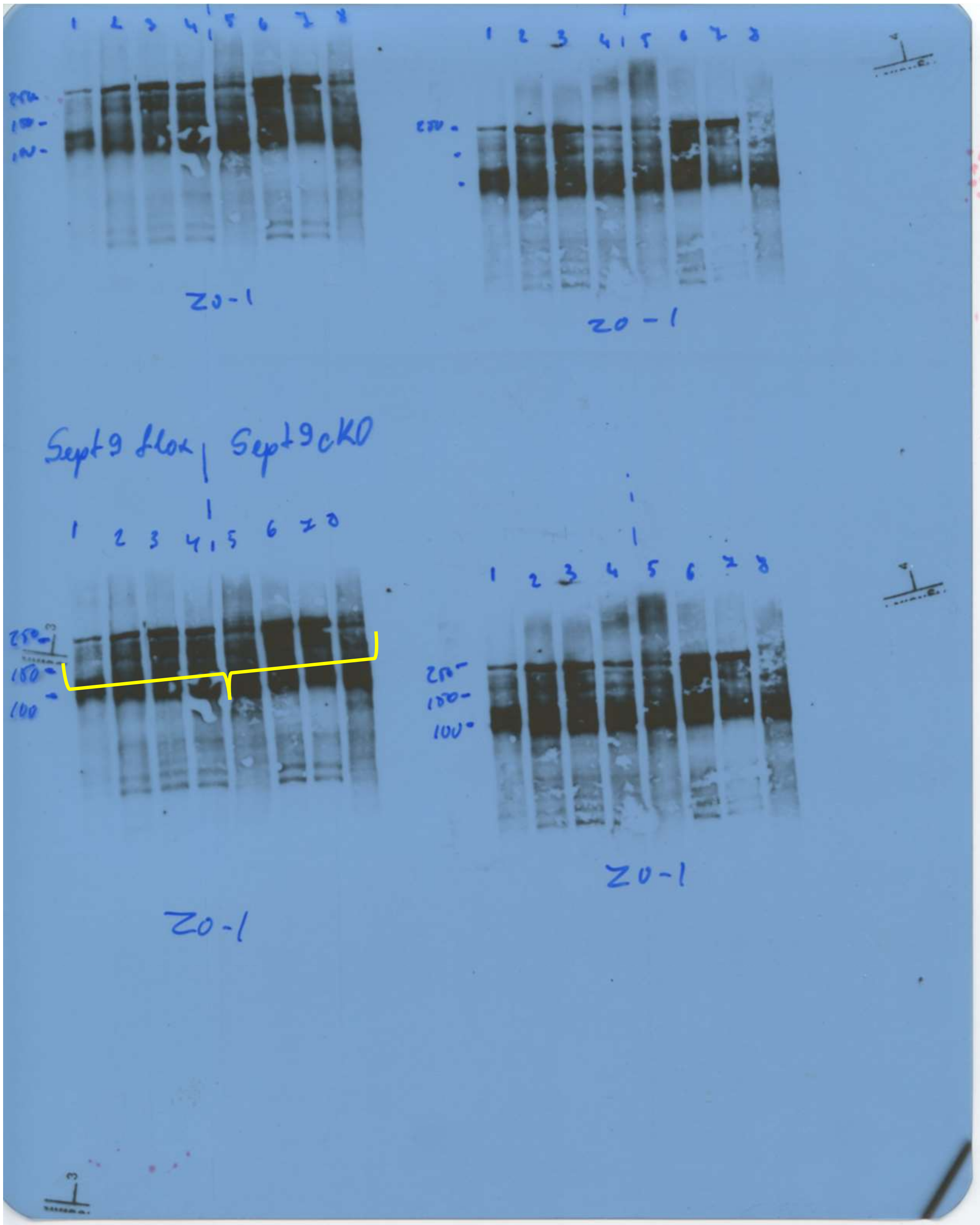

Full unedited gel for Figure S3 C (ZO-1)

Antibody ZO-1 (Invitrogen 40-2200),

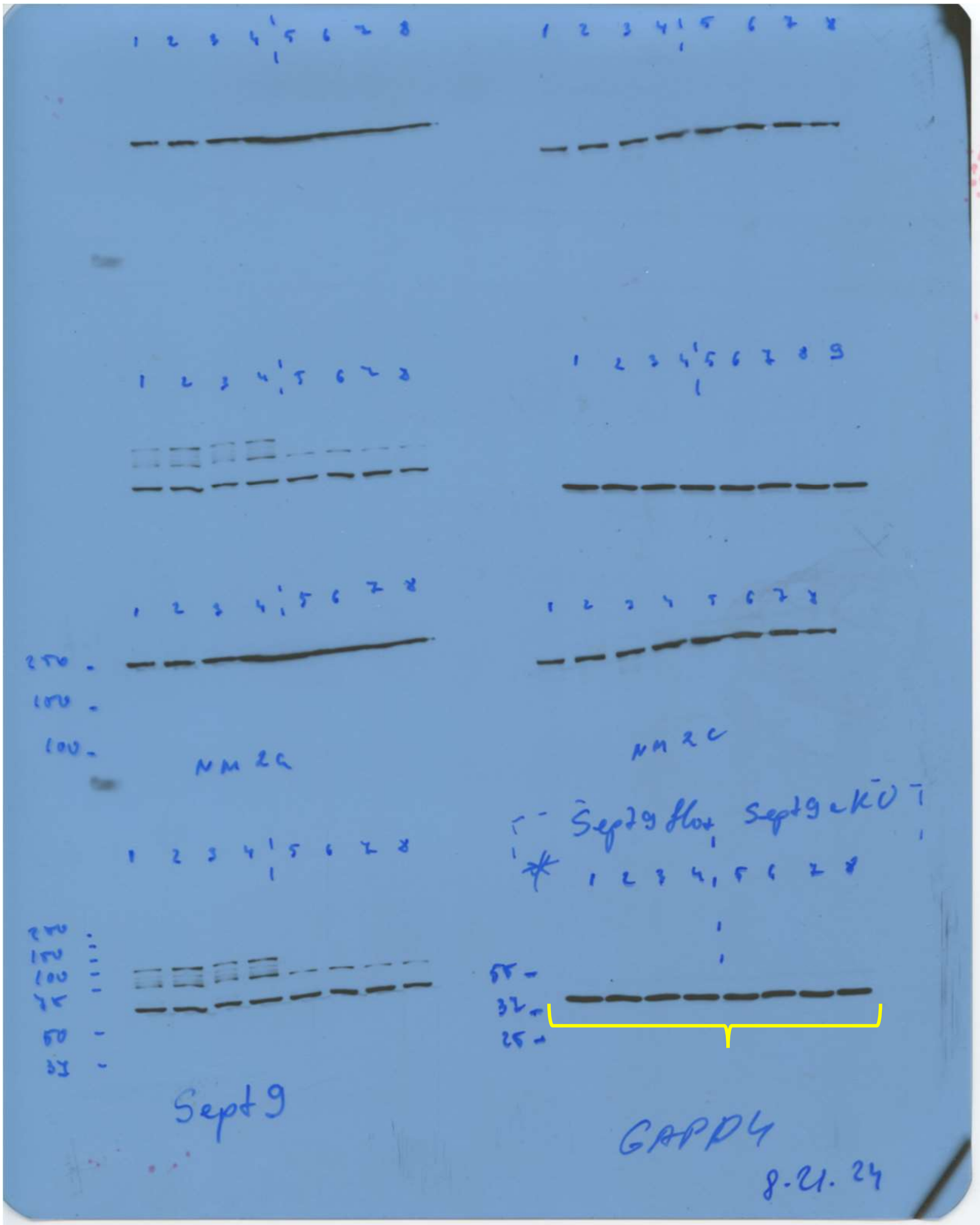

Full unedited gel for Figure S3 C (GAPDH)
Antibody Cell Signaling Technology 2118S

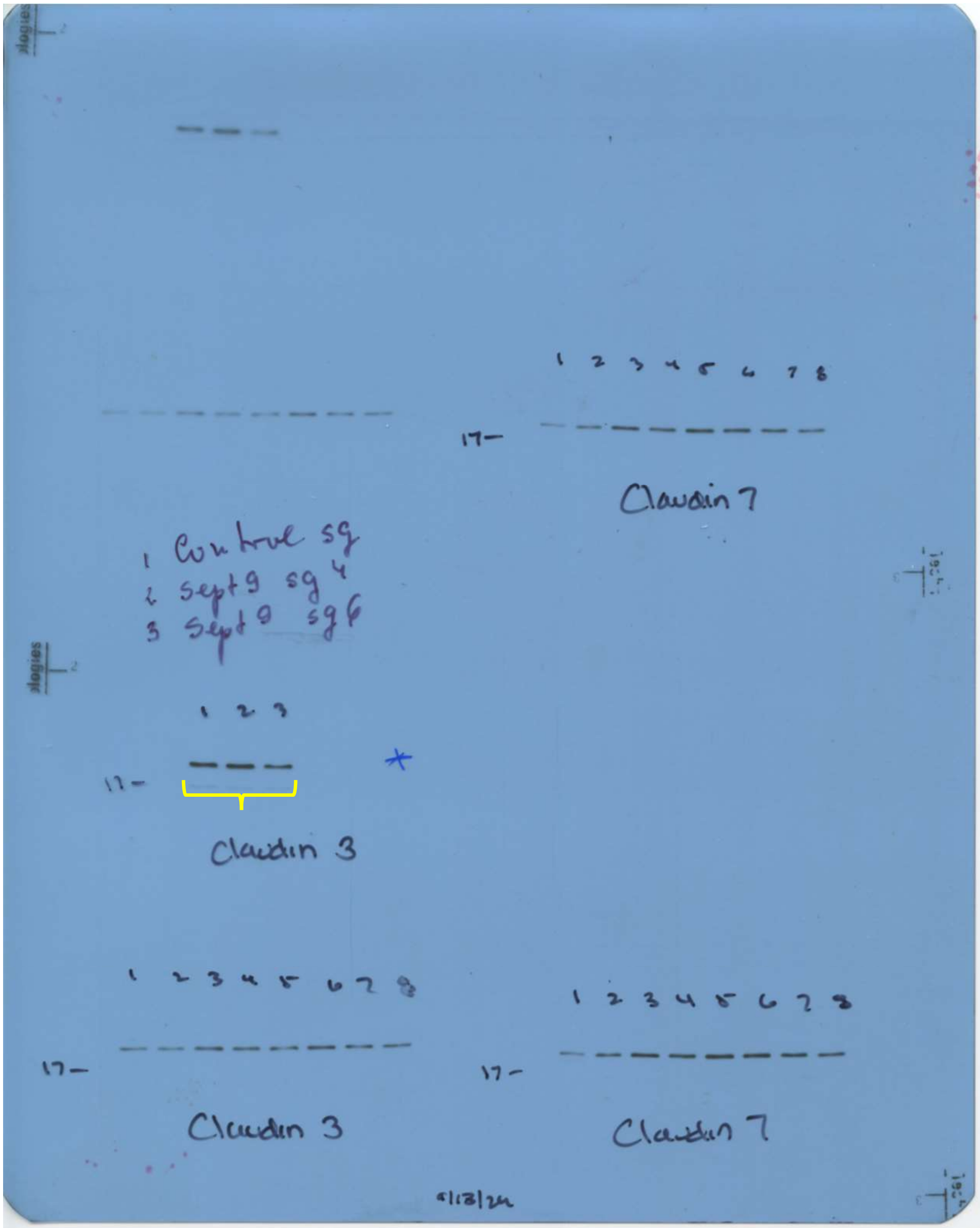

Full unedited gel for Fig.S5
A (Claudin3)
Antibody Thermo Fisher
34-1700

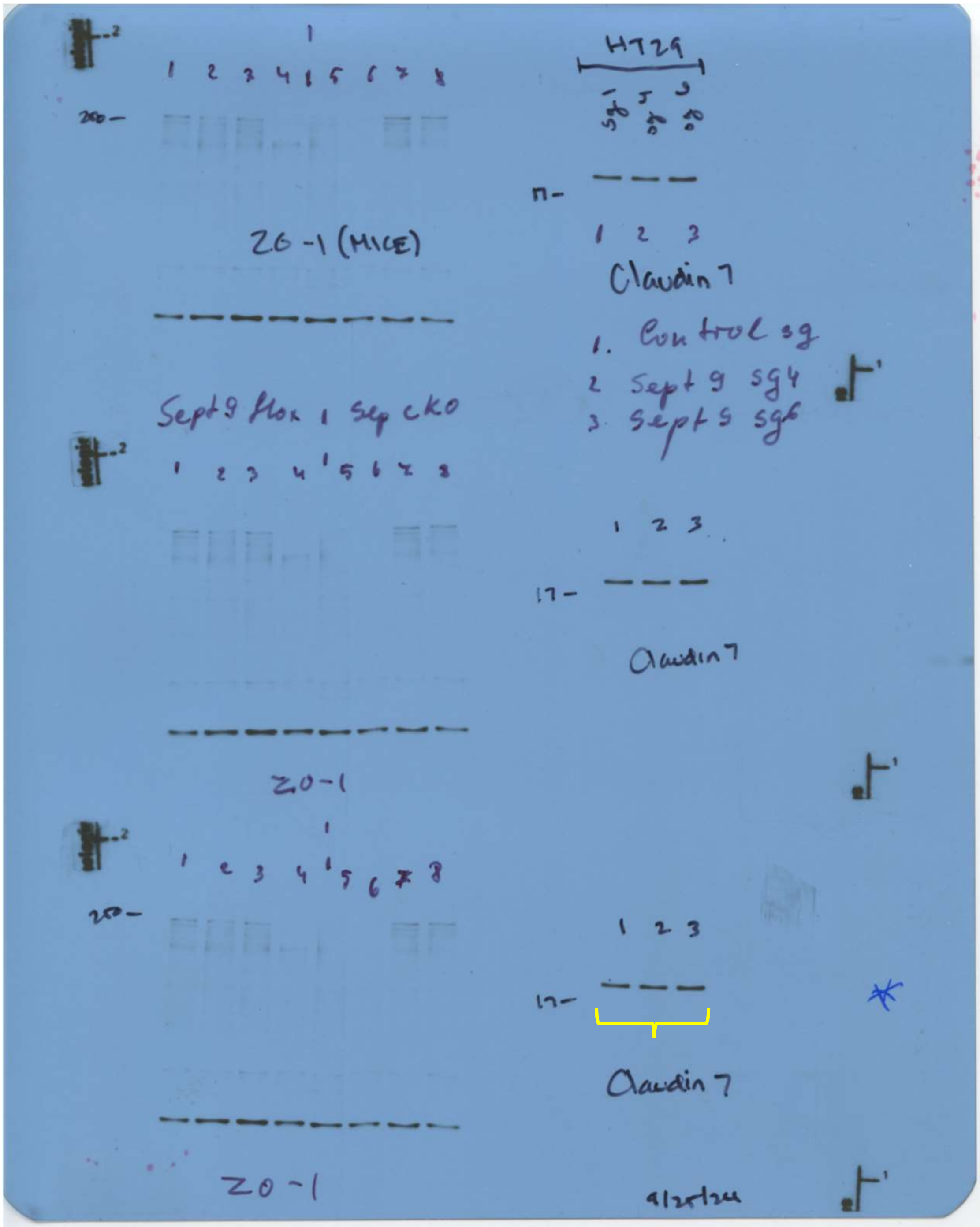

Full unedited gel for Fig.S5 A (Claudin7)
Antibody Thermo Fisher 34-9100

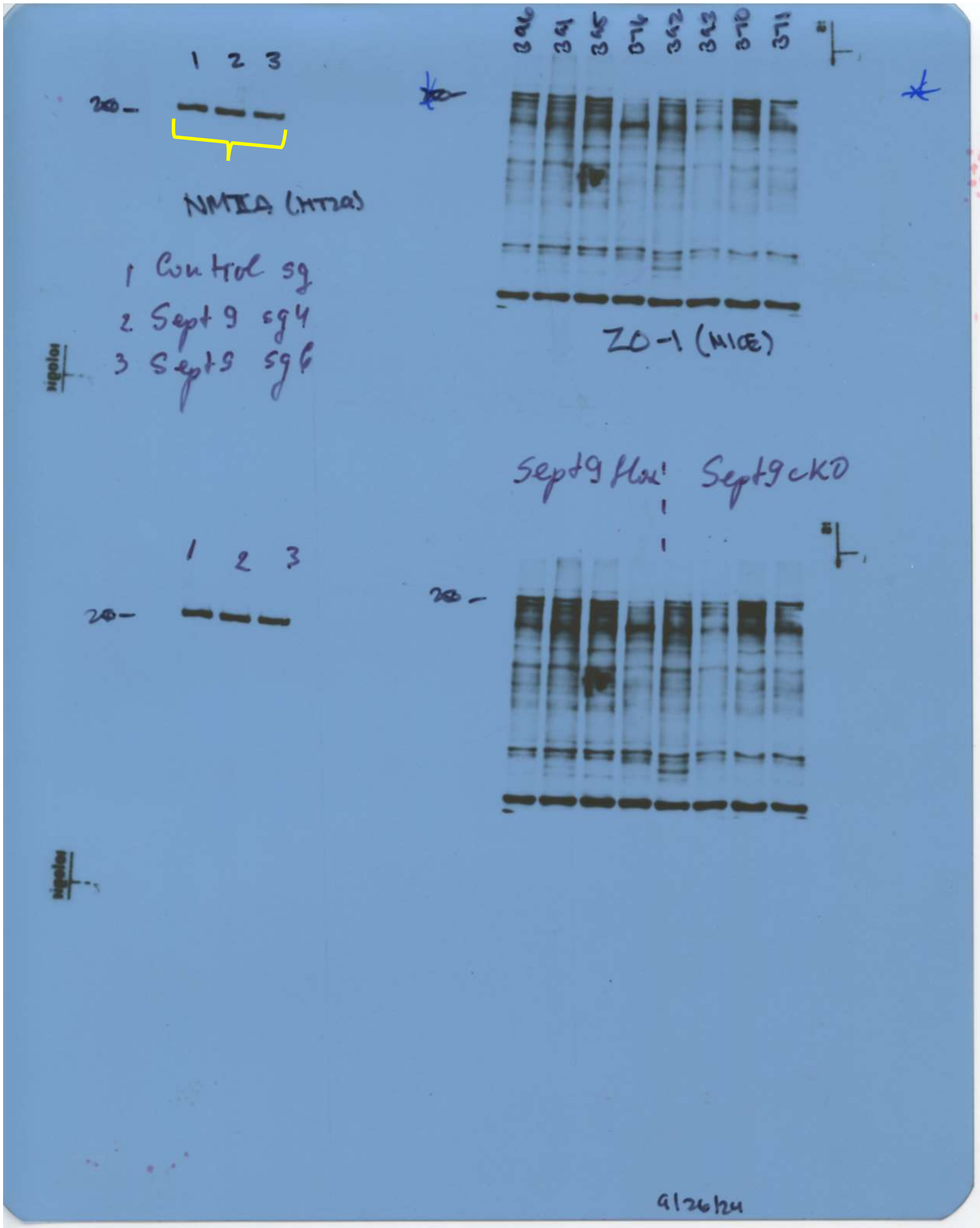

Full unedited gel for Figure S5 A
(NM IIA)
Antibody BioLegend 909801

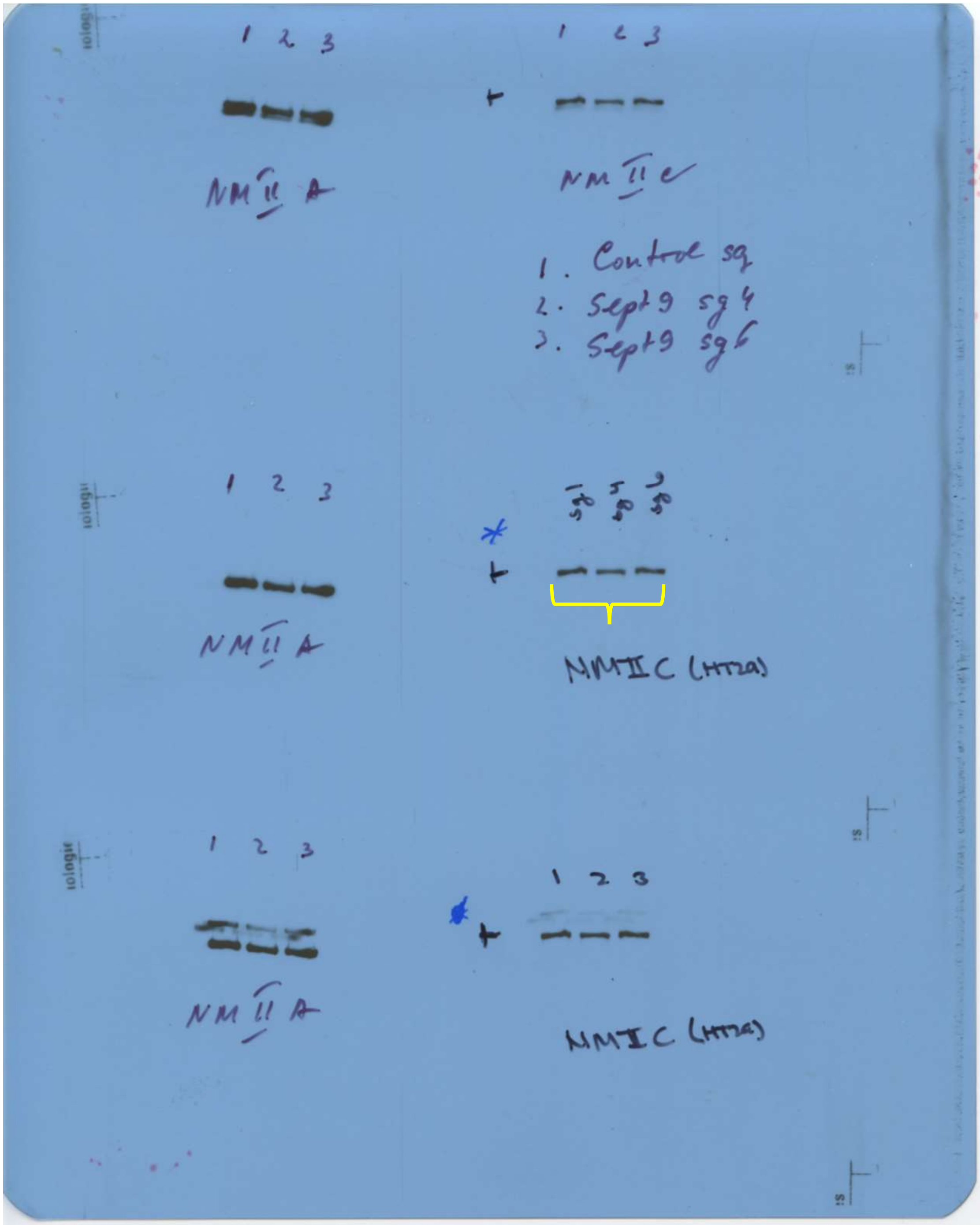

Full unedited gel for Figure S5 A (NM IIC)
Antibody Cell Signaling Technology 8189S

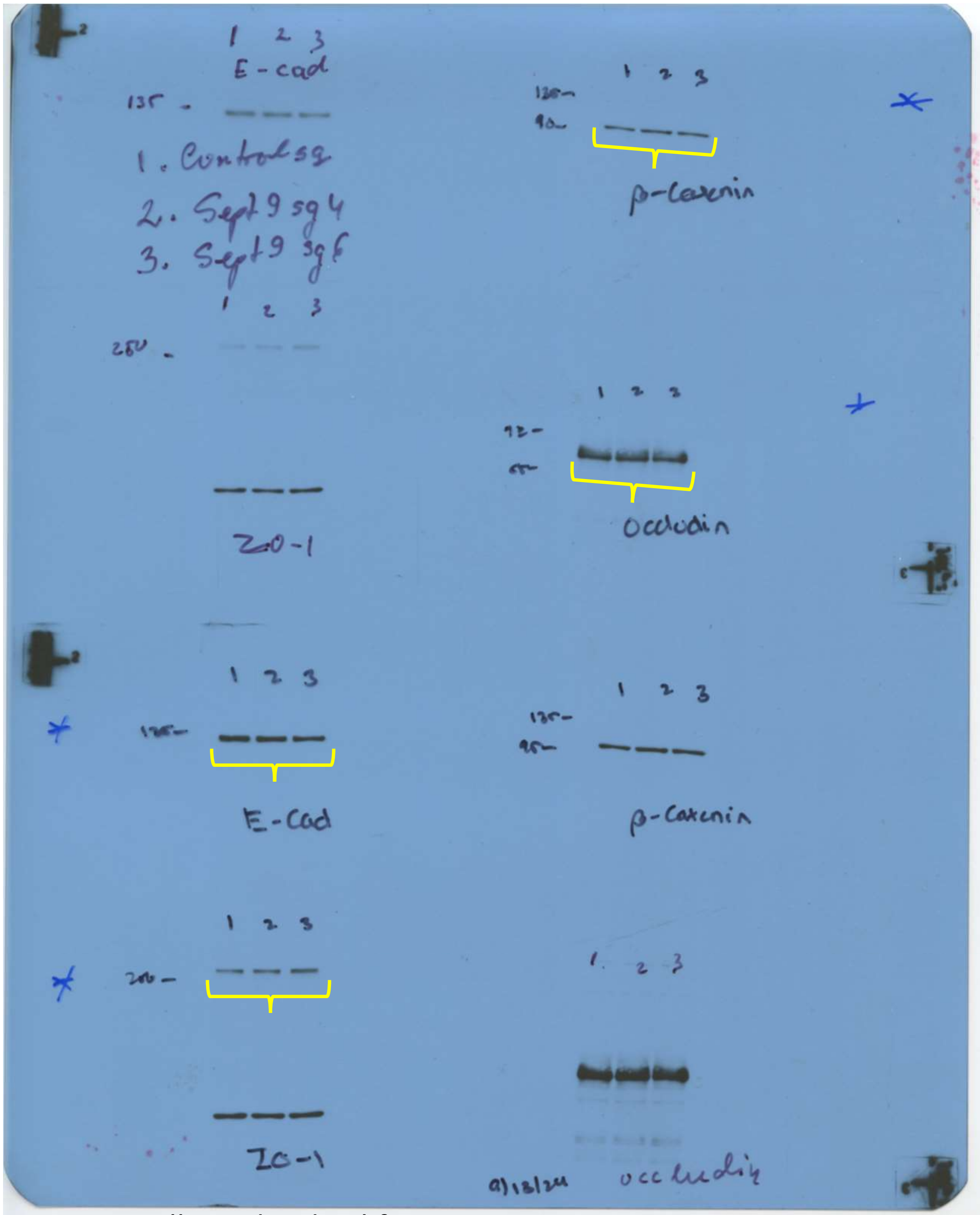

Full unedited gel for Figure S5 A
β-catenin-Antibody BD Biosciences 610153
E-cadherin-Antibody BD Biosciences 610182
ZO-1-Antibody Invitrogen 40-2200
Occludin-Antibody ProteinTech 13409-1-AP),

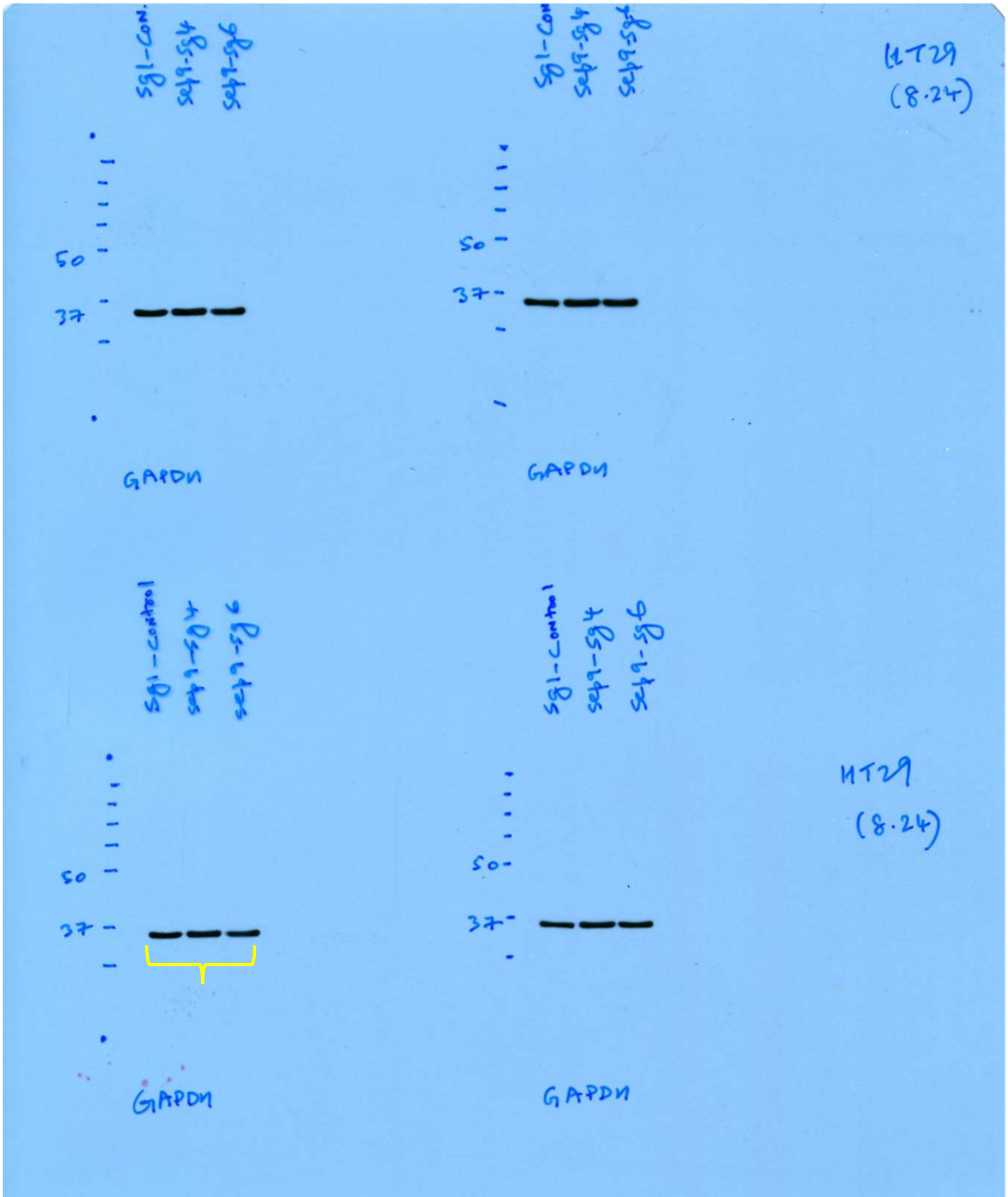

Full unedited gel for Figure S5 A (GAPDH)
Antibody Cell Signaling Technology 2118S
